# Herbicide Metabolic Resistance in *Poaceae* Plants via the GA‐GID1/DELLA‐DOF2‐P450s Module

**DOI:** 10.1002/advs.76742

**Published:** 2026-07-24

**Authors:** Junzhi Wang, Jiale Qi, Lin Wei, Qin Yu, Zhongyuan Liu, Lianyang Bai, Lang Pan

**Affiliations:** ^1^ College of Plant Protection Hunan Agricultural University Changsha China; ^2^ Hunan Plant Protection Research Institute Hunan Academy of Agricultural Sciences Changsha China; ^3^ Yuelushan Laboratory Changsha China

**Keywords:** DNA binding with one finger, echinochloa crus‐galli, herbicide resistance, gibberellins, penoxsulam, transcript regulation

## Abstract

Barnyard grass (*Echinochloa crus‐galli*) is one of the world's most important weeds, and the evolution of herbicide resistance in this species threatens rice and maize production. Cytochrome P450–mediated metabolic herbicide detoxification is a prevalent resistance mechanism, yet its upstream regulatory control has remained elusive. This study uncovers an important regulatory module linking gibberellin (GA) signaling to herbicide detoxification. We identify the DOF‐family transcription factor DOF2 as an important regulator that directly activates herbicide detoxification genes, including *CYP81A21* and *CYP704A371* in *E. crus‐galli*, and *CYP81A6* in rice. *DOF2* is constitutively elevated in resistant *E. crus‐galli* and further induced by herbicide treatment. Overexpression of *DOF2* in *E. crus‐galli* or its ortholog in rice confers resistance, while knockout of the rice ortholog increases susceptibility. Mechanistically, the DELLA protein SLR1 interacts with DOF2 to repress its transcriptional activity, and GA signaling relieves this repression. We further show that herbicide treatment rapidly upregulates GA biosynthesis genes, leading to increased GA levels that trigger this pathway. Importantly, this GA–GID1/DELLA–DOF2–P450 axis operates in at least two *Poaceae* species, suggesting a conserved regulatory logic. These findings establish a direct mechanistic link between phytohormone signaling and metabolic herbicide resistance, with implications for weed resistance management and crop tolerance improvement.

## Graphical abstract

Plant hormone signaling modulates herbicide resistance in *Echinochloa crus‐galli*. The GA–DELLA–DOF2 cascade directly activates P450 detoxification genes in two *Poaceae* species, uncovering a core mechanism of metabolic herbicide resistance.

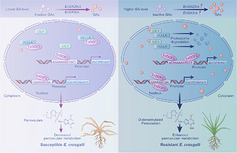

## 1 Introduction

Weeds are among the most significant constraints to sustainable crop production worldwide due to their strong adaptability to human‐managed agroecosystems [1]. The widespread and repeated use of herbicides as the primary weed control method has exerted intense selective pressure on weed populations, leading to the evolution of herbicide resistance and creating a growing challenge to global food security [2]. Herbicide resistance can be conferred by two major mechanisms: target‐site resistance (TSR) and non‐target‐site resistance (NTSR) [3]. TSR involves target‐site mutation, insertion and deletion, or gene copy number variation [3, 4, 5]. NTSR encompasses resistance mechanisms not related to the targets, including herbicide sequestration, limited translocation, and rapid metabolism (metabolic herbicide resistance) [2]. Compared to TSR, most genes associated with NTSR belong to large superfamilies, complicating the identification of specific resistance genes [6]. Consequently, only a limited number of NTSR genes have been identified and characterized [7, 8, 9, 10, 11].

Among NTSR mechanisms, metabolic herbicide resistance poses the greatest threat to agriculture, as metabolism‐based resistant plants often exhibit cross‐resistance to multiple herbicides of different chemical classes and modes of action [2]. A key enzyme family implicated in this process is cytochrome P450 monooxygenases (P450s), which play a central role in the metabolism of a wide range of natural and synthetic xenobiotics, including herbicides [2, 3, 12]. Similarly, herbicide selectivity in crop species (e.g., rice, maize, wheat) is often due to more rapid herbicide metabolism involving P450s than weedy species [13, 14, 15]. Due to the challenges in studying the extensive P450 families and the lack of well‐annotated weed genomes, discovery of specific P450 genes involved in metabolic herbicide resistance in weedy plant species has been slow until the milestone study of map‐based cloning of the major P450 gene *CYP81A6* in rice, endowing tolerance to PSII‐ and ALS‐inhibiting herbicides [13]. In light of this work, the two P450 genes in *Echinochloa phyllopogon*, *CYP81A12* and *CYP81A21*, were first cloned and characterized exhibiting broad cross‐resistance to multiple herbicide chemistries and modes of action, including the ALS‐inhibiting herbicides penoxsulam and bensulfuron‐methyl [9, 16]. Since then, several P450 genes mainly in the 81, 704, and 709 families (e.g., *CYP81A10v7*, *CYP81A68*, *CYP81Q32*, *CYP704C1*, *CYP704A177*, *CYP709C69*, *CYP709C56*) have been identified in a few weedy grass species [8, 17, 18, 19, 20, 21, 22, 23, 24, 25].

In almost all reported cases so far, the resistance is due to overexpression of identified P450 genes. However, how gene overexpression is regulated genetically or epigenetically is largely unknown. Only limited studies are available. For example, we identified epigenetic regulation (DNA methylation) may play a role in *E. crus‐galli CYP81A68* gene expression for penoxsulam resistance [24]. Other studies showed *EcMETTL7A* mediates DNA methylation of the *EcCYP72A385* promoter, regulating its expression in florpyrauxifen‐benzyl resistance in *E. crus‐galli* [25]. In addition, evidence for transcription factors (TFs) regulating resistance‐associated P450s in insecticide resistance have been reported [26, 27, 28, 29], and such regulation remains poorly understood in herbicide metabolic resistance [30] in weeds and in herbicide tolerance in crop species. For instance, bZIP88 TFs from *E. crus‐galli* and *E. glabrescens* have been implicated in resistance to three different herbicides, but the downstream target genes are yet to be identified [31]. Our recent studies have identified the TF *BsTGAL6* in *Beckmannia syzigachne* as a regulator of *BsCYP81Q32* expression in resistance to ALS‐inhibiting herbicides [19].

The plant‐specific DOF (DNA‐binding with one finger) family is defined by a conserved C2–C2 zinc finger DNA‐binding domain and preferentially recognizes the core cis‐regulatory element 5′‐(A/T)AAAG‐3′ in the promoters of target genes. DOF proteins participate in diverse biological processes, including seed germination, flowering, fruit development, hormone signaling, and responses to a range of abiotic and biotic stresses such as salinity, drought, cold, heat, and pathogen attack [32, 33, 34, 35, 36, 37]. Previous studies have shown that DOF gene expression can be induced by abiotic stress [38] and phytohormones [39, 40]. However, precise functions of many DOF family members remain largely unknown, let alone their regulatory networks and underlying mechanisms. Elucidating the functional diversity of this plant‐specific TF family is essential for better understanding of stress response mechanisms in plants. To date, it remains unclear whether DOFs are involved in herbicide response or resistance regulation in plants. Moreover, their biological functions and molecular regulatory mechanisms in weedy species remain largely unexplored.

Gibberellin acids (GAs) are known to regulate diverse aspects of plant growth and development, including seed germination, hypocotyl elongation, leaf expansion, and the floral transition [41, 42]. GA biosynthesis and signaling pathways have been extensively studied in the model species *Arabidopsis thaliana* and rice. Notably, as a key regulatory element in fruit set initiation, *DOF10* expression is transcriptionally regulated by GA during reproductive development [43, 44]. The core of GA signaling is the perception of active GA by its receptor, GIBBERELLIN INSENSITIVE DWARF1 (GID1), which undergoes a conformational change upon GA binding. This enables the formation of a GA‐GID1‐DELLA complex with DELLA protein SLENDER RICE 1 (SLR1), which are key repressors of GA responses [41]. SLR1 control the activity of many TFs via protein–protein interactions, and their degradation under GA signaling significantly impacts the cell's transcriptional landscape [45]. GID1 was first identified in rice, and its knockout *gid1* mutants displayed a characteristic dwarf phenotype with reduced sensitivity to GA [46]. Loss‐of‐function mutations in GA biosynthesis *GA20ox* or *GA3ox* genes result in diminished levels of bioactive GAs, leading to DELLA accumulation [47]. Nevertheless, little information is available on how these above pathway(s) mediating herbicide metabolic resistance genes.

*Echinochloa* spp. are the most prevalent weeds in global rice production [48]. *E. crus‐galli*, a polyploid and highly invasive species, is the most malignant species infesting rice paddies [49]. Global rice yield losses caused by competition with *E. crus‐galli* are estimated at ∼35% and can be as high as 80% [50]. *E. crus‐galli* control has been relied heavily on chemical herbicides, particularly the acetolactate synthase (ALS)‐inhibiting herbicides such as penoxsulam. However, excessive and repetitive use of the herbicide has resulted in a surge in herbicide resistance evolution in *E. crus‐galli* [24, 51, 52]. In addition to target‐site ALS mutations [53], non‐target‐site metabolic resistance to penoxsulam is also prevalent [24, 25, 31, 54]; however, progress in identifying resistance‐endowing P450 genes remains limited [24]. As the metabolic resistance and involving genes in weedy species often mimic the competing crop species, therefore, identifying these genes and elucidating their regulatory mechanisms are important both for understanding resistance, evolution, and management in weeds and for herbicide tolerance breeding and improvement in crops.

In this study, we identified DOF2 in herbicide resistant *E. crus‐galli* and herbicide tolerant rice (rice is naturally tolerance to penoxsulam) as a key TF endowing resistance/tolerance to ALS‐ and ACCase‐inhibiting herbicides by regulating the downstream gene expression of *CYP81A21*/*EcCYP704A371* (in *E. crus‐galli*), and *CYP81A6* (in rice), thereby enhancing herbicide detoxification. We further investigated the upstream regulation of DOF2 and demonstrated that GA signaling modulates DOF2 activity positively via *GID1* and negatively via DELLA. This establishes a shared regulatory cascade GA‐GID1/DELLA‐DOF2‐CYPs for herbicides detoxification in weedy and crop species. Altogether, our findings uncover a hormone‐transcription‐metabolism axis, providing in‐depth information for genetic abiotic stress adaptation in plants, and diverse options and insights on resistance evolution and management in weeds and tolerance breeding in crops.

## 2 Results

### 2.1 EcDOF2 Acts as a Nuclear Transcriptional Activator Linked to Herbicide Resistance in *E. crus‐galli*

**FIGURE 1:**
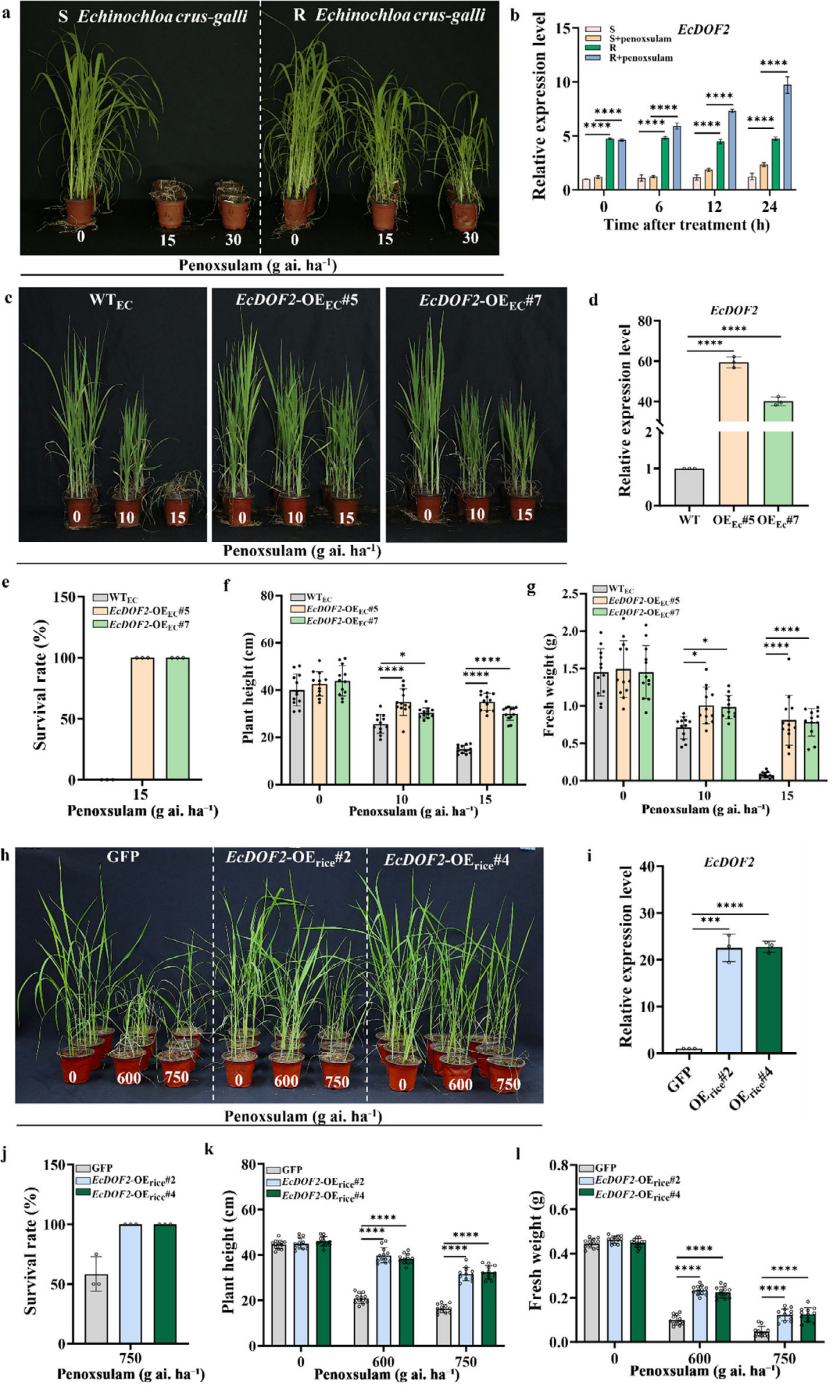
Overexpression of *EcDOF2* endows resistance to penoxsulam in *Echinochloa crus‐galli* and enhances tolerance in rice. (a) Penoxsulam resistance in the resistant (R) versus susceptible (S) populations (the filed recommended rate is 15 g ai. ha^−1^) 21 days after treatment. (b) RT‐qPCR quantification of *EcDOF2* expression in R and S plants following penoxsulam treatment (10 g ai. ha^−^^1^). (c) Phenotypic comparison of WT_EC_ and *EcDOF2*‐OE *E. crus‐galli* lines (OE_EC_#5 and OE_EC_#7), 21 days after penoxsulam treatment. (d). RT‐qPCR quantification of *EcDOF2* expression levels in *E. crus‐galli* OE_EC_#5 and OE_EC_#7 lines relative to WT_EC_ *E. crus‐galli*. (e–g) Survival rate (e), plant height (f), and fresh weight (g) of WT_EC_ and *EcDOF2*‐OE_EC_ lines responding to penoxsulam treatment in (c). (h) Phenotypes of GFP control and *EcDOF2*‐OE rice lines (OE_rice_#2 and OE_rice_#4) 21 days after penoxsulam treatment. (i) RT–qPCR quantification of *EcDOF2* transcript levels in transgenic rice lines OE_rice_#2 and OE_rice_#4 compared to GFP. (j–l) Survival rate (j), plant height (k), and fresh weight (l) of GFP and *EcDOF2*‐OE_rice_ lines following penoxsulam treatment in (h). Two‐tailed Student's t‐test was performed for (d, i). Two‐way ANOVA followed by Tukey's multiple comparison test was performed for (b). Two‐way ANOVA followed by Dunnett's multiple comparison test was performed for (f, g, k, l). Data are means ± SD (*n* = 12) in (c, h). Asterisks indicate significant differences compared with WT_EC_ or GFP control (*p < 0.05, ***p < 0.001, ****p < 0.0001).

Our previous work showed that the resistant (R) population of *E. crus‐galli* exhibits metabolic resistance to cyhalofop‐butyl [54], and further whole‐plant assays revealed non‐target‐site (no ALS target site mutations) cross‐resistance to penoxsulam with resistance index about 10 (Figure 1a and Table S1). Moreover, the known P450 inhibitor, malathion, significantly restored penoxsulam susceptibility (Table S1), suggesting possible metabolic resistance via P450‐mediate herbicide detoxification. In gene discovery for metabolic resistance to penoxsulam, we reanalyzed previously published RNA‐seq data from R and susceptible (S) populations of *E. crus‐galli*, and identified 11 TFs which were significantly upregulated (|log_2_FC| ≥ 1, FDR < 0.05) in the R population, encompassing members of the bZIP, DOF, GATA, MAD, MYB, and NAC families (Figure S1a,b and Table S2), as well as 27 P450 genes (Table S3). Based on these and our previous work on TF [19], we hypothesized that a cross talk between TF and P450s may be responsible for herbicide detoxification and thus herbicide resistance in this species.

Among these identified TFs, *EcDOF2* displayed 5‐fold higher expression (the highest) in R than in S plants under non‐treated conditions, as measured using reverse transcription quantitative PCR (RT‐qPCR) (Figure S1c). When R and S plants were treated with penoxsulam (10 g ai. ha^−^^1^), time‐course analysis revealed that *EcDOF2* expression in R plants further increased significantly from 6 h onward and near twofold above the untreated R plants at 24 h (Figure 1b). In contrast, S plants maintained lower transcript levels, with only a slight increase overtime. Higher constitutive and inducible upregulation of *EcDOF2* in R plants suggests its possible roles in transcriptional regulation of resistance.

Using genomic and transcriptomic resources of *E. crus‐galli*, we cloned the full‐length coding sequences of *EcDOF2* from R and S plants, with 1710 bp, encoding 569 amino acids. As only synonymous single nucleotide polymorphisms (SNPs) were detected (Figure S1d), *EcDOF2* expression levels, rather than SNPs, are likely responsible for resistance regulation. Furthermore, alignment of the 2000 bp promoter sequences of *EcDOF2* revealed no differences between S and R plants (Figure S2).

To investigate the evolutionary relationship of *EcDOF2* to other plant DOFs, we conducted multiple sequence alignments and phylogenetic analysis using 162 DOF protein sequences from five plant species, including *A. thaliana* (36), *O. sativa* (30), *Sorghum bicolor* (27), *Setaria viridis* (35), and *Setaria italica* (34). Among these, EcDOF2 share 77.1% sequence identity with OsDOF2 (*O. sativa*) (Figure S3a,b). Based on the close crop‐weed association, and sequence identity, conserved domain architecture, as well as phylogenetic position, this EcDOF family member was designated EcDOF2. Detailed sequence alignments were provided in Data S1.

Domain analysis revealed that the N‐terminal region of EcDOF2 harbors a conserved zinc finger motif, characteristic of the DOF DNA‐binding domain, while the C‐terminal region contains a putative transcriptional activation domain (Figure S3c). To analyze the subcellular localization of EcDOF2, we transiently expressed the EcDOF2‐GFP fusion protein in *Nicotiana benthamiana* leaf epidermal cells. Confocal microscopy showed that GFP fluorescence was exclusively localized in the nuclear, whereas the control 35S::GFP signal was distributed throughout the cytoplasm and nucleus (Figure S3d).

To further determine the activity of EcDOF2 on transcriptional activation, we constructed three protein fragments: the full‐length sequence (EcDOF2, amino acids 1–569), the N‐terminal domain (EcDOF2‐N, amino acids 1–273), and the C‐terminal domain (EcDOF2‐C, amino acids 274–569). The result showed that only cells expressing *EcDOF2‐C* proliferated on the selective SD/‐Trp‐His‐Ade medium and produced blue colonies on X‐α‐gal‐containing plates, indicating that the C‐terminal region possesses transcriptional activation capability (Figure S3e). These results demonstrated that *EcDOF2* functions as a nuclear transcription factor, with the C‐terminal region mediating transcriptional activation.

### 2.2 Expression of *EcDOF2* in *E. crus‐galli* and Rice, and *OsDOF2* (*EcDOF2* Ortholog) in Rice, Confer Penoxsulam Resistance/Tolerance

To explore whether the EcDOF2‐mediated metabolic detoxification system identified in *E. crus‐galli* represents a conserved mechanism in poaceae, we first characterized *EcDOF2* in its native host. Using a newly established Agrobacterium‐mediated homologous transformation system in *E. crus‐galli* and introduced a Ubi::*EcDOF2* overexpression construct into calli derived from the S plants (Figure S4a–e). Hygromycin‐resistant T_0_ lines were verified by PCR and further confirmed by RT–qPCR (Figure S4f,g). Notably, this is the first successful generation of transgenic *E. crus‐galli* lines, a significant technical advance for functional studies in a major weedy species. Further efforts to establish a genome‐editing compatible system achieved little success.

To evaluate herbicide response, the 3–4 leaf stage WT_EC_ (*E. crus‐galli*) and *EcDOF2*‐overexpressing *E. crus‐galli* (*EcDOF2*‐OE_EC_) seedlings were foliar treated with the recommended field rate of penoxsulam (10 g ai. ha^−^^1^) and a higher rate (15 g ai. ha^−^^1^) (Figure 1c,d). Consequently, *EcDOF2‐*OE_EC_ *E. crus‐galli* plants exhibited significantly higher survival, plant height, and biomass than WT_EC_ *E. crus‐galli* (Figure 1e–g). These results demonstrate that *EcDOF2* overexpression confers penoxsulam resistance in *E. crus‐galli*. However, *EcDOF2*‐OE_EC_ plants exhibited partial rescue of herbicide tolerance (at 15 g ai. ha^−1^) with 45% fresh weight inhibition compared to 23% in the R biotype and 96% in wild‐type S plants (Figure S5a,b). These results indicate that *EcDOF2*‐mediated detoxification contributes to resistance, but does not fully recapitulate the high‐level resistance of the R population, suggesting that additional unidentified mechanisms operate in parallel to achieve the full resistance phenotype.

In addition, we generated transgenic rice lines expressing *EcDOF2* (Figure 1h). Two independent lines (OE_rice_#2 and OE_rice_#4) were selected for further analysis (Figure 1i). Under penoxsulam treatment (600 and 750 g ai. ha^−^^1^, rates achieving rice control due to natural tolerance), *EcDOF2*‐OE_rice_ lines exhibited greater plant height and biomass compared with GFP controls (Figure 1j–l). Together, these results demonstrate that *EcDOF2* enhances penoxsulam resistance/tolerance in *E. crus‐galli* and rice, indicating a conserved regulatory role for this TF in herbicide stress response.

To investigate whether the function of *EcDOF2* is conserved in the crop species rice, we identified the *EcDOF2* ortholog gene in rice, *OsDOF2* (LOC_Os01g15900), a TF localized in the nucleus [55], based on protein sequence identity and phylogenetic analysis. OsDOF2 shares 74.08% nucleotide and 77.14% amino acid sequence identity with EcDOF2 (Figure S6), indicating strong evolutionary conservation between the two grass species.

**FIGURE 2:**
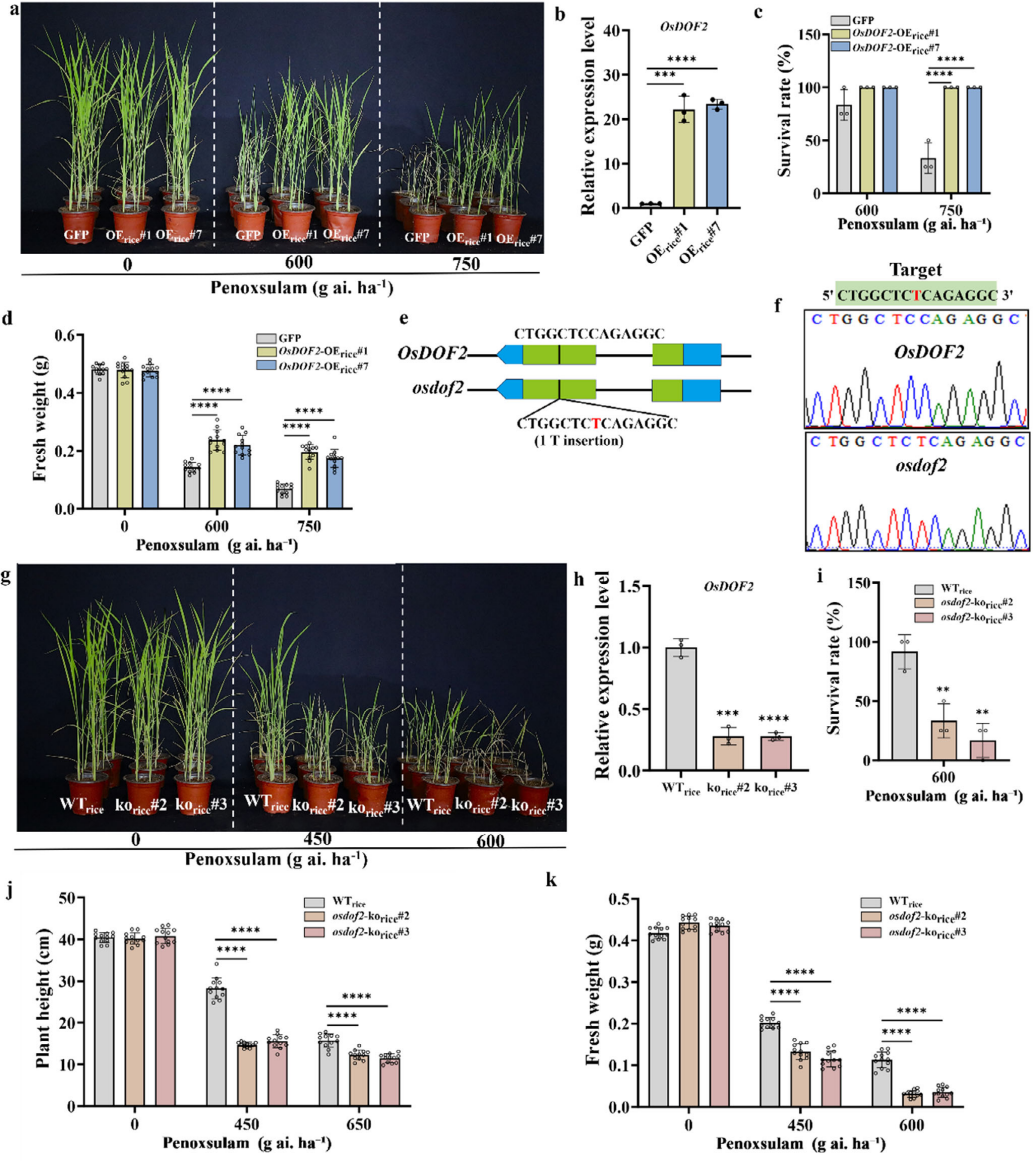
Functional characterization of *OsDOF2* in rice via overexpression and CRISPR/Cas9‐mediated knockout in response to penoxsulam treatment. (a) Phenotypic comparison of *OsDOF2* (OE_rice_#1, OE_rice_#2) and GFP control plants 21 days after penoxsulam treatment. (b) RT‐qPCR quantification of *OsDOF2* expression levels in GFP and OE_rice_ lines. (c) Survival rate and (d) fresh weight of GFP and OE_rice_ plants after penoxsulam treatment in (a) (*n* = 12). (e) Schematic representation of the *OsDOF2* gene structure and the CRISPR/Cas9‐induced *osdof2* mutation. (f) Sanger sequencing chromatograms confirming the mutation site in *osdof2* mutants. (g) Phenotypes of WT_rice_ and *OsDOF2* knockout (*osdof2*‐ko) rice lines (ko_rice_#2 and ko_rice_#3) 21 days after penoxsulam application. (h) RT‐qPCR quantification of *OsDOF2* expression levels in *osdof2*‐ko_rice_ lines relative to WT_rice_ (*n* = 3). (i) Survival rate, (j) plant height, and (k) fresh weight of WT_rice_ and *osdof2*‐ko_rice_ plants after penoxsulam treatment in (g) (*n* = 12 plants). Data are mean ± SD, statistical significance was assessed using two‐tailed Student's t‐test for (b, h, i), (**p < 0.01, ***p < 0.001, ****p < 0.0001); Two‐way ANOVA followed by Dunnett's multiple comparison test was performed for (c, d, j, k).

To assess the role of *OsDOF2* in herbicide tolerance, we generated transgenic rice lines overexpressing *OsDOF2* under the control of the CaMV 35S promoter. *OsDOF2*‐overexpression (*OsDOF2*‐OE_rice_) rice lines (OE_rice_#1 and OE_rice_#7) displayed significantly enhanced tolerance to GFP controls, with improved growth performance under 600 and 750 g ai. ha^−^^1^ herbicide treatment (Figure 2a). RT‐qPCR confirmed *OsDOF2* overexpression in these lines (Figure 2b), which correlated with the higher survival rate (Figure 2c) and greater fresh weight than GFP controls (Figure 2d).

To further validate the function of *OsDOF2* in herbicide tolerance, *osdof2* loss‐of‐function mutants were generated using CRISPR/Cas9, introducing a single thymine insertion that caused a frameshift mutation (Figure 2e,f). Two homozygous *osdof2* knockout mutant line (*osdof*2‐ko_rice_#2, *osdof2*‐ko_rice_#3) was confirmed by DNA sequencing and selected for phenotypic analysis. Compared with WT_rice_, *osdof2‐*ko_rice_ mutants exhibited marked sensitivity to penoxsulam. The *osdof2‐*ko_rice_ *#2* and *osdof2‐*ko_rice_ *#3* lines showed stunted growth at 450 and 600 g ai. ha^−^^1^ penoxsulam rates (Figure 2g). RT‐qPCR analysis confirmed that *OsDOF2* expression was significantly reduced in the knockout lines (Figure 2h). Under penoxsulam treatment, *osdof2*‐ko_rice_ lines exhibited significantly lower survival rates, reduced plant height, and decreased fresh weight compared with WT_rice_ (Figure 2i–k). Together, these gain‐ and loss‐of‐function studies establish *OsDOF2* as a positive regulator of penoxsulam tolerance in rice. Comparable phenotypic response of *EcDOF2* and *OsDOF2* genotypes to penoxsulam suggest conserved DOF transcription regulation of metabolic herbicide resistance/tolerance in weedy and crop grass species.

Overall, *EcDOF2* and its ortholog *OsDOF2* positively regulate penoxsulam resistance/tolerance in *E. crus‐galli* and rice, respectively, as supported by complementary overexpression and knockout analyses. The conserved function of *DOF2* across species suggests that DOF‐mediated transcriptional regulation represents a shared mechanism controlling herbicide stress adaptation in grasses.

### 2.3 Genome‐Wide Identification of *EcDOF2* Binding Sites Using DAP‐seq

DNA affinity purification sequencing (DAP‐seq) has recently emerged as a powerful tool to capture in vitro TF‐DNA interactions across the genome within native genomic contexts [56, 57]. To elucidate the transcriptional regulatory mechanisms underlying EcDOF2‐mediated penoxsulam resistance, we performed DAP‐seq to identify genome‐wide binding sites of EcDOF2, and recombinant *EcDOF2* fused with a HaloTag was used to purify genomic DNA from *E. crus‐galli*. Two independent biological replicates were prepared, including matched “Input” libraries as negative controls, and subjected to high‐throughput sequencing. The number of total clean reads and mapping rates are summarized in Table S4. A total of 63, 036 high‐confidence binding peaks were consistently identified across the two replicates (Figure S7a). Among these, 12, 733 peaks were located in promoter regions, corresponding to 17, 311 putative target genes, including 53 P450 genes. The binding regions ranged from 240 to 1500 bp in length, and peak distribution was highly enriched around transcription start sites (TSSs) (Figure S7b). Genomic annotation of the binding peaks showed that *EcDOF2* predominantly binds to distal intergenic regions (58.1%), followed by introns (18.2%), promoters within 1 kb (11.3%) and 1–2 kb (8.9%), exons (2.4%), and downstream regions (1.1%) (Figure S7c). Notably, 20.2% of the peaks were located within 2 kb upstream of TSSs, which is consistent with its function as a DNA‐binding transcription factor. Furthermore, peak distribution across chromosomes correlated with chromosome length (Figure S7d), suggesting no chromosomal bias in EcDOF2 binding.

To further characterize the DNA‐binding properties of *EcDOF2*, de novo motif discovery using MEME‐ChIP identified two significantly enriched DNA motifs among EcDOF2 binding sites. The most prevalent motif, AAAAAGGTAAAAAGT (E‐value = 4.5e–1975), was detected in 3342 peaks, while a secondary motif, AAGTGAAAAAGTCACATCGGA (E‐value = 6.3e–2296), occurred in 864 peaks (Figure S7e). Notably, both motifs contain the conserved (A/T) AAAG core sequence, which is a previously characterized binding motif for DOF transcription factors [58], thereby validating the specificity and reliability of our DAP‐seq results. The flanking nucleotides likely contribute to binding specificity and modulate the binding affinity of *EcDOF2*.

### 2.4 Identification of DOF2‐Regulated Genes in *E. crus‐galli* and Rice by RNA‐seq

To identify genes transcriptionally regulated by EcDOF2, we conducted RNA‐seq analysis using the 3–4 leaf stage seedlings from the two *E. crus‐galli EcDOF2*‐OE_EC_ lines (OE_EC_#5 and OE_EC_#7) and WT_EC_ plants. After quality filtering, a total of 188 936 073 clean reads were obtained, with mapping rates ranging from 91.58% to 95.13% across samples (Table S5).

**FIGURE 3:**
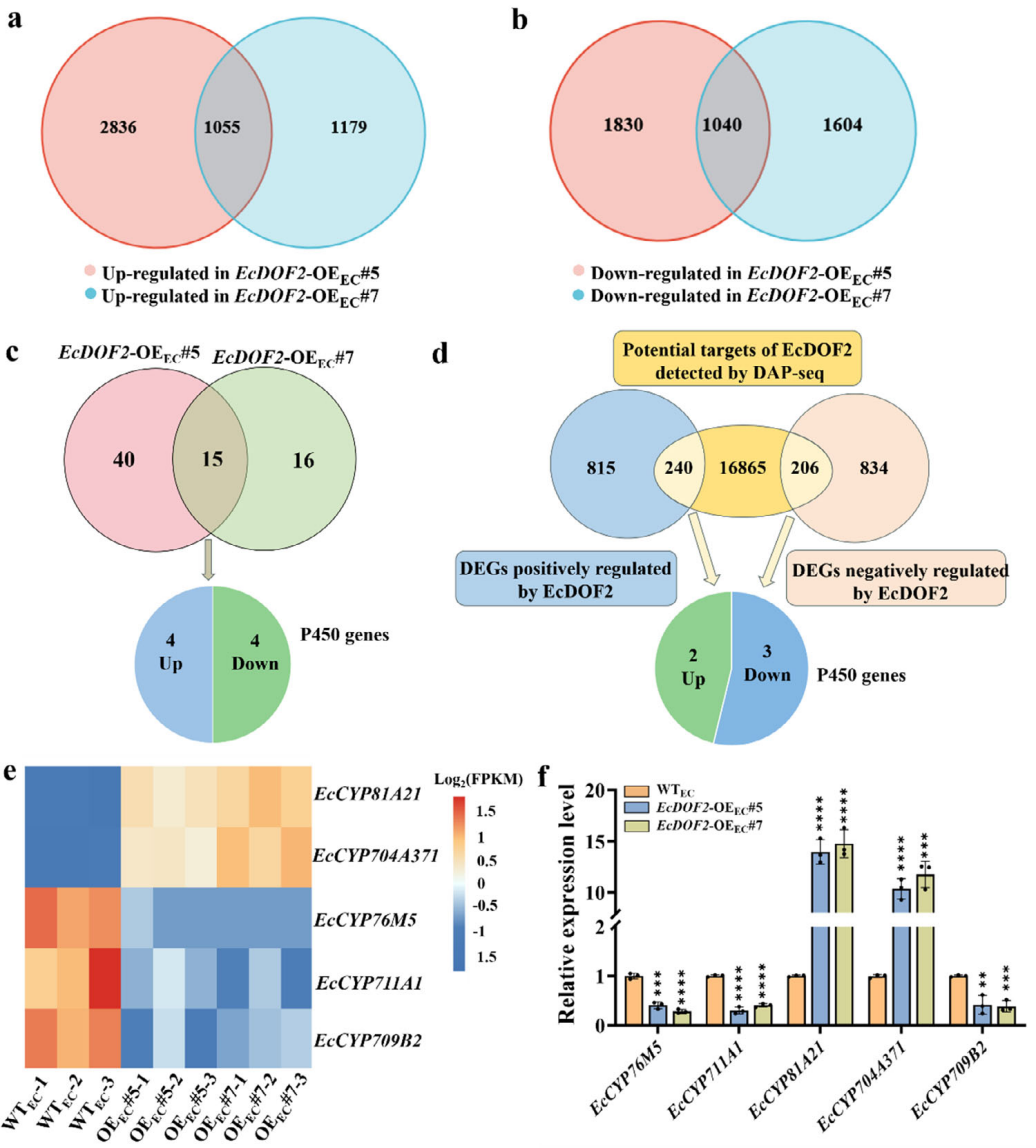
Identification of EcDOF2 direct target genes involved in herbicide detoxification in *Echinochloa crus‐galli*. (a, b) Venn diagrams displaying the overlap of upregulated (a) and downregulated (b) DEGs between the two OE lines (*EcDOF2*#5and *EcDOF2*#7). (c) Venn diagram showing the overlap of differentially expressed P450 genes between *EcDOF2*‐OE_EC_#5 and *EcDOF2*‐OE_EC_#7 lines compared with WT_EC_ plants. (d) Venn diagram showing the overlap between EcDOF2‐bound genes identified by DAP‐seq and DEGs identified by RNA‐seq from *EcDOF2*‐OE_EC_ *E. crus‐galli* lines. The numbers of directly upregulated and downregulated target genes were indicated. (e) Heatmap of RNA‐seq expression for seven cytochrome P450 genes predicted as direct EcDOF2 targets in WT and *EcDOF2*‐OE_EC_ lines. (f) RT‐qPCR validation of selected P450 genes in WT_EC_ and *EcDOF2*‐OE_EC_ lines (OE_EC_#5 and OE_EC_#7). Data are mean ± SD (*n* = 3).

Differential expression analysis revealed 6761 DEGs in the *EcDOF2*‐OE_EC_ #5 line compared to WT_EC_, including 2870 downregulated (Log_2_ FC < −1, FDR < 0.05) and 3891 upregulated (Log_2_ FC > 1, FDR < 0.05) genes (Figure S8a). Similarly, 4878 DEGs were identified in OE_EC_#7, comprising 2234 upregulated and 2644 downregulated genes (Figure S8b). Comparative analysis revealed 1055 commonly upregulated and 1040 commonly downregulated genes between the two overexpression lines (Figure 3a,b). Consistent with the P450 inhibitor assays indicating that P450‐mediated metabolism contributes to penoxsulam resistance in *E. crus‐galli* (Table S1), transcriptome profiling of *EcDOF2*‐OE_EC_ lines revealed significant transcriptional changes in multiple cytochrome P450 genes. Differential expression analysis identified 55 and 31 P450 genes in OE_EC_#5 and OE_EC_#7 lines, respectively, with 15 genes shared between the two lines (Figure 3c). Among these, 4 genes were consistently upregulated. Heatmap clustering further showed that these shared genes displayed consistent expression patterns between transgenic and WT_EC_ plants (Figure S8c). Notably, the P450 gene *EcCYP81A21*, was significantly upregulated in the two OE_EC_ lines. These findings are consistent with our hypothesis that EcDOF2 regulates herbicide detoxification pathways via transcriptional regulation of certain P450 genes.

We have already showed that the rice ortholog *OsDOF2* gene confers enhanced penoxsulam tolerance in rice (Figure 2a). To investigate how OsDOF2 regulates this process, we performed RNA‐seq analysis comparing rice WT_rice_ and the *osdof2*‐ko_rice_ plants. A total of 176 855 274 clean reads were obtained from six cDNA libraries (three biological replicates per genotype), with Q20 values >99.12% and GC content >47.24% (Table S6). Comparative analysis identified 4499 DEGs between *osdof2*‐ko_rice_ and WT_rice_ plants, including 1457 upregulated genes and 3042 downregulated genes (Figure S9a). The majority of DEGs were downregulated in *osdof2*‐ko_rice_, consistent with a predominant role for OsDOF2 as a transcriptional activator. KEGG enrichment analysis revealed significant representation in multiple metabolic and signaling pathways (Figure S9b). Pathways related to environmental adaptation and xenobiotic metabolism–often linked to herbicide detoxification–were enriched, suggesting a broad role of *OsDOF2* in regulating metabolic networks under stress conditions. A total of 46 P450 genes were identified as differentially expressed, with 41 showing significant downregulation (Figure S9c).

Together, RNA‐seq analyses demonstrate that DOF2 functions predominantly as a transcriptional activator in both *E. crus‐galli* and rice, regulating genes involved in stress responses and xenobiotic metabolism. Notably, multiple P450 genes, including the resistance‐associated *EcCYP81A21*, are upregulated in *EcDOF2*‐OE_Ec_ lines, linking DOF2 activity to herbicide detoxification pathways. These findings indicate a conserved transcriptional framework by which DOF2 modulates herbicide tolerance across grass species.

### 2.5 Determination of Potential DOF2‐Regulated Target P450 Genes in *E. crus‐galli* and Rice

To identify potential direct targets regulated by EcDOF2, we performed an integrative analysis combining DAP‐seq and RNA‐seq data that were described above. A total of 17, 311 EcDOF2‐bound genes were identified by DAP‐seq, and 2, 095 DEGs detected in *EcDOF2‐OE*_EC_ *E. crus‐galli* plants by RNA‐seq. Cross‐referencing these datasets revealed 446 overlapping genes (Figure 3d), suggesting that these are likely direct targets of EcDOF2. Among them, 240 genes (53.8%) were upregulated, indicating a possible role of EcDOF2 as a transcriptional activator. In contrast, 206 genes (46.2%) were downregulated, implying that EcDOF2 may also function as a repressor for certain target genes.

GO enrichment analysis of the 446 direct targets showed significant overrepresentation of functional categories associated with detoxification and stress adaptation (Figure S10a). These categories are closely related to redox metabolism and detoxification processes, suggesting that EcDOF2 may regulate genes involved in detoxification pathways, such as those encoding P450s, thereby contributing to herbicide resistance. KEGG pathway enrichment further supported this, highlighting pathways such as plant hormone signal transduction (Figure S10b), linking EcDOF2 to both stress response and metabolic adaptation.

Among these targets, we identified five P450 genes with potential roles in herbicide metabolism (Figure 3e). Consistently, the transcript levels of *EcCYP81A21* (13.9‐ and 14.7‐fold in OE_EC_#5 and OE_EC_#7) and *EcCYP704A371* were higher (10.3‐ and 11.7‐fold in OE_EC_#5 and OE_EC_#7), whereas *EcCYP76M5*, *EcCYP71A1* and *EcCYP709B2* were lower, in *EcDOF2*‐OE_EC_ lines than in WT_EC_ (Figure 3f) As EcCYP81A21 shares 99.24% amino acid sequence identity with the known EpCYP81A21 in *E. phyllopogon* [9], they likely function through similar metabolic mechanisms and confer resistance to penoxsulam (Figure S11a). These results strongly suggest that EcDOF2 directly activates detoxification‐related P450 genes, thereby contributing to metabolic herbicide resistance in *E. crus‐galli*.

Focusing on P450 genes in rice, a well‐known major herbicide detoxification P450, OsCYP81A6^13^, sharing 82.64% sequence identity with EcCYP81A21 (Figure S11b), exhibited the most pronounced decrease in transcript abundance in the *DOF2* knockout lines (Figure S9c). Another P450 genes, OsCYP704C1, sharing 73.74% amino acid identity with EcCYP704A371 (Figure S11c), was also markedly downregulated (Figure S9c). However, RT‐qPCR analysis confirmed significantly reduced transcript levels of *OsCYP81A6* but not *OsCYP704C1* in the *osdof2*‐ko_rice_ compared to WT_rice_ plants (Figure S12a,b). These results indicate that like EcDOF2, OsDOF2 positively upregulates certain P450 genes associated with herbicide detoxification, and loss of function disrupts this transcriptional network, leading to increased penoxsulam susceptibility (Figure 2g).

In summary, integrative DAP‐seq and RNA‐seq analyses identify a set of direct DOF2 targets enriched in detoxification‐ and stress‐related pathways. Notably, certain cytochrome P450 genes, including *EcCYP81A21* and its rice ortholog *OsCYP81A6*, are positively regulated by DOF2. Loss of DOF2 function disrupts this DOF‐centered transcriptional network, providing a mechanistic basis for enhanced penoxsulam susceptibility.

### 2.6 DOF2 Directly Bind to the Promoters of P450s for Transcription Activation in *E. crus‐galli* and Rice

**FIGURE 4:**
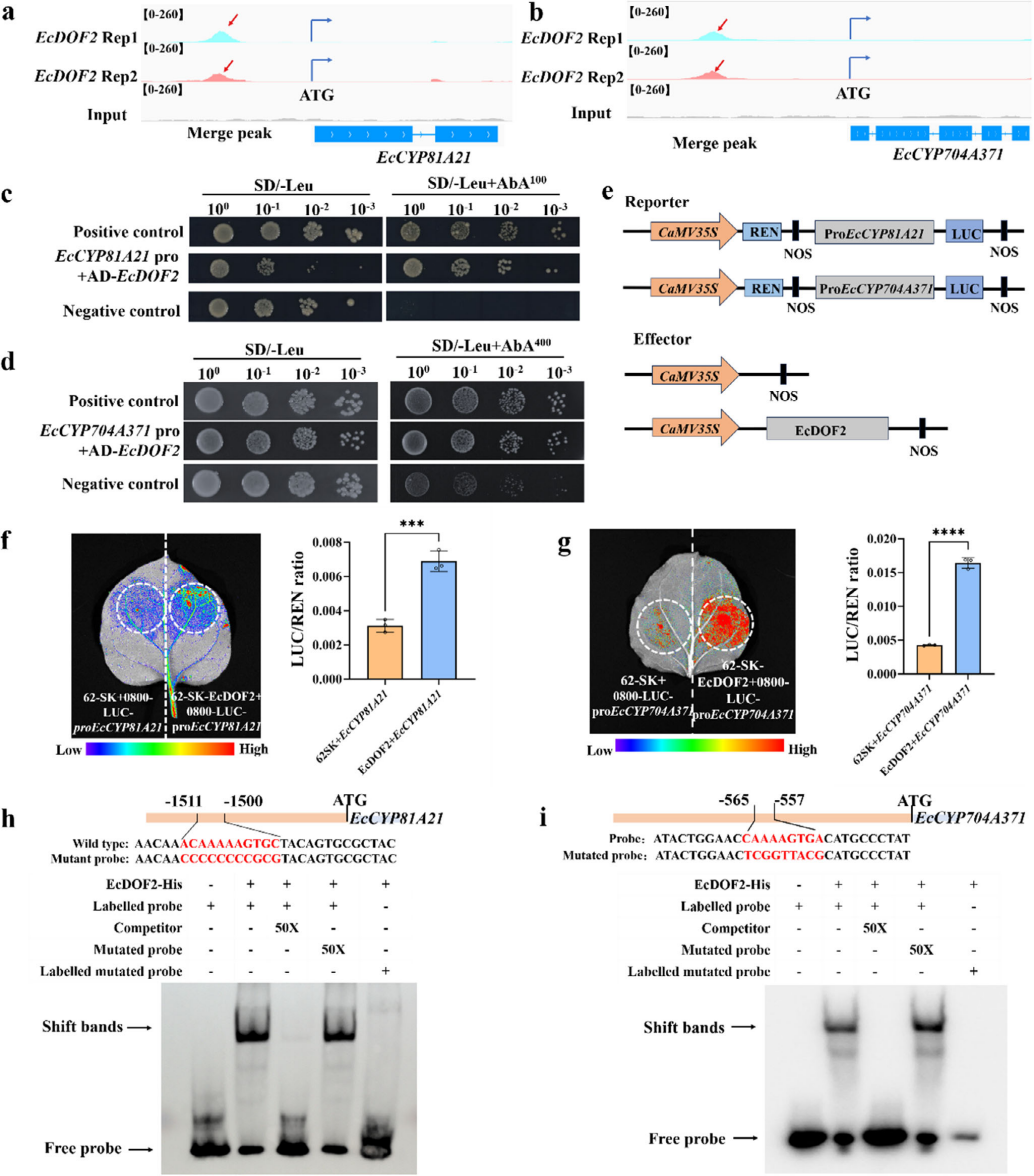
Direct binding of EcDOF2 to the promoters of *E. crus‐galli EcCYP81A21* and *EcCYP704A371* for transcription activation. (a, b) DAP‐seq genome browser views showing EcDOF2 binding peaks upstream of *EcCYP81A21* (a) and *EcCYP704A371* (b). (c, d) Y1H assays demonstrating EcDOF2 binding to the promoters of *EcCYP81A21* (c) and *EcCYP704A371* (d). (e) Schematic diagrams of effector and reporter constructs used in dual‐luciferase assays. f, g. Firefly luciferase imaging and signal quantification showing activation of *EcCYP81A21* (f) and *EcCYP704A371* (g) promoters by EcDOF2 in *Nicotiana benthamiana* leaves. (h, i) EMSA confirming in vitro binding of EcDOF2 to the *EcCYP81A21* (h) and *EcCYP704A371* (i) promoter fragments. Biotin‐labeled probes containing EcDOF2‐binding motifs were used; competition and mutation controls confirm specificity. Bar plots data are means ± SD (*n* = 3). Asterisks denote significance by two‐tailed Student's t‐test (***p < 0.001, ****p < 0.0001).

In *E.crus‐galli*, to determine whether EcDOF2 directly regulates *EcCYP81A21* and *EcCYP704A371*, we first analyzed their promoter regions using DAP‐seq. Distinct EcDOF2 binding peaks were identified upstream of both genes (Figure 4a,b), containing a conserved DOF‐binding motif (AAGTGAAAAAGTCACATCGGA). To confirm the DAP‐seq results, Y1H assays were conducted and results further demonstrated that EcDOF2 interacts specifically with the promoter fragments of *EcCYP81A21* and *EcCYP704A371*. Positive yeast colonies expressing AD‐EcDOF2 and pAbAi‐*EcCYP81A21*/*EcCYP704A371* promoter constructs showed normal growth on the selective SD‐Leu + AbA medium, confirming specific protein–DNA interaction (Figure 4c,d). Additionally, dual‐luciferase transient expression assays in *N. benthamiana* leaves demonstrated that EcDOF2 significantly enhanced the activity of the *EcCYP81A21* and *EcCYP704A371* promoters, with LUC/REN ratios markedly higher than vector controls (Figure 4e–g). These results provide evidence that EcDOF2 acts as a transcriptional activator of these P450 genes. Furthermore, we performed EMSA using purified recombinant EcDOF2‐His protein to validate the interaction between EcDOF2 and the promoter fragments of *EcCYP81A21* and *EcCYP704A371*. A distinct mobility shift was observed when biotin‐labeled DNA probes containing the DAP‐seq identified binding sites were incubated with the EcDOF2 protein (Figure 4h,i), indicating direct binding of EcDOF2 to these promoter regions. The addition of unlabeled competitor probes carrying the intact binding motifs effectively abolished the shift, whereas probes with mutated motifs failed to do so (Figure 4h,i). All these in vivo and in vitro results demonstrated that EcDOF2 specifically binds to the promoters of *EcCYP81A21* and *EcCYP704A371*.

In rice, promoter analysis identified multiple putative cis‐elements in the *OsCYP81A6* promoter, including the conserved DOF‐binding motifs. Y1H assays demonstrated that yeast cells co‐transformed with *OsCYP81A6* promoter‐pAbAi and *OsDOF2*‐pGADT7 constructs could grow on the selective medium containing AbA (Figure S12c), indicating that OsDOF2 binds to the *OsCYP81A6* promoter in vivo. Dual‐luciferase assays in *N. benthamiana* leaves further demonstrated that co‐expression of *OsDOF2* significantly enhanced LUC activity driven by the *OsCYP81A6* promoter, compared to controls (Figure S12d,e), confirming that OsDOF2 functions as a transcriptional activator. EMSA validated this interaction in vitro: the OsDOF2‐His fusion protein specifically bound to a biotin‐labeled probe containing the TGAAAAGGTG motif, and this interaction was abolished by competition with excess unlabeled but not mutated probes (Figure S12f). Consistent with RT‐qPCR results (Figure S12a,b), Y1H and dual‐luciferase assays showed no detectable interaction between OsDOF2 and the *OsCYP704C1* promoter, suggesting that OsDOF2 may not directly regulate *OsCYP704C1* transcription through promoter binding (Figure S12g,h). Together, these results demonstrated that OsDOF2 directly binds to the *OsCYP81A6* promoter for transcription activation.

Together, in vivo and in vitro assays demonstrate that DOF2 directly binds to the promoters of the key detoxification‐related P450 genes and activates their transcription. EcDOF2 specifically interacts with the promoters of *EcCYP81A21* and *EcCYP704A371* in *E. crus‐galli*, while OsDOF2 directly targets the *OsCYP81A6* promoter in rice. These findings establish a conserved DOF2–P450 regulatory axis underlying herbicide detoxification.

### 2.7 Expression of *DOF2* Downstream P450s Confers Herbicides Resistance/Tolerance in *E. crus‐galli* and Rice

**FIGURE 5:**
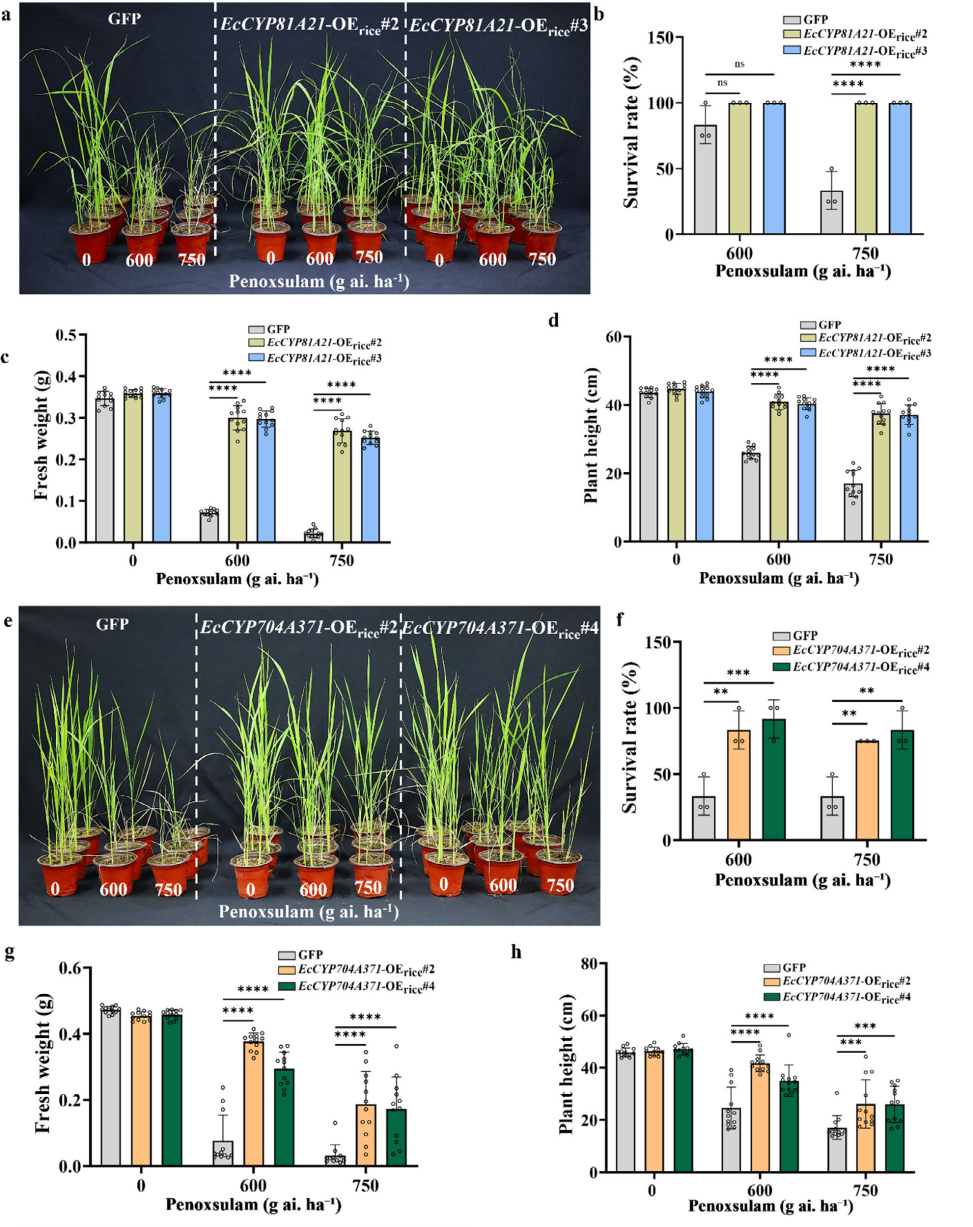
Overexpression of *EcDOF2* downstream target genes (*EcCYP81A21* and *EcCYP704A371*) enhance penoxsulam tolerance in rice. (a) Phenotypic responses of *EcCYP81A21*‐OE_rice_ (lines #2 and #3) and GFP control plants to penoxsulam treatment (0, 600, and 750 g ai. ha^−^^1^). Photo was taken 21 days after treatment. (b–d) Quantification of survival rate (b), fresh weight (c), and plant height (d) of GFP and *EcCYP81A21*‐OE_rice_ lines following penoxsulam treatment in (a). (e) Phenotyic response of *EcCYP704A371*‐OE_rice_ (lines #2 and #4) and GFP control plants to penoxsualm treatment. Photo was taken 21 days after treatment. (f–h) survival rate (f), fresh weight (g), and plant height (h) of GFP and *EcCYP704A371*‐OE_rice_ lines following penoxsulam treatment in (e). Data are means ± SD, Two‐way ANOVA followed by Dunnett's multiple comparison test was performed for (b, c, d, f, g, h). Asterisks indicate significant differences compared with the GFP control (**p < 0.01, ***p < 0.001, ****p < 0.0001).

To investigate the functional relevance of EcDOF2 downstream targets in metabolic herbicide resistance, we generated transgenic rice lines overexpressing *EcCYP81A21* (OE_rice_#2 and OE_rice_#3) and *EcCYP704A371* (OE_rice_#2 and OE_rice_#4). For *EcCYP81A21*, overexpression enhanced rice tolerance to penoxsulam. When treated with penoxsulam (at 600 and 750 g ai. ha^−^^1^), *EcCYP81A21* transgenic lines exhibited markedly higher survival rate, fresh weight, and plant height compared to GFP controls (Figure 5a–d). Similarly, overexpression of *EcCYP704A371* improved rice tolerance to penoxsulam. *EcCYP704A371* transgenic plants showed higher biomass accumulation and survival rate under the same herbicide rates (Figure 5e–h). Together, these findings established *EcCYP81A21* and *EcCYP704A371* as direct transcriptional targets of EcDOF2 and functional effectors in metabolic herbicide resistance. EcDOF2 thereby serves as a key transcriptional activator linking upstream regulatory signals to downstream detoxification capacity in *E. crus‐galli*.

As *EpCYP81A21* (*CYP81A21* from *E. phyllopogon*) is highly homologous to *EcCYP81A21* and is known to confer resistance to the ACCase‐inhibiting herbicide pinoxaden and bensulfuron‐methyl [9], we also compared the response of WT_EC_ and *EcDOF2*‐OE_EC_ *E. curs‐galli* plants (line #5 and #7) to pinoxaden and bensulfuron‐methyl (Figure S13a–f). WT_EC_ plants exhibited clear growth inhibition and even death under higher rates of both herbicides, whereas the transgenic lines maintained relatively better growth (Figure S13a,d). *EcDOF2*‐OE_EC_ plants displayed significantly greater plant height and fresh weight than WT_EC_ under herbicide stress (Figure S13b,c,e,f). These results demonstrated that overexpression of *EcDOF2* confers enhanced resistance of *E. crus‐galli* to both pinoxaden and bensulfuron‐methyl, in addition to penoxsulam.

*OsCYP81A6* is known to confer tolerance to ALS‐inhibiting sulfonylurea herbicides [13], to functionally validate if *OsCYP81A6* is involved in penoxsulam tolerance, we generated *OsCYP81A6* overexpressing (*OsCYP81A6*‐OE_rice_) transgenic rice lines. Two independent homozygous lines (OE_rice_#1 and OE_rice_#3) were selected for phenotypic assays. When treated with penoxsulam at 600 or 750 g ai. ha^−^^1^, *OsCYP81A6*‐OE_rice_ plants (OE_rice_#1 and OE_rice_#3) exhibited visibly enhanced tolerance, greater biomass, and a higher survival rate than GFP controls (Figure S14a–d). These results demonstrated that *OsCYP81A6* overexpression endows increased penoxsulam tolerance, supporting its role as a key downstream effector of the OsDOF2 regulatory network in rice.

Collectively, these gain‐of‐function analyses confirmed that DOF2 downstream P450s, including *EcCYP81A21*, *EcCYP704A371*, and *OsCYP81A6*, are sufficient to endow herbicide resistance in *E. crus‐galli* and enhance herbicide tolerance in rice. These findings establish a DOF2–P450 regulatory cascade linking transcriptional control to detoxification capacity in both weedy and crop grasses.

### 2.8 GAs Signaling is Likely the Upstream Regulator of EcDOF2 for Penoxsulam Resistance in *E. crus‐galli*

**FIGURE 6:**
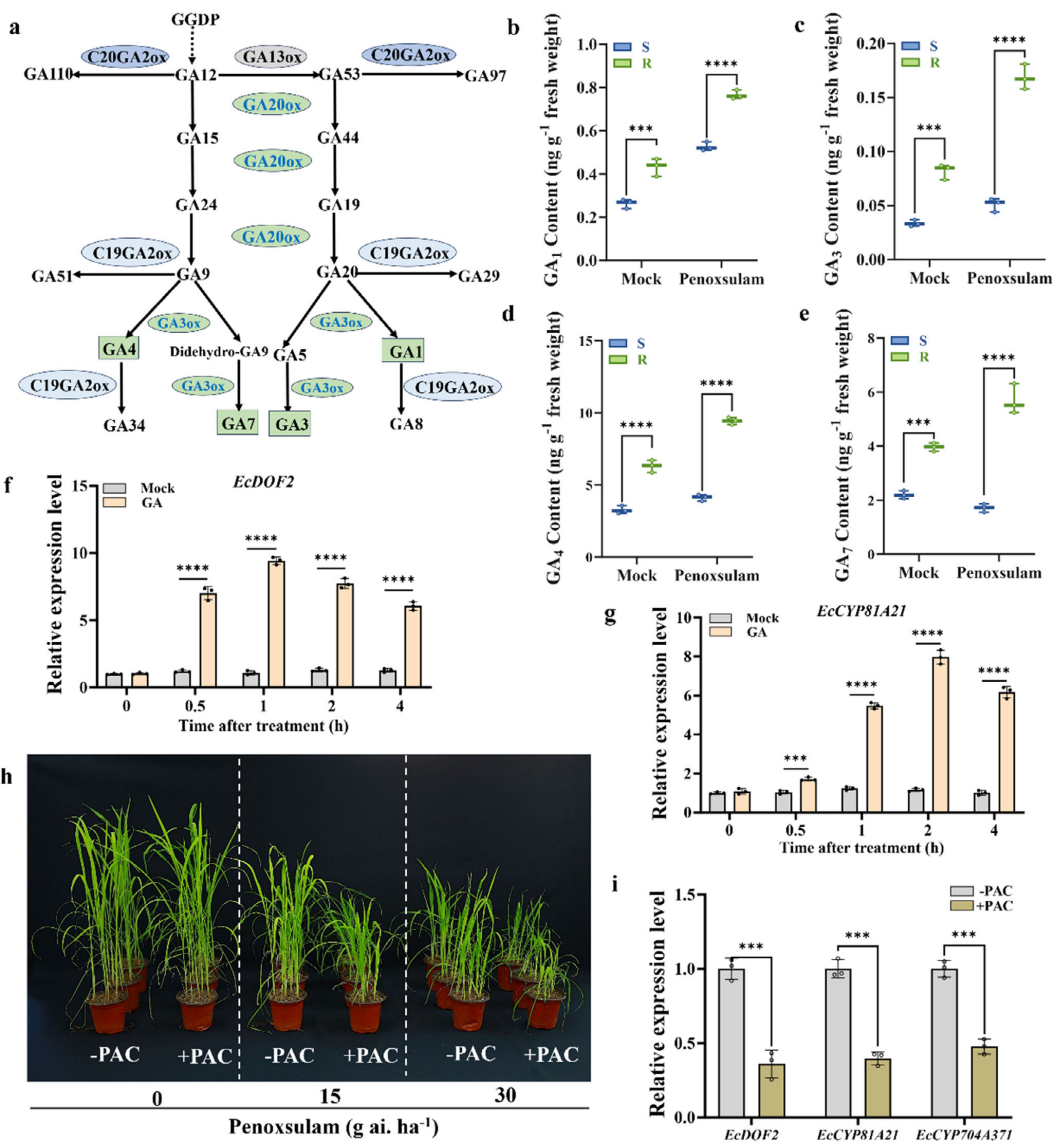
Gibberellin positively regulates *EcDOF2* expression, leading penoxsulam resistance in *Echinochloa crus‐galli*. (a) Simplified GA biosynthesis pathway showing key GA biosynthetic enzymes (blue) and major bioactive GAs (green squares). (b–e) Endogenous levels of bioactive GAs in R and S plants under mock or penoxsulam treatment, including (b) GA_1_, (c) GA_3_, (d) GA_4_, and (e) GA_7_. (f–g) Time‐course expression analysis of *EcDOF2*, and *EcCYP81A21* in *E. crus‐galli* leaves following GA_3_ treatment (20 µM). (h) Phenotypic response of R plants to penoxsulam with and without PAC treatment. The photo was taken 21 days after treatment. (i) Relative expression levels of *EcDOF2*, *EcCYP81A21*, and *EcCYP704A371* in R plants following PAC treatment. Data are mean ± SD. Statistical significance was determined using two‐tailed Student's t‐test for (i); Two‐way ANOVA followed by Sidak's multiple comparison test was performed for (b, c, d, e, f, g). Asterisks indicate significant differences (***p < 0.001, ****p < 0.0001).

GAs is known to regulate plant growth and environmental adaptation, and DOFs integrate GA signals to modulate plant stress responses [43, 59]. To explore whether GA signaling is involved in herbicide metabolic resistance in *E. crus‐galli*, we first analyzed genes associated with GA metabolism and perception from RNA‐seq data, as illustrated in the GA biosynthetic pathway schematic (Figure 6a). RT‐qPCR analyses revealed that the GA biosynthetic genes *EcGA20ox*, and *EcGA3ox* were significantly (*p *< 0.0001) upregulated in the R *E. crus‐galli* compared with the S plants (Figure S15a). Consistent with the transcript pattern, the levels of bioactive GA_1,_ GA_3,_ GA_4_, and GA_7_ were significantly higher in R than in S plants under both mock and penoxsulam treatment (Figure 6b–e).

To examine whether GA signaling regulates *EcDOF2* and its downstream detoxification pathways, exogenous GA_3_ was applied. GA treatment rapidly induced *EcDOF2* expression within 30 min, the expression reached the highest level 1 h after treatment (Figure 6f). Similarly, the downstream detoxification genes *EcCYP81A21* and *EcCYP704A371* were significantly upregulated 1 h post‐treatment (Figure 6g and Figure S15b), suggesting a temporal cascade in which GA activates *EcDOF2*, and which then activates P450 gene expression. To functionally assess GA involvement in resistance, paclobutrazol (PAC), a GA biosynthesis inhibitor [60], significantly inhibits GA synthesis (Figure S15c–f), was applied together with penoxsulam. Phenotypic assays showed that PAC co‐application lowered the survival rate and inhibited growth of R *E. curs‐galli* plants (Figure 6h), with significant decreases in fresh weight (Supplemental Figure S15g) and plant height (Figure S15h) compared with penoxsulam treatment alone. At the transcription level, PAC treatment suppressed the expression of *EcDOF2*, *EcCYP81A21*, and *EcCYP704A371* (Figure 6i), suggesting that GA signaling is likely the upstream regulator of DOF and P450s. Moreover, we examined the effect of exogenous GA_3_ on the S population. Under penoxsulam treatment alone, susceptible plants exhibited pronounced growth inhibition in a dose‐dependent manner, characterized by reduced plant height and fresh weight (Figure S15i). In contrast, co‐application of GA_3_ markedly alleviated penoxsulam‐induced growth suppression. GA_3_‐treated plants displayed significantly greater plant height and fresh weight compared with mock controls, particularly at higher herbicide rates (7.5 g ai. ha^−^^1^) (Figure S15j,k). To determine whether GA_3_‐induced herbicide tolerance depends on DOF2, we further examined the response of *osdof2* mutant plants to GA_3_ under penoxsulam treatment. Exogenous GA_3_ application failed to improve the fresh weight, or survival rate of *osdof2*‐ko_rice_ plants treatment with penoxsulam (Figure S16a–c). No significant differences were observed between GA_3_‐treated and mock‐treated *osdof2*‐ko_rice_ plants, indicating that DOF2 is indispensable for GA‐mediated enhancement of herbicide tolerance. Meanwhile, GA_3_ treatment did not significantly alter the expression of *OsDOF2* or *OsCYP81A6* in *osdof2*‐ko rice plants (Figure S16d). These results indicate that GA_3_‐induced expression of *OsCYP81A6* is dependent on the presence of functional *OsDOF2*.

Together, these results imply that GA signaling enhances penoxsulam resistance in *E. crus‐galli* and penoxsulam tolerance in rice by activating *DOF2*, which in turn transcriptionally activates P450 detoxification genes.

### 2.9 GID1 Plays a Positive Role in DOF2‐Dependent Herbicide Resistance in Rice and *E. crus‐galli*

**FIGURE 7:**
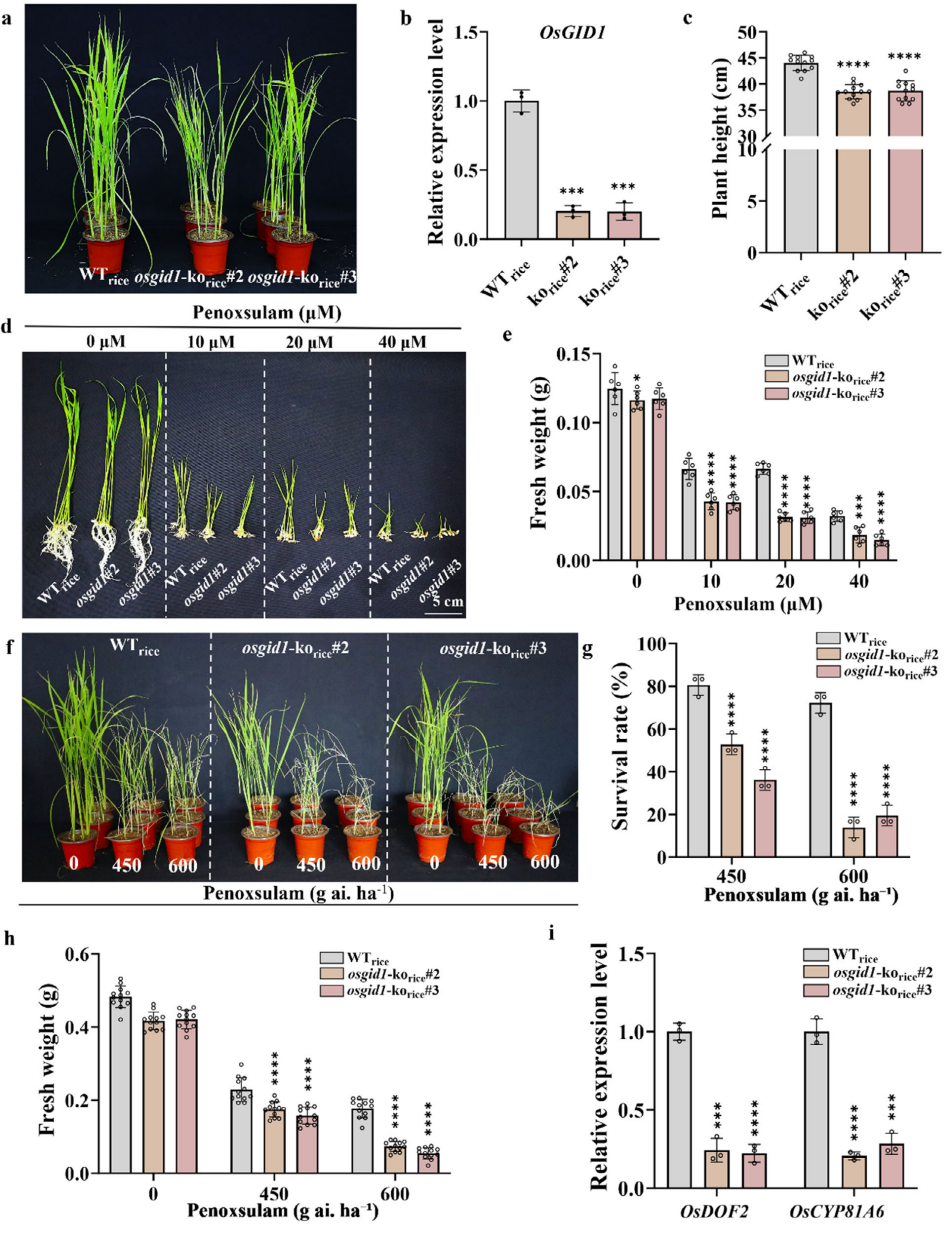
The GA receptor *OsGID1* regulates rice tolerance to penoxsulam. (a) Phenotypic comparison of WT_rice_ and *osgid1* knockout lines (*osgid1*‐ko_rice_#2 and *osgid1*‐ko_rice_#3) grown under normal conditions. (b) RT‐qPCR quantification of *OsGID1* expression in the mutants versus WT_rice_. (c) Plant height of WT_rice_ and mutants grown under normal conditions for 21 days. (d) Phenotypic and (e) growth (fresh weight) response of WT_rice_ and mutant seedlings to penoxsulam treatment, and data were recorded 10 days after treatment. (f) Phenotypes of WT_rice_ and mutants treated with penoxsulam. Photo was taken 21 days after treatment. (g, h) Survival rate (g) and fresh weight (h) of WT_rice_ and mutants following penoxsulam treatments in (f). (i) RT‐qPCR quantification of *OsDOF2* and *OsCYP81A6* expression in the mutants versus WT_rice_ under untreated conditions. Data are mean ± SD. Two‐tailed Student's t‐test for (b, c, i), Two‐way ANOVA followed by Dunnett's multiple comparison test was performed for (e, g, h). Asterisks indicate statistically significant differences from WT_rice_ (*p < 0.05, ***p < 0.001, ****p < 0.0001).

*OsGID1* serves as a key receptor mediating GA signaling in plants [41]. In rice, knockout of *OsGID1* disrupts GA signal transduction [46]. RT‐qPCR analyses revealed that the GA receptor gene *EcGID1* were significantly (*p*<0.0001) upregulated in the R *E. crus‐galli* compared with the S plants (Figure S15a). Hence, we hypothesize *GID1* may play a role in herbicide resistance/tolerance by regulating GA signaling. To test this, we generated rice *OsGID1* knockout mutants (*osgid1*‐ko_rice_). *OsGID1* comprises two exons and one intron, and a single base (A) insertion in the coding region introduced a frameshift mutation (Figure S17a,b). The knockout mutants exhibited a typical GA‐deficient phenotype, characterized by altered plant height compared with WT_rice_ (Figure 7a). RT‐qPCR analysis confirmed that *OsGID1* transcript abundance was markedly reduced in the mutants (Figure 7b). Consequently, plant height was significantly decreased in the two *osgid1*‐ko_rice_ lines relative to WT_rice_ (Figure 7c), validating impaired GA signaling.

To evaluate whether OsGID1 impacts herbicide tolerance in rice, seedling growth of WT_rice_ and *osgid1*‐ko_rice_ (#2, #3) on agar containing penoxsulam (0‐40 µM) were compared. Ten days after treatment, *osgid1*‐ko_rice_ exhibited much greater growth inhibition than WT_rice_, as reflected by seedling morphology (Figure 7d) and fresh weight, survival rate, and plant height measurements (Figure 7e, Figure S17c,d). Whole plant assays were also conducted to confirm the results. Under penoxsulam treatment (at 450 and 600 g ai. ha^−^^1^), *osgid1* mutants showed enhanced susceptibility compared with WT_rice_ (Figure 7f), as shown by significant reduction in the survival rate (Figure 7g), fresh weight (Figure 7h), and plant height (Figure S17e). Consistently, RT‐qPCR analysis showed that *OsDOF2* and *OsCYP81A6*, were significantly downregulated in *osgid1*‐ko_rice_ compared with WT_rice_ (Figure 7i).

To further determine if GA‐induced herbicide tolerance is dependent on functional *GID1* signaling, we treated *osgid1*‐ko_rice_ with exogenous GA_3_ followed by penoxsulam treatment. GA_3_ treatment failed to alleviate penoxsulam‐induced growth inhibition in *osgid1*‐ko_rice_ plants. No significant differences were observed in fresh weight, or survival rate between GA_3_‐treated and mock *osgid1*‐ko_rice_ plants under penoxsulam treatment (Figure S17f–h), indicating that GA‐induced penoxsulam tolerance requires functional *GID1* signaling. These results imply the cascade event of GA‐*OsGID1‐OsDOF2*–*OsCYP81A6* as a possible transcriptional activation module. Disruption of this pathway compromises detoxification gene expression, thereby increasing herbicide susceptibility. Similar to rice, this module is likely also operating in *E. crus‐galli*.

### 2.10 DELLA Plays a Negative Role in DOF2‐Dependent Herbicide Resistance in Rice and *E. crus‐galli*

Contrary to GID1, DELLA proteins are central repressors of GA signaling and act by inhibiting downstream transcription factors [41]. Given that GA promotes herbicide resistance via DOF2, we hypothesize that DELLA may negatively regulate this pathway. To text this, we examined protein–protein interactions between DELLA and DOF2 in both *E. crus‐galli* and rice. We first identified a DELLA homolog in *E. crus‐galli*, designated as EcDELLA (1878 bp, 626 aa), possessing 91.39% protein sequence similarity to OsDELLA (Figure S18), suggesting potential functional conservation across species.

**FIGURE 8:**
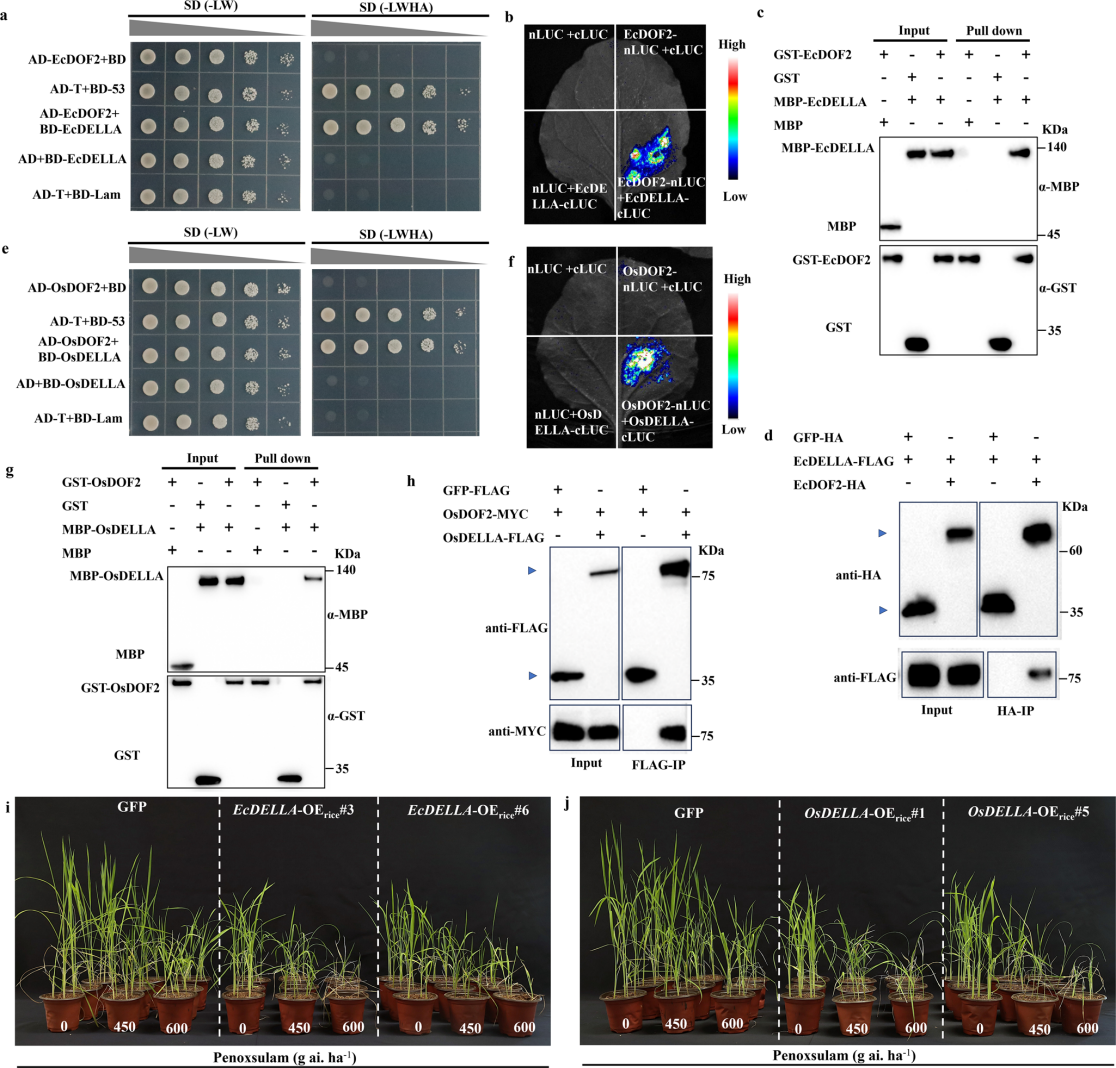
Physical interaction of DELLA proteins with DOF2 and negative regulation of penoxsulam resistance in *Echinochloa crus‐galli* and rice. (a) Y2H assays showing the interaction between EcDELLA and EcDOF2 in *E. curs‐galli*, as demonstrated by colony growth on the SD(‐LWHA) medium. (b) LCI assays in *N. benthamiana* leaves confirming EcDELLA‐EcDOF2 interaction in planta. (c) In vitro GST pull‐down assays demonstrating direct binding between MBP‐EcDELLA and GST‐EcDOF2 fusion proteins. (d) In vivo Co‐IP assays demonstrating direct binding between FLAG‐EcDELLA and HA‐EcDOF2 fusion proteins. (e–h) Y2H (e), LCI (f), GST pull‐down (g), and (h) Co‐IP assays confirming OsDELLA‐OsDOF2 interactions in rice. (i) Phenotypes of transgenic rice lines expressing *EcDELLA* (OE_rice_#3 and OE_rice_#6) and GFP responding to penoxsulam treatment. Photo was taken 21 days after penoxsulam treatment. (j) Phenotypes of rice lines overexpressing *OsDELLA* (OE_rice_#1 and OE_rice_#5) and GFP control, 21 days following penoxsulam treatment.

Yeast two‐hybrid (Y2H) assays revealed that EcDELLA physically interact with EcDOF2 (Figure 8a). Luciferase complementation imaging (LCI) assays performed in *N. benthamiana* leaves revealed a clear interaction between EcDOF2 and EcDELLA. Co‐expression of EcDOF2‐nLUC with cLUC‐EcDELLA generated intense luciferase activity, while co‐expression with the empty nLUC or cLUC constructs failed to produce any measurable signal (Figure 8b). Moreover, the interaction was further confirmed by an in vitro GST pull‐down assay using recombinant proteins purified from *E. coli*. GST‐EcDOF2 was able to precipitate MBP‐tagged EcDELLA but not the MBP control protein (Figure 8c, original blot can be found in Figure S19a). Co‐IP assays provided in vivo evidence for the physical interaction between EcDOF2 and EcDELLA (Figure 8d, original blot can be found in Figure S19b,c). Similarly, in rice, OsDELLA also physically interacted with OsDOF2. Y2H assays showed that yeast co‐expressing OsDELLA and OsDOF2 grew on SD (‐LWHA) medium, indicating a positive interaction (Figure 8e). Consistently, LCI assays in *N. benthamiana* leaves produced a strong luminescence signal when OsDELLA‐cLUC and nLUC‐OsDOF2 were co‐expressed, whereas all negative controls remained non‐luminescent (Figure 8f). Furthermore, an in vitro GST pull‐down assay using purified recombinant proteins demonstrated direct interaction between OsDOF2 and OsDELLA. GST‐OsDOF2 successfully pulled down MBP‐tagged OsDELLA, whereas no binding was detected with the MBP control protein (Figure 8g, original blot can be found in Figure S19d). Consistent with this, Co‐IP assays performed in rice protoplasts further confirmed the interaction in vivo, showing that OsDELLA was efficiently immunoprecipitated by OsDOF2 (Figure 8h, original blot can be found in Figure S19e,f), indicating that this DELLA–DOF2 interaction is likely conserved in monocot species. Competitive EMSA assays revealed that DELLA inhibited the DNA‐binding activity of DOF2 in a dose‐dependent manner. The binding of DOF2 to the promoters of *EcCYP81A21* and *OsCYP81A6* was gradually weakened upon addition of increasing amounts of DELLA, whereas DELLA alone showed no DNA‐binding activity (Figure S20). Thus, DELLA does not bind DNA directly but rather suppresses DOF2‐mediated transcriptional activation by impairing DOF2's DNA‐binding capacity through their physical interaction.

To investigate the functional role of DELLA in herbicide response, we generated *EcDELLA*‐OE_rice_ and *OsDELLA*‐OE_rice_ rice lines. Both *EcDELLA*‐OE_rice_ and *OsDELLA*‐OE_rice_ plants exhibited the typical dwarf phenotypes associated with DELLA accumulation (Figure 8i,j). When treated with 450 or 600 g ai. ha^−^^1^ of penoxsulam, the plant height of *EcDELLA*‐OE_rice_ lines (#3 and #6) was significantly reduced compared with GFP plants (Figure S21a). Upon exposure to 600 g ai. ha^−^^1^ penoxsulam, the survival rate of *EcDELLA*‐OE_rice_ plants was markedly lower than those of GFP controls (Figure S21b), indicating that elevated DELLA levels confer susceptibility to the herbicide. A similar trend was observed in *OsDELLA*‐OE_rice_ lines (#1 and #5), which also showed pronounced growth inhibition under penoxsulam treatment (Figure 8j, and Figure S21c,d). These results demonstrate that DELLA upregulation increases plant susceptibility to penoxsulam, suggesting a conserved role of DELLA proteins as negative regulators of herbicide resistance/tolerance in *E. crus‐galli* and rice. To further validate the role of DELLA (*SLR1*) [61], knockout *OsSLR1* mutants were generated (Figure S21e). Compared with WT_rice_ controls, *osslr1*‐ko_rice_ mutants exhibited stem elongation under normal growth conditions (Figure S21f), this is consistent with previous studies [61]. Following penoxsulam exposure, *osslr1*‐ko_rice_ plants displayed significantly higher survival rates and greater plant height than WT_rice_ plants (Figure S21g,h), indicating enhanced tolerance to penoxsulam.

Given that GA_3_‐induced herbicide tolerance depends on both *GID1* and *DOF2*, we further examined whether DELLA proteins are required for this GA‐mediated response. Exogenous GA_3_ was applied to *osslr1*‐ko_rice_ plants followed by penoxsulam treatment. GA_3_ application failed to enhance penoxsulam tolerance in *osslr1*‐ko_rice_ plants. No significant differences were observed between GA_3_‐treated and mock mutants in terms of fresh weight and survival rate under penoxsulam treatment (Figure S21i–k), indicating that functional DELLA proteins are indispensable for GA‐induced herbicide tolerance, supporting their essential roles in transducing GA signals to downstream detoxification pathways.

**FIGURE 9:**
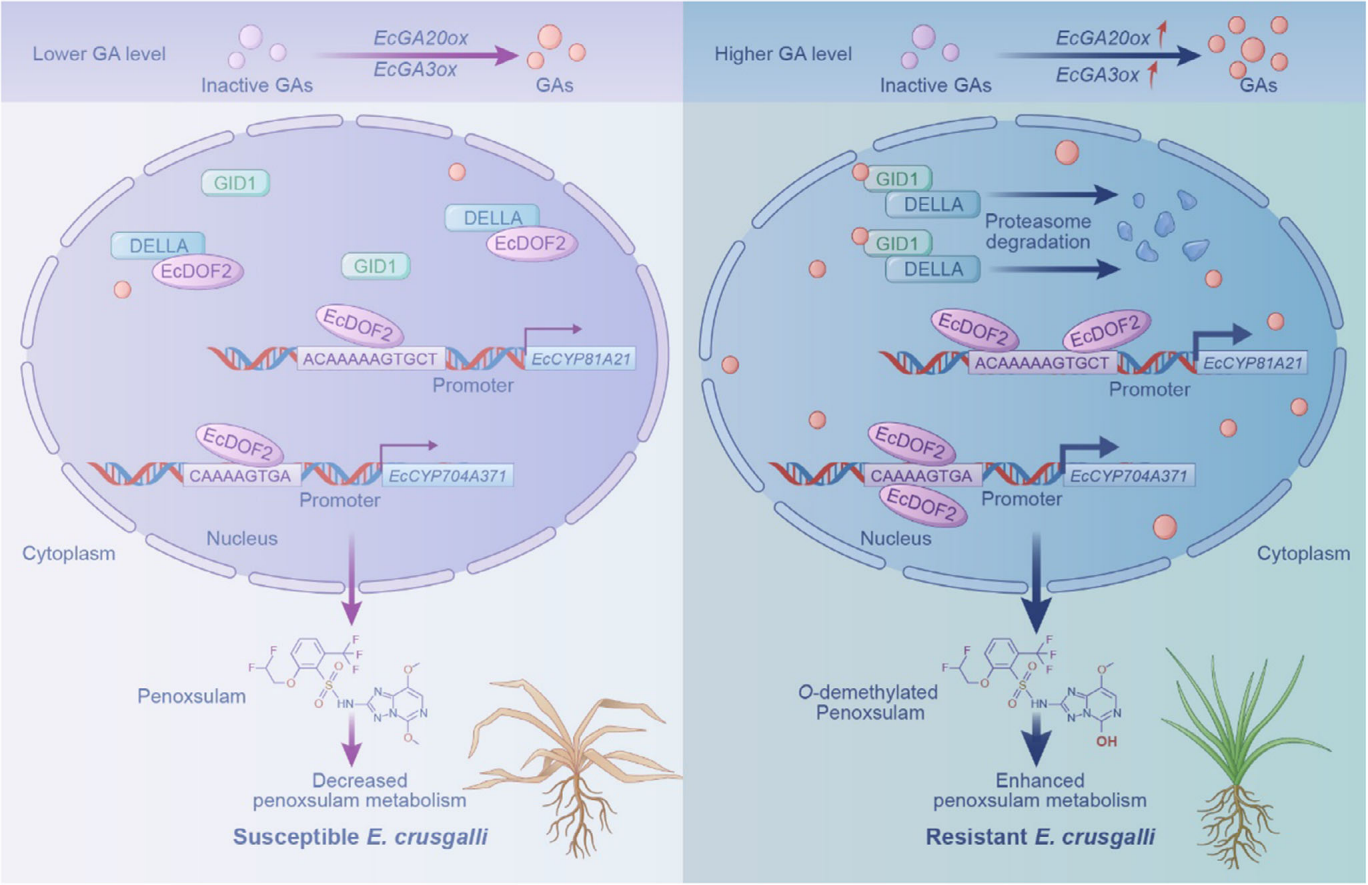
A proposed working model of the GA‐*EcDOF2*‐*EcCYP81A21*/*EcCYP704A371* signaling module mediating herbicide metabolic resistance in *Echinochloa crus‐galli*. Under lower GA conditions in susceptible *E. crus‐galli* populations, the expression of *EcDOF2* is limited, resulting in repressed transcriptional activation activity. Consequently, *EcDOF2* fails to efficiently recognize and bind to the cis‐regulatory elements within the promoters of *EcCYP81A21* and *EcCYP704A371*, thereby suppressing the transcriptional activation of downstream detoxification genes. The unelevated levels of detoxification enzymes cannot efficiently detoxify penoxsulam, leading to enhanced herbicide sensitivity.

These results support a model in which, under lower GA levels, DELLA proteins repress DOF2 activity, thereby inhibiting the expression of detoxification‐related P450 genes. Loss of DELLA function relieves this repression, promoting P450 gene expression and thus herbicide detoxification (Figure 9). Collectively, our findings reveal that DELLA acts as a negative regulator for penoxsulam tolerance in rice, functioning through a conserved GA–DELLA–DOF2 regulatory module likely shared between *E. crus‐galli* and rice.

## 3 Discussion

Unlike TSR, NTSR‐based metabolic herbicide resistance in weedy species and natural herbicide tolerance in crop species involve superfamilies of metabolic enzymes and thus likely complicated regulation networks. In recent studies, there has been an increase in identifying P450 genes endowing resistance/tolerance to ALS‐and ACCase‐inhibitor herbicides, and the next challenge is to elucidate their regulation mechanisms. Although TFs are known to be key regulators of plant abiotic stress responses [62], their roles in herbicide resistance/tolerance, particularly in regulating herbicide detoxification pathways have only recently begun to receive attention [19, 31]. However, the regulatory function of TFs in herbicide resistance, although implicated [19], remains elusive, and the mechanisms by which herbicides are sensed, along with the subsequent transduction of this signal into a physiological response mediated by TFs, have not been investigated.

It is hypothesized that herbicide application functions as an abiotic stressor that triggers intracellular signaling cascades, ultimately converging on TF families. These TFs, in turn, act as regulators of herbicide stress response genes by binding to the promoters and activating their expression, therefore, enhancing plant metabolic capacity and enabling plant survival. In this study, we demonstrate that *EcDOF2*, a key DOF transcription factor, is involved in herbicide resistance in *E. crus‐galli* and herbicide tolerance in rice, via direct activation of the major herbicide detoxification P450s in weeds (*CYP81A21* and *CYP704A371*) and in crop rice (*CYP81A6*) (Figure 5c,e and Figure S14b), thus conferring herbicide resistance/tolerance.

The ZmDOF2 protein in maize has been shown to interact with high mobility group proteins and to affect the binding efficiency of downstream target sites [63]. FaDOF2 regulated the volatile phenylpropanoid pathway in ripe strawberry receptacles by controlling the expression of the *FaEGS2* and *FaEOBII* genes, which are involved in eugenol synthesis [64]. In this study, we identified that DOF2 targets the CYP81A subfamily in both weedy and crop grass species. CYP81A is a large P450 subfamily of CYP81, genes encoding this subfamily have only been reported in grasses [65]. Members of the CYP81A subfamily, especially *CYP81A12* and *CYP81A21* in *E. phyllopongon*, and *CYP81A10v7* in *L. rigidum*, have been shown to metabolize and thus confer resistance to a broad spectrum of herbicides across different modes of action, both pre‐ and post‐emergent [8, 9, 16, 19, 66]. In rice, *CYP81A6* is the major detoxifying enzyme for the PSII‐inhibiting herbicide bentazon and ALS‐inhibiting herbicides [13, 67]. In our study, we show that DOF2 regulates *CYP81A6* (Figure S12). As for *CYP704A371*, currently, only a few genes within the CYP704 subfamily have been demonstrated to metabolize herbicides and endow resistance, including *B. syzigachne CYP704A177* ^18^ and *Raphanus raphanistrum CYP704C1* ^22^. In this study, we clearly established the role of *E. crus‐galli CYP704A371* in conferring resistance to penoxsulam (Figure 5e) under the same regulation of DOF2 as *CYP81A21*. Often, NTSR to herbicides is polygenetic, and sometimes it exhibits single gene control nature. For instance, in *E. phyllopongon*, genetic studies showed resistance to ALS‐inhibiting herbicides is segregated as a single gene trait [9]. However, more than one P450 gene (e.g., *CYP81A12*, *CYP81A21*) were identified contributing to the resistance, hence a TF was suggested to simultaneously regulate the two P450 genes [9] and thus the resistance appears as one‐gene model^.^ This may be true if a major TF regulates a few (P450) genes and each gene contributes to the resistance trait, and thus the genetic inheritance may be segregated as a monogenetic trait. Therefore, TF, rather than its downstream targets, may play a more determinant role in resistance genetic inheritance. Especially, when each of the contributing target genes has a minor role in resistance, starting with identification of their TFs may be more feasible in resistance gene discovery.

Notably, sequencing analysis confirmed that the coding sequence and promoter region of *EcDOF2* are identical in susceptible and resistant *E. crus‐galli*. Accordingly, the contrast in herbicide resistance between the two populations is not caused by sequence mutations of *EcDOF2*, but by its distinct expression regulation, which suggests the key factor driving resistance variation lies upstream of the *EcDOF2* locus. As *DOF2* is higher expressed in the penoxsulam‐resistant *E. crus‐galli* population both constitutively and herbicide inducible (Figure S1c and Figure 1b), and transgenic overexpression of *DOF2* or its downstream P450s improves penoxsulam tolerance in susceptible plants (Figure 1c,h and Figure 5a,e). Notably, although *DOF2* transcript abundance in these transgenic lines is 8–10 times higher than endogenous *DOF2* in the R populations (Figure 1b, Figures S1c and S3g), the transgenic plants still exhibit less pronounced herbicide resistance compared with the R population (Figure 1a,c). These findings indicate that the *DOF2*‐*CYP81A21*/*CYP704A371* pathway contributes significantly but not fully to penoxsulam resistance in *E. crus‐galli*. In rice, the orthologous *DOF2*‐*CYP81A6* cascade also confers partial herbicide tolerance against penoxsulam (Figure 4 and Figure S12). Given that *CYP81A21* and *CYP81A6* are well‐documented primary detoxification enzymes against multiple herbicide chemistries, *DOF2* functions as an important transcription factor contributing to herbicide metabolic resistance in these grass species, whereas additional independent regulatory pathways may be required to fully establish the complete resistance phenotype of resistant *E. crus‐galli* biotypes.

Certain DOF proteins are known to be involved in plant stress responses through interacting with various plant hormone signals [33]. Our study provides evidence and establishes a mechanistic link between GA signaling and herbicide resistance/tolerance, via the GA‐EcDOF2‐*EcCYP81A21*/*EcCYP704A371* signaling module (Figure 9). Accumulating evidence suggests that DOFs respond to various plant hormone signals, including GA, and participate in the regulation of plant stress responses. DOFs may enhance the submergence tolerance of rice during the early stages of germination by regulating GA and other plant hormones [68]. Exogenous GA_3_‐induced expression of DOFs has been demonstrated to be a key factor in the initiation of tomato fruit formation [43]. Phytochromes inhibit GA biosynthesis [69], whereas *COG1/DOF1.5* increases endogenous GA_1_ levels in *Arabidopsis* by promoting the expression of the gibberellin biosynthetic gene *GA3ox3*, which is ultimately involved in seed coat development [59]. Furthermore, the overexpression of *SlCDF4* resulted in elevated levels of *GA20ox* and *GA3ox*, thereby increasing tomato fruit size through the regulation of endogenous GA_4_ biosynthesis [70]. However, additional studies are required to investigate the roles of DOFs in GA signaling pathways and to identify which members are central to integrating the crosstalk between various hormones. Given species specificity, substantial differences exist in the types of bioactive GAs regulated by various DOFs, providing a new direction for research on traditional gibberellin pathways. Indeed, the penoxsulam ‐resistant *E. crus‐galli* population exhibited higher expression of GA biosynthesis (*GA3ox*, *GA20ox*) and receptor (*GID1*) genes, along with higher levels of active GAs (GA_1_, GA_3_, GA_4_, GA_7_) (Figure 6b–e). Thus, the intrinsically high endogenous GA level in the resistant biotype likely drives the constitutive transcriptional upregulation of *EcDOF2*, representing a distinct regulatory mechanism from the previously described GA–DELLA/GID1‐mediated control of DOF2 protein stability. However, we cannot exclude that post‐translational regulation of DOF2 protein stability via the GA–DELLA/GID1 module may also contribute to the resistant phenotype in parallel, though this was not experimentally tested in the current study. Existing studies have demonstrated that an appropriate amount of ROS modulates endogenous GA levels via Ca^2+^, NO signaling, and MAPK cascades during plant stress responses and seed germination [71, 72]. In particular, ROS can upregulate GA biosynthetic genes such as *GA3ox* via MAPK signaling cascades, thereby promoting GA biosynthesis [71, 73, 74]. Herbicides treatment triggers rapid ROS accumulation [75]. Drawing on these conserved regulatory paradigms, we propose that penoxsulam‐induced ROS production in resistant *E. crus‐galli* activates downstream signaling components including the MAPK cascade, which in turn upregulate *EcGA20ox* and *EcGA3ox* expression and elevate bioactive GA levels. The resulting GA surge then initiates the GA–DELLA‐EcDOF2‐P450 detoxification pathway to confer NTSR. Direct experimental evidence for the ROS‐MAPK‐GA regulatory axis to herbicide stress in *E. crus‐galli* is currently lacking, and this remains to be tested in future work. Consistently, exogenous GA_3_ application enhanced DOF2‐dependent activation of *CYP81A21* (Figure 6g). Conversely, in vivo inhibition of GA biosynthesis by PAC suppressed *DOF2* and its downstream detoxification genes, thereby sensitizing plants to the herbicide penoxsulam (Figure 6i). The GA‐GID1‐DELLA module has long been considered as a central regulator of plant growth, modulating downstream TF activity through sequestration and degradation of DELLA proteins [60, 76, 77]. Our data suggest that DOF2 may be one of the downstream targets relieved from *DELLA* repression following GA perception (Figure 9). Interestingly, similar hormone‐TF interactions have been observed in *Arabidopsis*, where *AtDOF1.1* (OBP2) regulates glucosinolate biosynthesis in a hormone‐dependent manner [78]. Consistent with the phenotype shown in Figure S15, exogenous GA_3_ application partially restores penoxsulam resistance in S plants and cannot reach the high resistance level observed in R plants, further demonstrating that the GA–DOF2–P450 cascade is an important but not exclusive component of the polygenic NTSR network, and additional unknown regulatory pathways are required to achieve full herbicide resistance in resistant *E. crus‐galli*.

Often, genetic transformation systems for model plants (e.g. rice, *Arabidopsis*) are used for functional validation of candidate genes for weedy species, due to the challenges establishing an equivalent system for weeds. However, model plants may not share similar genetic background and even ploidy with weedy species, and thus discrepancies may exist, especially, for studying gene transcriptional regulatory mechanisms. If we focus solely on investigating the functions of metabolic enzyme genes, we can utilize heterologous expression in model plants, as these systems can also demonstrate detoxification metabolic functions. However, transcriptional regulatory mechanisms, including the functions of transcription factors, may not be accurately identified for resistance in heterologous systems because model plants and weeds may not use the same genes, including their downstream regulatory networks. For instance, in this current study, although the DOF and the downstream targets 81A homologs are conserved in rice and *E. crus‐galli*, the *CYP704* gene was only identified in *E. crus‐galli* but not in rice. Therefore, it is essential to establish a genetic transformation system specific to the weed species under investigation. Research on *E. crus‐galli* tissue culture commenced in 1984, when white and compact embryogenic tissue was obtained from *E. crus‐galli* [79]. However, callus induction, differentiation, and regeneration in *E. crus‐galli* are time‐consuming, and the establishment of a complete genetic transformation system had not been achieved until this study (Figure S4, Figure 1c). Here, we used the first successfully generated transgenic *E. crus‐galli* lines (Figure S4) for validation of the *DOF2* gene (Figure 1c), a significant technical advancement for functional studies in weedy species. The established system enables gene functional validation for herbicide resistance and beyond. It also provides foundation for genome editing in the species, although further effort is needed to achieve this.

A key component of effective resistance management involves resistance diagnosis. However, in the case of TF‐mediated resistance, this process is often time‐consuming and costly, as identifying changes in NTSR gene expression typically requires RNA extraction and storage. This limitation also precludes the use of many common approaches for storing specimens prior to molecular analyses (e.g., in ethanol or on silica). The identification of DOF2 as a key regulatory hub in herbicide resistance provides new avenues for the development of diagnostic marker and functional genomics‐based weed control strategies. Screening for *DOF2* expression or promoter variants in weed populations could facilitate the early detection of major P450‐based metabolic resistance evolution, potentially as a marker for developing robust DNA‐based diagnostics that can be used in field‐collected samples. Such information will guide the development and implementation of strategies to control *E. crus‐galli* while minimizing ineffective herbicide applications. Additionally, DOF2 and its target genes can be potential targets for gene editing or chemical inhibition for resistance reversal. For instance, inhibitors that disrupt DOF2 binding to P450 promoters or stabilize *DELLA* repressors could synergize herbicide effects, thereby restoring herbicide efficacy. The evidence in this study that knockdown of *DOF2* and *GID1* reduces P450 expression and increases plant susceptibility to penoxsulam (Figure 7i) illustrated the potential of this approach. Furthermore, our findings indicate the risk of resistance evolution through hormone application. Application of plant growth regulators or environmental factors that upregulate GA pathways may select for resistant phenotypes. Therefore, integrated weed management strategies may have to account for hormone‐mediated plasticity in weed adaptation to environmental stresses.

Together, our findings uncover a previously unrecognized regulatory module (Figure 9) in which GA signaling activates *DOF2* to drive the expression of herbicide‐detoxifying P450 genes. This module links developmental hormone pathways to metabolic defense, providing a mechanistic basis between vigorous growth and creeping herbicide resistance/tolerance plants. By demonstrating that *DOF2* acts as a direct transcriptional activator downstream of the GA‐GID1/DELLA cascade, we establish a conceptual framework that connects hormone‐driven growth regulation with inducible xenobiotic metabolism. This GA‐GID1/DELLA‐DOF2‐P450s axis functions as an adaptive regulatory strategy validated experimentally in the tested *Poaceae* species (*E. crus‐gall*i and rice), enabling rapid reprogramming of detoxification capacity under herbicide selection. Whether this regulatory cascade is conserved across dicotyledons and other divergent plant taxa remains unconfirmed and requires further investigation in future work. Importantly, our work reveals that endogenous hormonal states can precondition herbicide responsiveness, suggesting that physiological plasticity plays roles in resistance evolution.

## 4 Materials and Methods

### 4.1 Plant Growth Conditions and Herbicide Treatment

The *E. crus‐galli* population previously characterized exhibiting metabolic resistance to cyhalofop‐butyl [54] was used in this study. For whole‐plant bioassays to determine cross‐resistance to the ALS‐inhibiting herbicide penoxsulam, and the effect of the P450 inhibitor, malathion, on the resistance, seed germination, growth conditions, and herbicide treatment were consistent with established protocols from our previous studies [24].

Rice (ZH11) seeds for transgenic studies were surface‐sterilized and germinated on a solid medium containing 0.6% (w/v) agar under controlled environmental conditions (25°C, 16 h light/8 h dark photoperiod). For solution culture, uniformly germinated seedlings were selected and transferred to half‐strength Murashige and Skoog (1/2 MS) medium supplemented with different concentrations of penoxsulam (10, 20, 40, and 100 µM). Each treatment was conducted with three biological replicates (6 seedlings as a replicate). For whole‐plant bioassays, germinated seedlings were transplanted into plastic pots containing a mixed substrate of rice medium soil and vermiculite (1:1, v/v). Plants were grown until the 3–4 leaf stage, and then treated with penoxsulam using a precision herbicide spray tower [24]. Each treatment contained 3 replicate pots and each pot had four seedlings.

*N. benthamiana* plants were cultivated in a growth chamber under controlled conditions (25°C, 70% relative humidity, and a 16‐h light/8‐h dark cycle) using a potting mixture of peat and vermiculite (2:1, v/v). These plants were used for transient gene expression assays as described in the corresponding section.

### 4.2 DNA/RNA Extraction and RT‐qPCR Assay

DNA was extracted from the leaves of *E. crus‐galli* and rice using the Plant Genomic DNA Kit (TIANGEN, Beijing, China). Total RNA was extracted from fresh *E. crus‐galli* and rice leaves using the RNA Extraction Kit (TIANGEN, Beijing, China). Total RNA was used to synthesize cDNA with the HiScript II Q RT SuperMix for qPCR (+gDNA wiper) kit (Vazyme Biotech, Nanjing, China). RT‐qPCR analysis was performed using the 2× TSINGKE Master qPCR Mix (SYBR Green I) (Tsingke Biotech, Beijing, China). Long‐chain reverse transcription was carried out using in the HiScript II 1st Strand cDNA Synthesis (+gDNA wiper) Kit. Gene‐specific primers used in the qPCR analysis are listed in Data S3. The relative expression levels of target genes were calculated using the 2^^–ΔΔCt^ method. All reactions were performed with three biological and three technical replicates.

### 4.3 Phylogenetic Analysis

DOF Transcription factor protein sequences from *Arabidopsis thaliana*, *O. sativa*, *Sorghum bicolor*, *Setaria viridis*, and *Setaria italica* were obtained from the Plant Transcription Factor Database v3.0 (PlantTFDB; http://planttfdb.gao‐lab.org/family.php?fam=DOF). A total of 162 DOF protein sequences were retrieved and used as references. The protein sequences were aligned with *E. crus‐galli* DOF2 (EcDOF2) protein sequence using the MEGA11 software. A phylogenetic tree was subsequently constructed using the neighbor‐joining method. The robustness of the tree topology was assessed by performing 1000 bootstrap replicates. The full‐length amino acid sequence of *EcDOF2* was obtained from the *E. crus‐galli* transcriptome data. To identify conserved domains, the protein sequence was analyzed using the NCBI Conserved Domain Database (CDD; https://www.ncbi.nlm.nih.gov/Structure/cdd/) and SMART (http://smart.embl.de/) with default parameters.

### 4.4 Subcellular Localization

The cDNA of *EcDOF2* was cloned into the pYba1132 vector between the *Bam*HI and *Hin*dIII restriction enzyme sites to generate the p35S‐*EcDOF2*‐eGFP construct. The empty vector and p35S‐*EcDOF2*‐eGFP construct were separately transformed into the *Agrobacterium tumefaciens* strain GV3101. After positive clone selection, bacterial cells were resuspended in the infiltration solution (10 mM MES, 10 mM MgCl_2_, 150 µM acetosyringone, pH 5.6) and adjusted to an OD_600_ of 0.6. The suspension was infiltrated into 4‐ to 5‐week‐old tobacco leaves, and fluorescence signals were observed using confocal microscopy 48 h post‐infiltration. Fluorescence signals were observed using a Leica TCS SP5 confocal laser scanning microscope (Leica, Germany). The excitation wavelength was set at 488 nm, and emission was detected within the range of 5000–550 nm. The primers used for construct generation were listed in Data S3.

### 4.5 Transcription Activation Analysis

To identify the transcriptional activation domain in *EcDOF2*, the full‐length, C‐terminal, and N‐terminal regions of *EcDOF2* were amplified and inserted into the pGBKT7 vector, generating recombinant plasmids pGBKT7‐*EcDOF2*, pGBKT7‐*EcDOF2*‐N, and pGBKT7‐*EcDOF2*‐C. The pGBKT7 empty vector and recombinant plasmids were separately transformed into Y2HGold yeast cells. The yeast cultures were adjusted to an OD_600_ of 0.2 and subsequently plated onto SDO (SD/‐Trp), TDO (SD/‐Trp‐His‐Ade), and TDO/x‐α‐gal (SD/‐Trp‐His‐Ade + x‐α‐gal) media. The plates were incubated at 28°C for 3 days in an inverted position to allow colony growth and color development. The primers used for vector construction were listed in Data S3.

### 4.6 Generation of Transgenic *E. crus‐galli* and Rice Plants

The ORF of *EcDOF2* was cloned into the pOX overexpression vector to generate the pro35S:*EcDOF2* construct, which was subsequently introduced into herbicide susceptible *E. crus‐galli* calli via agrobacterium‐mediated transformation using strain EHA105. After 3 days of co‐cultivation, transformed calli were subjected to two rounds of hygromycin selection, followed by subculture and subsequent differentiation and rooting. *EcDOF2*‐overexpressing (*EcDOF2*‐OE_EC_) *E. crus‐galli* lines were confirmed by PCR amplification and RT‐qPCR analysis. Homozygous *EcDOF2*‐OE_EC_ plants were selected and used for whole‐plant phenotypic bioassays. Full experimental procedures are provided in Method S1.

For rice transformation, the ORFs of *EcDOF2*, *EcCYP81A21*, *EcCYP704A371*, and their orthologs (*OsDOF2* and *OsCYP81A6*) in rice were amplified and cloned into the pOX vector to generate overexpression constructs (pOX*‐EcDOF2*, pOX*‐EcCYP81A21*, pOX‐*EcCYP704A371*, pOX‐*OsDOF2*, and pOX‐*OsCYP81A6*, respectively). The constructs were introduced into *A. tumefaciens* strain EHA105 and transformed into rice (ZH11) [19]. Primers used for vector construction and RT‐qPCR analyses were listed in Data S3.

### 4.7 Generation of *OsDOF2* Knockout Rice Mutant

The *OsDOF2* (LOC_Os01g15900) knockout mutant lines (*osdof2*‐ko_rice_) were generated using CRISPR‐Cas9. A 23bp target sequence within the *OsDOF2* gene was selected and confirmed via BLAST analysis in the rice genome database (GGTGATCCCGCTGGCTCCAGAGG), then integrated into the pGBK032 vector. This construct was transferred into rice through Agrobacterium‐mediated transformation to generate T1 transgenic plants. Specific primers were designed flanking the target sequence, and DNA extraction and sequencing were performed on all T1 transgenic plants to identify homozygous lines for generation of homozygous T2 transgenic lines (*osdof2*‐ko_rice_). Which were used for herbicide sensitivity screening with WT_rice_ as control plants.

### 4.8 RNA‐Seq Analysis to Identify DEGs in the *EcDOF2*‐OE_EC_ *E. crus‐galli* and *osdof2*‐ko_rice_ Rice Plants

For RNA‐seq analysis, total RNA was extracted from the leaf tissue of the 3–4 leaf stage plants, including *EcDOF2*‐OE_EC_ (line#5, line#7) and *osdof2* knockout rice mutants, and their respective WT counterparts. Each genotype had three biological replicate plants. RNA quality was assessed prior to library preparation, which involved mRNA enrichment, followed by sequencing on an Illumina platform (Illumina novaseq 6000). Sequencing reads were subjected to quality checks, trimming, and alignment to the respective reference genomes. The DESeq2 software package was used to determine differentially expressed genes (DEGs) in an R environment. The adjusted *p* value < 0.05, and the cutoff value log2 (fold change) ≥1 or ≤ −1 were defined as differentially expressed genes. Functional enrichment analyses, including Gene Ontology (GO) and KEGG pathway analysis, were performed on the differentially expressed genes.

### 4.9 DAP‐Seq and Data Analysis

Genomic DNA libraries were prepared as previously described [80]. The *EcDOF2* coding region was cloned into the pFN19K Halo Tag T7 SP6 Flexi vector, and the Halo‐*EcDOF2* fusion protein was produced using the TNT SP6 Coupled Wheat Extract System (Promega). Produced proteins were directly captured using Magne Halo Tag Beads (Promega). Captured protein‐bound DNA samples were tagged with dual‐indexed multiplexing barcodes and sequenced on the Illumina HiSeq 2500 platform, generating single‐end reads of 150 bp. Quality check on the raw reads was performed using FastQC (v0.11.9), followed by trimming with Trimmomatic [81]. The trimmed reads were mapped to the *E. crus‐galli* reference genome (*E. crus‐galli* V2.0), and peak identification conducted using MACS [82]. The core motif discovery was performed using MEME‐ChIP [83], and the identified genes were subjected to KEGG pathway classification analysis.

### 4.10 Yeast One‐Hybrid (Y1H) Assays

The promoter regions of the *EcCYP81A21* and *EcCYP704A371*, and *OsCYP81A6* and *OsCYP704C1* genes were amplified from *E. crus‐galli* and rice genomic DNA, respectively. The full‐length coding sequences of *EcDOF2* and *OsDOF2* genes were cloned into the pAbAi bait vector and the pGADT7 prey vector. The pAbAi‐*EcCYP81A21* or *‐OsCYP81A6*, pAbAi‐*EcCYP704A371* or ‐*OsCYP704C1*, and pGADT7‐*EcDOF2* or ‐*OsDOF2* plasmids were co‐transformed into Y1H Gold yeast cells, and then cultured at 30°C on SD/‐Leu and SD/‐Leu + AbA^100^ media for 3–5 days [19]. The primers used for vector construction were listed in Data S3.

### 4.11 Electrophoretic Mobility Shift Assay (EMSA)

To conduct EMSA, His‐tagged EcDOF2 and OsDOF2 proteins were expressed according to published research [19]. Briefly, the coding sequences of *EcDOF2* and *OsDOF2* were cloned into the pET28a expression vector, and recombinant proteins expressed in *E. coli* BL21 (DE3) cells. Protein expression was induced by adding IPTG to a final concentration of 1 mM, followed by incubation at 28°C and 200 rpm for 20 h. His‐tagged fusion proteins were purified using a His‐tagged protein purification kit (Colarbo, Shanghai, China). Biotin‐labeled DNA probes containing the putative DOF binding sites were synthesized from the promoter regions of *EcCYP81A21* (29 bp), *EcCYP704A371* (29 bp), and *OsCYP81A6* (29 bp). Unlabeled probes were used as cold competitors, and mutated versions were generated by substituting core binding site sequences. EMSA detection was performed using the LightShift Chemiluminescent EMSA kit (Thermo Fisher Scientific, Waltham, MA, USA), and the results were visualized using the chemiluminescent imaging system (Bio‐Rad, Hercules, CA, USA). Probe sequences were listed in Table S7.

### 4.12 Dual‐Luciferase Assays

The dual‐luciferase assay was performed as described [19]. The full‐length coding sequences of *EcDOF2* and *OsDOF2* were cloned into the pGreen II 62‐SK vector as effectors, while the promoter regions of *EcCYP81A21*, *EcCYP704A371*, and *OsCYP81A* were inserted into the pGreen II 0800‐LUC vector as reporters. All constructs were verified by sequencing and introduced into *A. tumefaciens* strain GV3101. The corresponding effector and reporter plasmids were co‐infiltrated into *N. benthamiana* leaves using transient transformation. Forty‐eight hours after incubation, the leaves were sprayed with 0.1 M luciferin and kept in the dark for 5 min. Luminescence was detected using the low‐light cooled CCD imaging system (CHEMIPROHT 1300B/LND, 16 bits; Roper Scientific, Martinsried, Germany). The primers used for Dual‐LUC assays were listed in Data S3.

### 4.13 Exogenous Application of GA and Quantification

To investigate the response of *EcDOF2* to gibberellin, the 3–4 leaf stage seedlings of penoxsulam ‐susceptible and ‐resistant *E. crus‐galli* were selected for exogenous GA_3_ treatment. Seedlings were foliar‐sprayed with 100 µM GA_3_ solution, while water‐treated plants served as control. Leaf samples were collected at 0, 6, 12, 24, and 48 h post‐treatment, immediately frozen in liquid nitrogen, and stored at −80°C for subsequent RNA extraction. The expression levels of *EcDOF2* were quantified by RT‐qPCR. Each treatment group included three biological replicate samples, and each sample was analyzed in three technical replicates.

For GA quantification, ESI‐HPLC‐MS/MS analysis were performed according Pan with some modification [84]. Briefly, the harvested leaf tissue was ground into a fine powder in liquid nitrogen. A 0.5 g aliquot of tissue was transferred into a 15 mL centrifuge tube and extracted with 10 mL of acetonitrile containing 8 µL of deuterated GA4 (D‐GA4) as an internal standard. Samples were extracted overnight at 4°C, then centrifuged at 12 000 × g at 4°C for 5 min. The supernatant was collected, and the residue was re‐extracted twice with 5 mL of acetonitrile. The resulting supernatant was dried under nitrogen gas and reconstituted in 400 µL of methanol. The solution was filtered through a 0.22 µm organic‐phase membrane and stored at −20°C until analysis.

Chromatographic separation was carried out on a Poroshell 120 SB‐C18 reversed‐phase column (2.1 mm × 150 mm, 2.7 µm; Agilent Technologies) maintained at 30°C. The mobile phase consisted of solvent A (water with 0.02% formic acid) and solvent B (HPLC‐grade methanol) at a flow rate of 0.3 mL/min^−1^. The injection volume was 10 µL. Mass spectrometric detection was performed using electrospray ionization in both positive and negative ion switching mode. Instrument settings included an ion source temperature of 300°C, interface heating at 300°C, and ionization voltages of +5500 and −5000 V. Authentic standards of GA_1_, GA_3_ GA_4_, GA_7_, and D‐GA_4_ (≥98% purity) were obtained from Sigma–Aldrich (St. Louis, MO, USA).

### 4.14 Identification and Analysis of Genes in the GA Pathway in *E. crus‐galli*

To investigate genes potentially involved in the GA biosynthesis pathway, previously published transcriptome data from herbicide‐resistant and ‐susceptible *E. crus‐galli* populations [54] were analyzed. Candidate GA‐related DEGs were validated by RT‐qPCR. Total RNA extraction, cDNA synthesis, qPCR procedures, and relative quantification of gene expression were as described previously. The primer sequences used in the RT‐qPCR validation were listed in Data S3.

### 4.15 Combined Effect of GA Inhibitor PAC and Penoxsulam Treatment

The 3–4 leaf stage, herbicide‐resistant *E. crus‐galli* plants were foliar treated with (1) water, (2) a known GA inhibitor, paclobutrazol solution [60] (100 µM PAC), (3) penoxsulam at the recommended field rate (10 g ai. ha^−^^1^), and (4) a mixture of PAC and penoxsulam. Each treatment included three biological replicates with 5 plants per replicate. Plants were maintained under controlled conditions (25°C, 16 h light/8 h dark photoperiod, 60% relative humidity). Survival rates were assessed 14 days after treatment, and the plants having newly expanded leaves from the shoot apex were considered as survivors.

### 4.16 Sensitivity of *OsGID1*/*DELLA* Mutants to Penoxsulam

To investigate the role of GA signaling in herbicide resistance, we employed the CRISPR‐Cas9 genome‐editing system to generate rice knockout mutants targeting key components of the GA pathway. For positive regulation, the GA receptor gene *OsGID1* (LOC_Os05g33730) was targeted using a specific sgRNA sequence (5′‐TGTCGTACAACATTCTGCGGCGG‐3′), inserted into the CRISPR‐Cas9 binary vector. Similarly, to disrupt negative regulation, the *DELLA* gene (*SLR1*, LOC_Os03g49990), a known repressor of GA signaling, was edited using the sgRNA sequence (5′‐GTTCGTGTCGCACCTGGCCACGG‐3′). Homozygous knockout mutant lines for *osgid1* and *slr1* were confirmed by sequencing and selected for subsequent phenotypic and herbicide sensitivity analysis.

WT_rice_ and *osgid1* mutant rice seeds were germinated and grown in glass test tubes containing 1/2 MS medium supplemented with different concentrations of penoxsulam (0, 10, 20, 30, and 40 µM). Each treatment included three biological replicates. Fourteen days after growth under controlled conditions (28°C, 16 h light/8 h dark), shoot length and fresh weight were measured.

To further assess penoxsulam sensitivity at the whole‐plant level, WT_rice_, *osgid1*‐ko_rice_, and *slr1*‐ko_rice_ seedlings were grown in pots to the 3–4 leaf stage and then treated with penoxsulam at 0, 450, and 600 g ai. ha^−^^1^ using the spray tower as described before. Each treatment had three replicates with at least 10 plants per replicate. Fresh weight was recorded 14 days after treatment.

To evaluate the transcriptional response, RT‐qPCR was performed to analyze the expression of *OsGID1*, *OsDELLA*, *OsDOF2*, and *OsCYP81A6* in WT_rice_ and *osgid1*‐ko_rice_ *or slr1*‐ko_rice_ seedlings. All reactions were conducted with three biological replicates and three technical replicates per replicate. The relative expression levels were calculated using the 2^–ΔΔCt^ method. Primer sequences used were listed in Data S3.

### 4.17 Protein‐Protein Interaction Assays

#### 4.17.1 Yeast Two‐Hybrid Assay (Y2H)

To test physical interactions between DOF2 and DELLA proteins, full‐length coding sequences of EcDOF2, EcDELLA, OsDOF2, and OsDELLA were cloned into the pGBKT7 (DNA‐binding domain, BD) and pGADT7 (activation domain, AD) vectors (Clontech). Recombinant plasmid pairs (BD and AD) were co‐transformed into Saccharomyces cerevisiae strain Y2HGold using the lithium acetate/PEG method. Transformants were first selected on SD/‐Leu/‐Trp (SD(‐LW)) medium and then transferred to SD/‐Leu/‐Trp/‐His/‐Ade (SD (‐LWHA)) medium to assess protein–protein interaction. Positive interactions were indicated by colony growth on SD (‐LWHA) after 3–4 days of incubation at 30°C.

#### 4.17.2 Luciferase Complementation Imaging (LCI) Assay

The coding sequences of EcDOF2, EcDELLA, OsDOF2, and OsDELLA were cloned into pCAMBIA1300 vectors containing the N‐terminal (nLUC) and C‐terminal (cLUC) fragments of firefly luciferase, respectively. Constructs were co‐infiltrated into *N. benthamiana* leaves using *A. tumefaciens* GV3101 for 48 h. Leaf disks were then incubated with 1 mM luciferin (solarbio), and luminescence was captured using the CCD imaging system (Tanon 5200). The interaction strength was quantified using the ImageJ software. Negative controls included co‐infiltration of DELLA‐nLUC and DOF2‐cLUC with empty vectors.

#### 4.17.3 Co‐Immunoprecipitation (Co‐IP)

For in vivo confirmation of interaction, EcDELLA and EcDOF2 (as well as rice orthologs) were cloned into vectors with C‐terminal 3×FLAG or MYC tags. Plasmids were co‐transfected into rice protoplasts via PEG‐mediated transformation. Total protein was extracted with IP buffer (50 mM Tris‐HCl pH 7.5, 150 mM NaCl, 1% NP‐40) containing protease inhibitors. Immunoprecipitation was performed using anti‐FLAG (MBL, M185‐3L), anti‐HA (Abcam, Ab9110) and anti‐MYC antibodies (Abclonal, AE070) followed by immunoblotting. Input and immunoprecipitated samples were analyzed using SDS‐PAGE and Western blot.

#### 4.17.4 GST Pull‐Down Assay

Pull‐down assays were performed using recombinant proteins expressed in *E. coli*. DELLA was fused to GST using pGEX‐4T‐1 and expressed in *E. coli* BL21 (DE3), while DOF2 was fused to His‐tag using pET‐32a (+). Proteins were purified using Glutathione Sepharose 4B resin (GE Healthcare) and Ni‐NTA agarose, respectively. GST‐DELLA or GST alone was incubated with MBP‐DOF2 in the binding buffer (20 mM Tris‐HCl pH 7.5, 150 mM NaCl, 1 mM DTT) at 4°C for 2 h. The resin was washed extensively, and bound proteins were analyzed by western blotting using anti‐MBP (Proteintech, 66003‐1‐Ig) and anti‐GST (YEASEN, 30901ES50) antibodies.

### 4.18 Statistical Analyses

Statistical analyses were conducted using GraphPad Prism version 10.0. Statistical significance was evaluated using a two‐tailed Student's *t*‐test for comparisons between two groups, while one‐way ANOVA or two‐way ANOVA was performed for multiple‐group comparisons. All detailed parameters and relevant information for statistical analyses are described in the corresponding figure legends.

## Author Contributions

J. W. conceptualization, methodology, investigation, data curation, and the writing of the original draft and its review and editing. J. Q. conceptualization, methodology, and validation. L. W. validation. Q. Y. review and editing of the manuscript. Z. L. methodology and formal analysis. L. B. conceptualization and resources. L. P. conceptualization, funding acquisition, and the writing of the original draft and its review and editing.

## Conflicts of Interest

The authors declare no conflicts of interest.

## Supporting information




**Supporting File 1**: advs76742‐sup‐0001‐SuppMat.docx.

**Supporting File 2**: advs76742‐sup‐0002‐DataS1‐S3.zip.

**Supporting File 3**: advs76742‐sup‐0003‐MethodS1.docx.

## Data Availability

The data that support the findings of this study are available from the corresponding author upon reasonable request.
