# Supplementary material for: Herbicide Metabolic Resistance in Poaceae Plants via the GA‐GID1/DELLA‐DOF2‐P450s Module
Source: Adv Sci (Weinh). 2026 Jul 24;13(57):e76742. doi: 10.1002/advs.76742 (PMC13398137; doi:10.1002/advs.76742)
Supplement: Supplementary file 2 [file ADVS-13-e76742-s003.docx]

**Title:**

**Herbicide metabolic resistance in *Poaceae*** **plants via the GA-GID1/DELLA-DOF2-P450s module**

Junzhi Wang^1,2,3,#^, Jiale Qi^1,#^, Lin Wei^1^, Qin Yu^3^, Zhongyuan Liu^1^, Lianyang Bai^1,2,3,*^, Lang Pan^1,3,*^

**Affiliations:**

^1^ College of Plant Protection, Hunan Agricultural University, Changsha, 410128, China

^2^ Hunan Plant Protection Research Institute, Hunan Academy of Agricultural Sciences, Changsha, 410125, China

^3^ Yuelushan Laboratory, Changsha, 410016, China

^#^ These authors contributed equally.

**^*^ Correspondence:** Lang Pan (Email: langpan@hunau.edu.cn), Lianyang Bai (Email: [lybai@hunaas.cn)](mailto:lybai@hunaas.cn))

**Supplemental figures and legends**

**Supplemental Fig. 1 | Identification of candidate TFs involved in penoxsulam resistance in *Echinochloa crus-galli*. a.** Volcano plot of transcriptome comparisons between R and S populations. **b.** Heatmap showing transcription factors differentially expressed between R and S samples. **c.** Relative expression levels of *EcDOF2* in R and S plants without penoxsulam treatment. **d.** Alignment of *EcDOF2* coding sequences from R and S samples reveals several synonymous SNPs that do not alter the amino acid sequence. Asterisks denote significant differences. (**p* < 0.05, ****p* < 0.001, *****p* < 0.0001). Two-tailed Student’s t-test was performed for (**c**).

**Supplemental Fig. 2 |** **Sequence alignment of the promoter of *EcDOF2* in S and R plants.**

**Supplemental** **Fig. 3 | Characterization of EcDOF2 in *Echinochloa crus-galli*.** **a.** Phylogenetic tree of EcDOF2 protein and its homologs from *Arabidopsis thaliana*, *Oryza sativa*, *Sorghum bicolor*, *Setaria viridis*, and *Setaria italica*. The tree was constructed using the Neighbor-Joining method in MEGA based on multiple sequence alignment by ClustalW. Bootstrap values from 1000 replicates are shown next to the branches. **b.** Phylogenetic tree of EcDOF2 and selected DOF transcription factors from *O. sativa*. Tree was constructed using the neighbor-joining method with full-length protein sequences, branch colors denote major clades. **c.** Schematic representation of EcDOF2 protein structure, showing the conserved DOF DNA-binding domain, predicted nuclear localization signal (NLS), and C-terminal activation domain. **d.** Subcellular localization of EcDOF2-GFP fusion protein in *Nicotiana benthamiana* leaf epidermal cells. GFP fluorescence indicates nuclear localization. Scale bar, 50 μm. **e.** Transcriptional activation analysis of EcDOF2 and its truncated forms in yeast cells.

**Supplemental Fig. 4 | Generation of *EcDOF2*-OE transgenic lines in *Echinochloa crus-galli*.** **a.** Schematic representation of the Ubi::*EcDOF2* overexpression construct, containing the *35S* promoter and the *Nos* terminator. **b.** Callus induction from *E. crus-galli* seeds. **c.** Subculture of induced calli for proliferation. **d.** Agrobacterium-mediated transformation of seed-derived calli using the Ubi::*EcDOF2* construct. **e.** Regenerated plantlets showing shoot differentiation from *EcDOF2*-transformed calli. **f.** PCR-based genotyping of T0 hygromycin-resistant plants transformed *EcDOF2*. **g.** RT-qPCR analysis showing elevated *EcDOF2* transcript levels in representative transgenic lines.

**Supplemental Fig. 5 | Fresh weight inhibition of plants treated with 15 g a.i. ha⁻¹ penoxsulam.** **a.** Comparison of fresh weight inhibition between S and R *Echinochloa crus-galli*. Statistical analysis was performed using Student’s t-test. **b.** Fresh weight inhibition of WT_Ec_ and two *EcDOF2*-OE_Ec_ transgenic rice lines (OE_EC_#5, OE_EC_#7). Two-tailed Student’s t-test was performed for (**a, b**). Asterisks indicate significant differences: ***p* < 0.01; ****p* < 0.001; *****p* < 0.0001.

**Supplemental Fig. 6 | Amino acid sequence alignment of DOF2 from *Echinochloa crus-galli* and rice accessions.**

**Supplemental Fig. 7 | Genome-wide identification of EcDOF2 binding sites by DAP-seq.** **a.** Venn diagram showing overlap of EcDOF2 binding peaks from two biological DAP-seq replicates, identifying 63,036 high-confidence shared peaks. **b.** Genomic distribution of EcDOF2 binding peaks relative to TSSs, revealing strong enrichment in promoter-proximal regions (−2 kb to 0). **c.** The distribution of EcDOF2 binding peaks on the genome. **d.** Chromosomal distribution of EcDOF2-binding peaks identified by DAP-seq using the EcDOF2-HaloTag fusion protein. **e.** The DNA-binding motifs identified by MEME-ChIP for EcDOF2-enriched binding sites, with E-values shown.

**Supplemental Fig. 8 | Transcriptome-based identification of EcDOF2-regulated genes in *Echinochloa crus-galli*.** **a, b.** Volcano plots showing DEGs in EcDOF2-overexpressing lines OE_EC_#5 (**a**) and OE_EC_#7 (**b**) compared with the WT_EC_. DEGs were defined as genes with |Log₂FC| > 1 and FDR < 0.05. Red and blue dots indicate significantly upregulated and downregulated genes, respectively. **c.** Heatmap representation of the expression profiles of the eight P450 genes in WT_EC_ and *EcDOF2*-OE_EC_ lines.

**Supplemental Fig. 9 | Analysis of OsDOF2 target genes by RNA-seq. a.** Volcano plot showing DEGs between WT and *osdof2* knockout mutant rice. DEGs were identified based on adjusted *p* < 0.05 and |Log₂FC| > 1. **b.** KEGG pathway enrichment analysis of *OsDOF2*-regulated DEGs. **c.** Heatmap showing significantly differentially expressed cytochrome P450 genes between WT and *osdof2* mutant rice plants.

**Supplemental Fig. 10 | KEGG pathway enrichment of genes defined by the association of DAP-seq peaks with RNA-seq differential expression.** **a.** GO enrichment analysis of 446 putative direct EcDOF2 targets. Dot color indicates enrichment significance (−log₁₀(*P*-value)), and dot size represents the number of genes per term. **b.** KEGG pathways based on the overlapping genes between DAP-seq targets and RNA-seq differentially expressed genes.

**Supplemental Fig. 11 |** **a.** Amino acid sequence alignment of EcCYP81A21 and EpCYP81A21**. b.** Amino acid sequence alignment of EcCYP81A21 and OsCYP81A6**.** **c.** Amino acid sequence alignment of EcCYP704A371 and OsCYP704C1.

**Supplemental Fig. 12 | OsDOF2 directly activates *OsCYP81A6* expression but does not interact with *OsCYP704C1*.** Relative expression levels of *OsCYP81A6* (**a**) and *OsCYP704C1* (**b**) in WT_rice_ and *osdof2*-ko_rice_ plants. **c.** Y1H assay showing the interaction between OsDOF2 and the promoter region of *OsCYP81A6*. **d, e.** Dual-LUC/REN demonstrating the transactivation of the *OsCYP81A6* promoter by OsDOF2 in *N. benthamiana* leaves. LUC/REN ratios were quantified, data are means ± SD (n = 3). **f.** EMSA showing specific binding of purified OsDOF2–His to a biotin-labeled probe containing the TGAAAAGGTG motif in the *OsCYP81A6* promoter. Binding was abolished by competition with excess unlabeled but not mutated probe. **g**. Lack of interaction between OsDOF2 and the *OsCYP704C1* promoter in the Y1H. **h**. Dual-LUC/REN verified the interaction between OsDOF2 and the *OsCYP704C1* promoter. Statistical significance was assessed using two-tailed Student’s *t*-test (***p* < 0.01, ****p* < 0.001).

**Supplemental Fig. 13 | Overexpression of *EcDOF2* confers resistance to pinoxaden and bensulfuron-methyl in *Echinochloa crus-galli*.** **a.** Phenotypes of WT_EC_ and *EcDOF2*-OE_EC_ lines (#5 and #7) under different rates of pinoxaden. **b.** Plant height of WT and *EcDOF2*-OE_EC_ lines in response to pinoxaden treatment. **c.** Fresh weight of WT_EC_ and *EcDOF2*-OE_EC_ lines under pinoxaden treatment. **d.** Phenotypes of WT_EC_ and *EcDOF2*-OE_EC_ lines exposed to different rates of bensulfuron-methyl. **e.** Plant height of WT_EC_ and *EcDOF2*-OE_EC_ lines following bensulfuron-methyl treatment. **f.** Fresh weight of WT_EC_ and *EcDOF2*-OE_EC_ lines under bensulfuron-methyl treatment. WT_EC_ plants showed severe growth inhibition and death under high rates of both herbicides, whereas *EcDOF2*-OE_EC_ plants retained significantly greater plant height and biomass. Data are shown as mean ± SD (n=12). Two-way ANOVA followed by Dunnett’s multiple comparison test was performed for (**b, c, e, f,**). (**** *p* < 0.001).

**Supplemental Fig. 14 | Overexpression of *OsCYP81A6* in rice increases penoxsulam tolerance.** **a.** RT-qPCR quantification showing elevated *OsCYP81A6* expression levels in *OsCYP81A6*-OE_rice_ lines. **b**. Whole-plant herbicide assays reveal increased tolerance to penoxsulam in *OsCYP81A6* overexpressing lines (OE_rice_#1 and OE_rice_#3) compared to GFP. Photo was taken 21 d after treatment. **c.** Survival rate assessment indicates enhanced tolerance in *OsCYP81A6*-OE_rice_ lines. **d.** Quantification of plant fresh weight following penoxsulam treatment. Data are mean ± SD. Statistical significance was determined using two-tailed Student’s t-test for (**a**); Two-way ANOVA followed by Dunnett’s multiple comparison test was performed for (**c**, **d**). Asterisks indicate significant differences (****p* < 0.001, *****p* < 0.0001).

**Supplemental Fig. 15 | Gibberellin positively regulates penoxsulam resistance in *Echinochloa crus-galli*. a.** Relative expression levels of *EcGA20ox*, *EcGA3ox*, and *EcGID1* in R and S plants. **b.**Time-course expression analysis of *EcCYP704A371* in *E. crus-galli* leaves following GA₃ treatment (20 μM). **c–f.** Quantification of endogenous gibberellins GA_1_ (**c**), GA_3_ (**d**), GA_4_ (**e**), and GA_7_ (**f**) in *E. crus-galli* following PAC treatment, hormone levels were measured by LC-MS/MS. Fresh weight (**g**) and plant height (**h**) of the R plants as affected by PAC treatment. **i.** Representative phenotypes of penoxsulam susceptible *E. crus-galli* seedlings treated with penoxsulam in the presence (GA₃) or absence (Mock) of exogenous GA₃. Quantification of plant height (**j**) and fresh weight (**k**) of seedlings under the same treatments. Data are mean ± SD. Statistical significance was determined using two-tailed Student’s t-test for (**a, c, d, e, f**); Two-way ANOVA followed by Sidak’s multiple comparison test was performed for (**b,** **g, h, j, k**). Asterisks indicate significant differences (***p* < 0.01, ****p* < 0.001, *****p* < 0.0001).

**Supplemental Fig. 16 | GA₃ fails to enhance penoxsulam tolerance in *osdof2*-ko_rice_.** **a.** Representative phenotypes of *osdof2*-ko_rice_ plants treated with penoxsulam in the presence (GA₃) or absence (Mock) of exogenous GA₃. Fresh weight (**b**) and survival rate (**c**) under the same treatment conditions. **d.** Quantification of the relative expression levels of *OsDOF2* and *OsCYP81A6* in *osdof2*-ko_rice_ rice under the same GA₃ treatment conditions. Data are presented as mean ± SD (n = 12 biological replicates). Two-way ANOVA followed by Dunnett’s multiple comparison test was performed for **b**, Two-tailed Student’s t-test for (**c, d**). ns, not significant.

**Supplemental Fig. 17 | Characterization of *osgid1* mutants and their response to penoxsulam treatment. a.** Schematic representation of the *OsGID1* gene structure and the CRISPR/Cas9-targeted site. **b.** Sanger sequencing chromatograms showing a 1 bp (A) insertion in the *osgid1* mutants. **c.** Survival rate of WT_rice_ and *osgid1* mutants grown under 20 or 40 μM penoxsulam. **d.** Plant height of WT_rice_ and *osgid1* mutants grown under various concentrations of penoxsulam (0-40 μM). **e.** Plant height of WT _rice_ and *osgid1* mutants treated with different field application rates of penoxsulam (0-600 g ai. ha⁻¹). **f.** Representative phenotypes of *osgid1*-ko_rice_ seedlings treated with penoxsulam in the presence (GA₃) or absence (Mock) of exogenous GA₃. Quantification of fresh weight (**g**) and survival rate (**h**) under the same treatments. Data are as mean ± SD (n = 6–12). Asterisks indicate significant differences compared to WT (***p* < 0.01, ****p* < 0.001, *****p* < 0.0001, Two-way ANOVA followed by Dunnett’s multiple comparison test was performed for (**c**, **d**, **e),** two-tailed Student’s t-test for **g** and **h**; ns, not significant.).

**Supplemental Fig. 18 | Amino acid sequence alignment of DELLA from** ***E. crus-galli* and rice accessions.**

**Supplemental Fig. 19 | Unmodified full original blots for GST pull-down and Co-IP assays.** **a.** Full blots of GST pull-down for *E. crus-galli* EcDOF2 and EcDELLA. **b, c.** Complete raw blots for EcDOF2-EcDELLA Co-IP experiments. **d.** Uncropped full blots of GST pull-down for rice OsDOF2 and OsDELLA. **e, f.** Original full immunoblots of OsDOF2-OsDELLA Co-IP assays.

**Supplemental Fig. 20 | EMSA demonstrating the inhibitory effect of DELLA on DOF2 DNA-binding activity. a.** Interaction of EcDOF2 with the *EcCYP81A21* promoter probe in the presence of increasing concentrations of EcDELLA (1×, 2×). **b.** Interaction of OsDOF2 with the *OsCYP81A6* promoter probe in the presence of increasing concentrations of OsDELLA (1×, 2×).

**Supplemental Fig. 21 | Functional characterization of DELLA in herbicide response.** **a-d.** Plant height and survical rate of transgenic rice plants *EcDELLA*-OE_rice_ (**a, b**) or *OsDELLA*-OE_rice_ (**c, d**) after treatment with 600 g ai. ha⁻¹ penoxsulam. **e.** Sanger sequencing chromatograms showing the single-nucleotide substitution (G→T) introduced in *OsSLR1* by CRISPR/Cas9-mediated mutagenesis. **f.** Phenotypes of *osslr1*-ko_rice_ mutant plants and WT_rice_ controls treated with 0, 600, or 750 g ai. ha⁻¹ penoxsulam. **g, h.** Quantification of the survival rate (**g**) and plant height (**h**) of *osslr1*-ko_rice_ lines (#2 and #5) compared with WT_rice_ plants. **i.** Phenotypic responses of *osslr1*-ko_rice_ seedlings treated with penoxsulam in the presence or absence of exogenous GA₃. Fresh weight (**j**) and survival rate (**k**) in *osslr1*-ko_rice_ mutants. Data are mean ± SD. Statistical significance was determined by two-tailed Student’s *t*-test for (**b, d, g, k**), Two-way ANOVA followed by Dunnett’s multiple comparison test was performed for (**a, c, h, j**). (**p* < 0.05; ***p* < 0.01; ****p* < 0.001; *****p* < 0.0001). ns, not significant.

**Supplemental Table 1. Effects of the P450 inhibitor (Malathion) on growth response to penoxsulam of R and S *E. crus-galli* populations**

| Population | Treatments | GR_50_ (g ai. ha^-1^) (SE)^a^ | RI* |
| --- | --- | --- | --- |
| S | Penoxsulam | 2.46 (0.17) | - |
|  | Malathion+ Penoxsulam | 2.52 (0.21) | 1.02 |
| R | penoxsulam | 25.27 (2.31) | 10.27 |
|  | Malathion+ Penoxsulam | 17.12 (3.42) | 6.96 |

*RI, resistance index.

**Supplemental Table 2: RNA-seq analysis of candidate transcription factors**

| Gene family | Function annotation | Log_2_FC | *p*Value |
| --- | --- | --- | --- |
| AP2/ERF | Ethylene-responsive transcription factor RAP2-13 | 1.73 | 2.80e-4 |
| bZIP | bZIP transcription factor 3 | 1.60 | 1.29e-05 |
| bZIP | bZIP transcription factor 1 | 1.92 | 3.37e-19 |
| DOF | Cyclic DOF factor 2 | 1.85 | 4.44e-4 |
| GATA | GATA transcription factor 4 | 1.66 | 2.66e-07 |
| MAD | MADS-box transcription factor 47 | 7.09 | 1.70e-77 |
| MYB | Transcription factor MYB3R-2 | 1.69 | 4.18e-09 |
| MYB | Transcription factor MYB 6 | 1.58 | 3.66e-08 |
| NAC | NAC domain-containing protein 2 | 3.46 | 2.26e-37 |
| NAC | NAC domain-containing protein 48 | 2.73 | 9.87e-16 |
| NAC | NAC domain-containing protein 67 | 2.24 | 2.29e-20 |

**Supplemental Table 3: RNA-seq analysis of differentially expressed P450s genes**

| Gene family | Function annotation | Log_2_FC | *p*Value |
| --- | --- | --- | --- |
| Cytochrome P450 | cytochrome P450 | 3.90 | 1.01e -102 |
| Cytochrome P450 | cytochrome P450 88A1 | 2.62 | 9.25e -20 |
| Cytochrome P450 | cytochrome P450 71A1 | 4.82 | 9.18e -39 |
| Cytochrome P450 | cytochrome P450 88A1-like | 1.45 | 0.000279187 |
| Cytochrome P450 | Cytochrome P450 711A1 | 1.20 | 0.000555506 |
| Cytochrome P450 | cytochrome P450 709B2-like | 2.35 | 1.01e -07 |
| Cytochrome P450 | cytochrome P450 | 4.33 | 6.63e -62 |
| Cytochrome P450 | cytochrome P450 78A5-like | 1.88 | 0.000367011 |
| Cytochrome P450 | cytochrome P450 81D1-like | 1.34 | 0.000633307 |
| Cytochrome P450 | Cytochrome P450 76M5 | 1.80 | 9.87e -16 |
| Cytochrome P450 | cytochrome P450 89A2 | 4.63 | 3.48e -78 |
| Cytochrome P450 | cytochrome P450 81A21 | 5.12 | 5.14e -129 |
| Cytochrome P450 | Cytochrome P450 711A1 | 1.67 | 4.60e -09 |
| Cytochrome P450 | cytochrome P450 724B1 | 1.28 | 3.71e -07 |
| Cytochrome P450 | cytochrome P450 89A2-like | 3.40 | 1.47e -24 |
| Cytochrome P450 | cytochrome P450 704A371 | 1.71 | 2.25e -21 |
| Cytochrome P450 | cytochrome P450 71A1 | 1.25 | 0.000527506 |
| Cytochrome P450 | cytochrome P450 97B2 | 1.02 | 6.06e -05 |
| Cytochrome P450 | Cytochrome P450 90B1 | 1.31 | 0.000331985 |
| Cytochrome P450 | cytochrome P450 | 1.78 | 3.34e -19 |
| Cytochrome P450 | cytochrome P450 71A1 | 4.39 | 2.28e -20 |
| Cytochrome P450 | cytochrome P450 | 3.04 | 5.89e -15 |
| Cytochrome P450 | Cytochrome P450 78A9 | 1.18 | 1.33e -08 |
| Cytochrome P450 | Cytochrome P450 71A1 | 1.39 | 6.85e -08 |
| Cytochrome P450 | cytochrome P450, partial | 1.14 | 1.62e -11 |
| Cytochrome P450 | cytochrome P450 78A5-like | 2.37 | 1.55e -06 |
| Cytochrome P450 | cytochrome P450 | 1.34 | 2.28e -06 |

**Supplemental Table 4: Number of total clean sequencing reads and mapping rate for each replicate of DAP-seq**

| Samples | Total reads | Reads mapped | Reads mapped and paired | Reads unpaired | Map ratio |
| --- | --- | --- | --- | --- | --- |
| DOF_1 | 85188342 | 84330012 | 84271100 | 58912 | 0.989924 |
| DOF_2 | 57323068 | 56620467 | 56576266 | 44201 | 0.987743 |
| DOF_input | 79546656 | 78834082 | 78786822 | 47260 | 0.991042 |

**Supplemental Table 5: Summary statistics for RNA-Seq sample quality control**

| Sample ID | Clean reads | Clean bases | GC(%) | N(%) | Q20(%) |
| --- | --- | --- | --- | --- | --- |
| *EcDOF2*-OE#5-1 | 20,446,722 | 6,119,850,901 | 52.16 | 0.01 | 97.96 |
| *EcDOF2*-OE #5-2 | 20,758,040 | 6,213,786,875 | 52.22 | 0.01 | 98.09 |
| *EcDOF2*-OE #5-3 | 21,738,251 | 6,505,782,063 | 52.26 | 0.01 | 97.96 |
| *EcDOF2*-OE #7-1 | 21,263,914 | 6,365,773,330 | 53.37 | 0.01 | 97.93 |
| *EcDOF2*-OE #7-2 | 23,025,746 | 6,891,348,231 | 53.43 | 0.01 | 97.95 |
| *EcDOF2*-OE #7-3 | 20,069,299 | 6,006,099,477 | 53.72 | 0.01 | 97.94 |
| WT-1 | 20,319,323 | 6,083,028,855 | 53.7 | 0.01 | 97.91 |
| WT-2 | 21,321,454 | 6,381,964,950 | 53.76 | 0.01 | 98.13 |
| WT-3 | 19,993,324 | 5,985,185,558 | 53.2 | 0.01 | 97.93 |

**Supplemental Table 6: Summary statistics for RNA-seq sample quality control**

| Sample | Clean Reads Pairs | Clean base(bp) | Q20% | Q30% | GC content% |
| --- | --- | --- | --- | --- | --- |
| WT-1 | 34,847,987 | 10,454,396,100 | 99.19 | 97.39 | 51.84 |
| WT-2 | 27,692,585 | 8,307,775,500 | 99.21 | 97.47 | 53.67 |
| WT-3 | 29,680,311 | 8,904,093,300 | 99.19 | 97.41 | 53.52 |
| osdof-1 | 27,516,120 | 8,254,836,000 | 99.37 | 97.91 | 48.68 |
| osdof-2 | 28,446,240 | 8,533,872,000 | 99.12 | 97.43 | 47.24 |
| osdof-3 | 28,672,031 | 8,601,609,300 | 99.18 | 97.40 | 54.08 |

**Supplemental Table 7: Probes used for EMSA experiments**

| Probes name | Forward | Reverse |
| --- | --- | --- |
| EcCYP81A21 | AACAAACAAAAAGTGCTACAGTGCGCTAC | GTAGCGCACTGTAGCACTTTTTGTTTGTT |
| Mutant-EcCYP81A21 | AACAACCCCCCCCGCGGACAGTGCGCTAC | GTAGCGCACTGTCCGCGGGGGGGGTTGTT |
| EcCYP704A371 | ATACTGGAACCAAAAGTGACATGCCCTAT | ATAGGGCATGTCACTTTTGGTTCCAGTAT |
| Mutant- EcCYP704A371 | ATACTGGAACTCGGTTACGCATGCCCTAT | ATAGGGCATGCGTAACCGAGTTCCAGTAT |
| OsCYP81A6 | GTCTGTTTATGAAAAGGTGGGGTAAGTCC | GGACTTACCCCACCTTTTCATAAACAGAC |
| Mutant- OsCYP81A6 | GTCTGTTTACTCCCCCCACCGGTAAGTCC | GGACTTACCGGTGGGGGGAGTAAACAGAC |
