# Supplementary material for: Herbicide Metabolic Resistance in Poaceae Plants via the GA‐GID1/DELLA‐DOF2‐P450s Module
Source: Adv Sci (Weinh). 2026 Jul 24;13(57):e76742. doi: 10.1002/advs.76742 (PMC13398137; doi:10.1002/advs.76742)
Supplement: Supplementary file 3 [file ADVS-13-e76742-s001.zip › Supplemental Data S1.docx]

>EcDof2

MSDQKDPGIKLFGRVIPLAPDPAPGTMETEEPPCDDQPHEELQPRAAAAAEKDQHNEKEEKEDSGMKVDTPQEKGNEMKADTTQEEGNEMKVDTPQEKGDEMKFDAPQKEQDDEMKVDAQQEKRDEQMKIDAPPMTENIQPGSLPSLDSDHKKEDQGQTNSTEDKAASDPKGENEKPSNDESGQDKTLKKPDKILPCPRCNSMDTKFCYYNNYNVNQPRHFCKNCQRYWTAGGTMRNVPVGAGRRKSKNASLHYRQLLMAPDCMLGSRVDMSKSVLPEALVSPAPIQPTSRNETVLKFGPEVPLCESMVSALNIDEQNVKNPGSATGGENGEDNSCASSVTSYNGLPENAVHADKNGAPIYCNGVGPVPQYYLGTPFMYPWSVGWNNLPVMVPGKGMPESASASEGCSTSSAPWMTSPMMPSSRLPGPAFLYPLVPPALWGCLSGWPATTWNIPWISANGCVSPSSSSNSSCSGNGSPTLGKHSRDSNPLNEEKKEKSLWVPKTLRIDDPDEAAKSSIWATLGIKPGDPGTFKPFQSKVESKGQKSDAAQVLKANPAALSRSQSFQESS

>AtDof1

MAFPSNWSQPTNSNHQHHLQHQLNENGSIISGHGLVLSHQLPPLQANPNPNHHHVATSAGLPSRMGGSMAERARQANIPPLAGPLKCPRCDSSNTKFCYYNNYNLTQPRHFCKGCRRYWTQGGALRNVPVGGGCRRNNKKGKNGNLKSSSSSSKQSSSVNAQSPSSGQLRTNHQFPFSPTLYNLTQLGGIGLNLAATNGNNQAHQIGSSLMMSDLGFLHGRNTSTPMTGNIHENNNNNNNENNLMASVGSLSPFALFDPTTGLYAFQNDGNIGNNVGISGSSTSMVDSRVYQTPPVKMEEQPNLANLSRPVSGLTSPGNQTNQYFWPGSDFSGPSNDLL

>AtDof2

MLPYIGHNSYQQHQFPLPEMEIPEKWKLSYEQEAITAPACPRCASSNTKFCYYNNYSLSQPRYFCKGCRRYWTKGGSLRNIPVGGGCRKRSRSRQNSHKRFGRNENRPDGLINQDDGFQSSPPGSDIDLAAVFAQYVTDRSPSSTDNTTGSDQDSPITTTTHALESLSWDICQETDVDLGFYGEFNNLTQKTKEDQEVFGQFLQEDREEIFEFQGLLDDKEIQEILECSFSEEPDQLVSQGSFMINGDNWSSTDLTRFGI

>AtDof3

MWLSHLFMSLSKLTCNFSIFSVFMACGSIGMSQVRDTPVKLFGWTITPVSHDPYSSSSHVLPDSSSSSSSSSLSLRPHMMNNQSVTDNTSLKLSSNLNNESKETSENSDDQHSEITTITSEEEKTTELKKPDKILPCPRCNSADTKFCYYNNYNVNQPRHFCRKCQRYWTAGGSMRIVPVGSGRRKNKGWVSSDQYLHITSEDTDNYNSSSTKILSFESSDSLVTERPKHQSNEVKINAEPVSQEPNNFQGLLPPQASPVSPPWPYQYPPNPSFYHMPVYWGCAIPVWSTLDTSTCLGKRTRDETSHETVKESKNAFERTSLLLESQSIKNETSMATNNHVWYPVPMTREKTQEFSFFSNGAETKSSNNRFVPETYLNLQANPAAMARSMNFRESI

>AtDof4

MPIISSPNTNPLASMQSKNMIVASSHQQQQQQQPQQPQPQLKCPRCDSSNTKFCYYNNYSLSQPRHFCKACKRYWTRGGTLRNVPVGGSYRKNKRVKRPSTATTTTASTVSTTNSSSPNNPHQISHFSSMNHHPLFYGLSDHMSSCNNNLPMIPSRFSDSSKTCSSSGLESEFLSSGFSSLSALGLGLPHQMSHDHTINGSFINNSTTNKPFLLSGLFGSSMSSSSTLLQHPHKPMNNGGDMLGQSHLQTLASLQDLHVGGNNEDMKYKEGKLDQISGNINGFMSSSSSLDPSNYNNMWNNASVVNGAWLDPTNNNVGSSLTSLI

>AtDof5

MATQDSQGIKLFGKTITFNANITQTIKKEEQQQQQQPELQATTAVRSPSSDLTAEKRPDKIIPCPRCKSMETKFCYFNNYNVNQPRHFCKGCQRYWTAGGALRNVPVGAGRRKSKPPGRVGGFAELLGAATGAVDQVELDALLVEEWRAATASHGGFRHDFPVKRLRCYTDGQSC

>AtDof6

MPSEPNQTRPTRVQPSTAAYPPPNLAEPLPCPRCNSTTTKFCYYNNYNLAQPRYYCKSCRRYWTQGGTLRDVPVGGGTRRSSSKRHRSFSTTATSSSSSSSVITTTTQEPATTEASQTKVTNLISGHGSFASLLGLGSGNGGLDYGFGYGYGLEEMSIGYLGDSSVGEIPVVDGCGGDTWQIGEIEGKSGGDSLIWPGLE

ISMQTNDVK

>AtDof7

MQDLTSAAAYYHQSMMMTTAKQNQPELPEQEQLKCPRCDSPNTKFCYYNNYNLSQPRHFCKNCRRYWTKGGALRNIPVGGGTRKSNKRSGSSPSSNLKNQTVAEKPDHHGSGSEEKEERVSGQEMNPTRMLYGLPVGDPNGASFSSLLASNMQMGGLVYESGSRWLPGMDLGLGSVRRSDDTWTDLAMNRMEKN

>AtDof8

MDTAKWPQEFVVKPMNEIVTNTCLKQQSNPPSPATPVERKARPEKDQALNCPRCNSLNTKFCYYNNYSLTQPRYFCKDCRRYWTAGGSLRNIPVGGGVRKNKRSSSNSSSSSPSSSSSSKKPLFANNNTPTPPLPHLNPKIGEAAATKVQDLTFSQGFGNAHEVKDLNLAFSQGFGIGHNHHSSIPEFLQVVPSSSMKNNPLVSTSSSLELLGISSSSASSNSRPAFMSYPNVHDSSVYTASGFGLSYPQFQEFMRPALGFSLDGGDPLRQEEGSSGTNNGRPLLPFESLLKLPVSSSSTNSGGNGNLKENNDEHSDHEHEKEEGEADQSVGFWSGMLSAGASAAASGGSWQ

>AtDof9

MSKSRDTEIKLFGRTITSLLDVNCYDPSSLSPVHDVSSDPSKEDSSSSSSSCSPTIGPIRVPVKKSEQESNKFKDPYILSDLNEPPKAVSEISSPRSSKNNCDQQSEITTTTTTSTTSGEKSTALKKPDKLIPCPRCESANTKFCYYNNYNVNQPRYFCRNCQRYWTAGGSMRNVPVGSGRRKNKGWPSSNHYLQVTSEDCDNNNSGTILSFGSSESSVTETGKHQSGDTAKISADSVSQENKSYQGFLPPQVMLPNNSSPWPYQWSPTGPNASFYPVPFYWGCTVPIYPTSETSSCLGKRSRDQTEGRINDTNTTITTTRARLVSESLRMNIEASKSAVWSKLPTKPEKKTQGFSLFNGFDTKGNSNRSSLVSETSHSLQANPAAMSRAMNFRESMQQ

>AtDof10

MDPEQEISNETLETILVSSTKGSNNNNKKMEEEMKKKVSRGELGGEAQNCPRCESPNTKFCYYNNYSLSQPRYFCKSCRRYWTKGGTLRNVPVGGGCRRNKRSSSSAFSKNNNNKSINFHTDPLQNPLITGMPPSSFGYDHSIDLNLAFATLQKHHLSSQATTPSFGFGGDLSIYGNSTNDVGIFGGQNGTYNNSLCYGFMSGNGNNNQNEIKMASTLGMSLEGNERKQENVNNNNNNSENPSKVFWGFPWQMTGDSAGVVPEIDPGRESWNGMVSSWNNGLLNTPLV

>AtDof11

MVFSSVSSFLDPPINWPQSANPNNHPHHHQLQENGSLVSGHHQVLSHHFPQNPNPNHHHVETAAATTVDPSSLNGQAAERARLAKNSQPPEGALKCPRCDSANTKFCYFNNYNLTQPRHFCKACRRYWTRGGALRNVPVGGGCRRNKKGKSGNSKSSSSSQNKQSTSMVNATSPTNTSNVQLQTNSQFPFLPTLQNLTQLGGIGLNLAAINGNNGGNGNTSSSFLNDLGFFHGGNTSGPVMGNNNENNLMTSLGSSSHFALFDRTMGLYNFPNEVNMGLSSIGATRVSQTAQVKMEDNHLGNISRPVSGLTSPGNQSNQYWTGQGLPGSSSNDHHHQHLM

>AtDof12

MATQDSQGIKLFGKTIAFNTRTIKNEEETHPPEQEATIAVRSSSSSDLTAEKRPDKIIACPRCKSMETKFCYFNNYNVNQPRHFCKGCHRYWTAGGALRNVPVGAGRRKSKPPGRVVVGMLGDGNGVRQVELINGLLVEEWQHAAAAAHGSFRHDFPMKRLRCYSDGQSC

>AtDof13

MVFSSIQAYLDSSNWQQAPPSNYNHDGTGASANGGHVLRPQLQPQQQPQQQPHPNGSGGGGGGGGGSIRAGSMVDRARQANVALPEAALKCPRCESTNTKFCYFNNYSLTQPRHFCKTCRRYWTRGGALRNVPVGGGCRRNRRTKSNSNNNNNSTATSNNTSFSSGNASTISTILSSHYGGNQESILSQILSPARLMNPTYNHLGDLTSNTKTDNNMSLLNYGGLSQDLRSIHMGASGGSLMSCVDEWRSASYHQQSSMGGGNLEDSSNPNPSANGFYSFESPRITSASISSALASQFSSVKVEDNPYKWVNVNGNCSSWNDLSAFGSSR

>AtDof14

MDATKWTQGFQEMMNVKPMEQIMIPNNNTHQPNTTSNARPNTILTSNGVSTAGATVSGVSNNNNNTAVVAERKARPQEKLNCPRCNSTNTKFCYYNNYSLTQPRYFCKGCRRYWTEGGSLRNVPVGGSSRKNKRSSSSSSSNILQTIPSSLPDLNPPILFSNQIHNKSKGSSQDLNLLSFPVMQDQHHHHVHMSQFLQMPKMEGNGNITHQQQPSSSSSVYGSSSSPVSALELLRTGVNVSSRSGINSSFMPSGSMMDSNTVLYTSSGFPTMVDYKPSNLSFSTDHQGLGHNSNNRSEALHSDHHQQGRVLFPFGDQMKELSSSITQEVDHDDNQQQKSHGNNNNNNNSSPNNGYWSGMFSTTGGGSSW

>AtDof15

MQDPAAYYQTMMAKQQQQQQPQFAEQEQLKCPRCDSPNTKFCYYNNYNLSQPRHFCKSCRRYWTKGGALRNVPVGGGSRKNATKRSTSSSSSASSPSNSSQNKKTKNPDPDPDPRNSQKPDLDPTRMLYGFPIGDQDVKGMEIGGSFSSLLANNMQLGLGGGGIMLDGSGWDHPGMGLGLRRTEPGNNNNNPWTDLAMNR

AEKN

>AtDof16

MDYSSMHQNVMGVSSCSTQDYQNQKKPLSATRPAPPEQSLRCPRCDSTNTKFCYYNNYSLSQPRYFCKSCRRYWTKGGILRNIPIGGAYRKHKRSSSATKSLRTTPEPTMTHDGKSFPTASFGYNNNNISNEQMELGLAYALLNKQPLGVSSHLGFGSSQSPMAMDGVYGTTSHQMENTGYAFGNGGGGMEQMATSDPNRVLWGFPWQMNMGGGSGHGHGHVDQIDSGREIWSSTVNYINTGALL

>AtDof17

MMMETRDPAIKLFGMKIPFPSVFESAVTVEDDEEDDWSGGDDKSPEKVTPELSDKNNNNCNDNSFNNSKPETLDKEEATSTDQIESSDTPEDNQQTTPDGKTLKKPTKILPCPRCKSMETKFCYYNNYNINQPRHFCKACQRYWTAGGTMRNVPVGAGRRKNKSSSSHYRHITISEALEAARLDPGLQANTRVLSFGLEAQQQHVAAPMTPVMKLQEDQKVSNGARNRFHGLADQRLVARVENGDDCSSGSSVTTSNNHSVDESRAQSGSVVEAQMNNNNNNNMNGYACIPGVPWPYTWNPAMPPPGFYPPPGYPMPFYPYWTIPMLPPHQSSSPISQKCSNTNSPTLGKHPRDEGSSKKDNETERKQKAGCVLVPKTLRIDDPNEAAKSSIWTTLGIKNEAMCKAGGMFKGFDHKTKMYNNDKAENSPVLSANPAALSRSHNFHEQI

>AtDof18

MPTSDSGEPRRIAMKPNGVTVPISDQQEQLPCPRCDSSNTKFCYYNNYNFSQPRHFCKACRRYWTHGGTLRDVPVGGGTRKSAKRSRTCSNSSSSSVSGVVSNSNGVPLQTTPVLFPQSSISNGVTHTVTESDGKGSALSLCGSFTSTLLNHNAAATATHGSGSVIGIGGFGIGLGSGFDDVSFGLGRAMWPFSTVGTATTTNVGSNGGHHAVPMPATWQFEGLESNAGGGFVSGEYFAWPDLSITTPGNSLK

>AtDof19

MERAEALTSSFIWRPNANANAEITPSCPRCGSSNTKFCYYNNYSLTQPRYFCKGCRRYWTKGGSLRNVPVGGGCRKSRRPKSSSGNNTKTSLTANSGNPGGGSPSIDLALVYANFLNPKPDESILQENCDLATTDFLVDNPTGTSMDPSWSMDINDGHHDHYINPVEHIVEECGYNGLPPFPGEELLSLDTNGVWSDALLIGHNHVDVGVTPVQAVHEPVVHFADESNDSTNLLFGSWSPFDFTADG

>AtDof20

MVFSSLPVNQFDSQNWQQMISILVFFSTSRLFKKLFLVDKNLFSCLLQGLMYNVFLTGLIFSLQGNQHQLECVTTDQNPNNYLRQLSSPPTSQVAGSSQARVNSMVERARIAKVPLPEAALNCPRCDSTNTKFCYFNNYSLTQPRHFCKTCRRYWTRGGSLRNVPVGGGFRRNKRSKSRSKSTVVVSTDNTTSTSSLTSRPSYSNPSKFHSYGQIPEFNSNLPILPPLQSLGDYNSSNTGLDFGGTQISNMISGMSSSGGILDAWRIPPSQQAQQFPFLINTTGLVQSSNALYPLLEGGVSATQTRNVKAEENDQDRGRDGDGVNNLSRNFLGNININSGRNEEYTSWGGNSSWTGFTSNNSTGHLSF

>AtDof21

MDATKWTQGFQEMINVKPMEQMISSTNNNTPQQQPTFIATNTRPNATASNGGSGGNTNNTATMETRKARPQEKVNCPRCNSTNTKFCYYNNYSLTQPRYFCKGCRRYWTEGGSLRNVPVGGSSRKNKRSSTPLASPSNPKLPDLNPPILFSSQIPNKSNKDLNLLSFPVMQDHHHHGMSHFFHMPKIENNNTSSSIYASSSPVSALELLRSNGVSSRGMNTFLPGQMMDSNSVLYSSLGFPTMPDYKQSNNNLSFSIDHHQGIGHNTINSNQRAQDNNDDMNGASRVLFPFSDMKELSSTTQEKSHGNNTYWNGMFSNTGGSSW

>AtDof22

MDHHQYHHHDQYQHQMMTSTNNNSYNTIVTTQPPPTTTTMDSTTATTMIMDDEKKLMTTMSTRPQEPRNCPRCNSSNTKFCYYNNYSLAQPRYLCKSCRRYWTEGGSLRNVPVGGGSRKNKKLPFPNSSTSSSTKNLPDLNPPFVFTSSASSSNPSKTHQNNNDLSLSFSSPMQDKRAQGHYGHFSEQVVTGGQNCLFQAPMGMIQFRQEYDHEHPKKNLGFSLDRNEEEIGNHDNFVVNEEGSKMMYPYGDHEDRQQHHHVRHDDGNKKREGGSSNELWSGIILGGDSGGPTW

>AtDof23

MNNLNVFTNEDNEMNVMPPPRVCPRCYSDQTRFSYFNNNKKSQPRYKCKNCCRCWTHGGVLRNIPVTGICDKSNLPKIDQSSVSQMILAEIQQGNHQPFKKFQENISVSVSSSSDVSIVGNHFDDLSELHGITNSTPIRSFTMDRLDFGEESFQQDLYDVGSNDLIGNPLINQSIGGYVDNHKDEHKLQFEYES

>AtDof24

MDNFNVVANEDNQVNDVKPPPPPPRVCARCDSDNTKFCYYNNYSEFQPRYFCKNCRRYWTHGGALRNVPIGGSSRAKRTRINQPSVAQMVSVGIQPGSHKPFFNVQENNDFVGSFGASSSSFVAAVGNRFSSLSHIHGGMVTNVHPTQTFRPNHRLAFHNGSFEQDYYDVGSDNLLVNQQVGGYVDNHNGYHMNQVDQYNWNQSFNNAMNMNYNNASTSGRMHPSHLEKGGP

>AtDof25

MDNLNVFANEDNQVNGLKRPPPSRVCPRCDSDNTKFCFYNNYSESQPRYFCKNCRRYWTHGGALRNIPVGGSCRKPKRLKVDQSSISEMVSVENQPINHQSFRQTQENNEFVRSFDASSSATVTAVPNHFGYLSELHGVTNLLPIQSFRTMDCLDFGDESFQQGYYDVGSNDLIDNPLINQSIGGYVDNLTSYCINQVEPKLQPRYEHES

>AtDof26

MDNLNVFANEDNQVNDVKPPPPPPRVCARCDSDNTKFCYYNNYCEFQPRYFCKNCRRYWTHGGALRNIPIGGSSRAKRARVNQPSVARMVSVETQRGNNQPFSNVQENVHLVGSFGASSSSSVGAVGNLFGSLYDIHGGMVTNLHPTRTVRPNHRLAFHDGSFEQDYYDVGSDNLLVNQQVGGYGYHMNPVDQFKWNQSFNNTMNMNYNNDSTSGSSRGSDMNVNHDNKKIRYRNSVIMHPCHLEKDGP

>AtDof27

MDTAQWPQEIVVKPLEEIVTNTCPKPQPQPLQPQQPPSVGGERKARPEKDQAVNCPRCNSTNTKFCYYNNYSLTQPRYFCKGCRRYWTEGGSLRNIPVGGGSRKNKRSHSSSSDISNNHSDSTQPATKKHLSDHHHHLMSMSQQGLTGQNPKFLETTQQDLNLGFSPHGMIRTNFTDLIHNIGNNTNKSNNNNNPLIVSSCSAMATSSLDLIRNNSNNGNSSNSSFMGFPVHNQDPASGGFSMQDHYKPCNTNTTLLGFSLDHHHNNGFHGGFQGGEEGGEGGDDVNGRHLFPFEDLKLPVSSSSATINVDINEHQKRGSGSDAAATSGGYWTGMLSGGSWC

>AtDof28

MMTSSHQSNTTGFKPRRIKTTAKPPRQINNKEPSPATQPVLKCPRCDSVNTKFCYYNNYSLSQPRHYCKNCRRYWTRGGALRNVPIGGSTRNKNKPCSLQVISSPPLFSNGTSSASRELVRNHPSTAMMMMSSGGFSGYMFPLDPNFNLASSSIESLSSFNQDLHQKLQQQRLVTSMFLQDSLPVNEKTVMFQNVELIPPSTVTTDWVFDRFATGGGATSGNHEDNDDGEGNLGNWFHNANNNALL

>AtDof29

MVFSSFPTYPDHSSNWQQQHQPITTTVGFTGNNINQQFLPHHPLPPQQQQTPPQLHHNNGNGGVAVPGGPGGLIRPGSMAERARLANIPLPETALKCPRCDSTNTKFCYFNNYSLTQPRHFCKACRRYWTRGGALRSVPVGGGCRRNKRTKNSSGGGGGSTSSGNSKSQDSATSNDQYHHRAMANNQMGPPSSSSSLSSLLSSYNAGLIPGHDHNSNNNNILGLGSSLPPLKLMPPLDFTDNFTLQYGAVSAPSYHIGGGSSGGAAALLNGFDQWRFPATNQLPLGGLDPFDQQHQMEQQNPGYGLVTGSGQYRPKNIFHNLISSSSSASSAMVTATASQLASVKMEDSNNQLNLSRQLFGDEQQLWNIHGAAAASTAAATSSWSEVSNNFSSSSTSNI

>AtDof30

MADPAIKLFGKTIPLPELGVVDSSSSYTGFLTETQIPVRLSDSCTGDDDDEEMGDSGLGREEGDDVGDGGGESETDKKEEKDSECQEESLRNESNDVTTTTSGITEKTETTKAAKTNEESGGTACSQEGKLKKPDKILPCPRCNSMETKFCYYNNYNVNQPRHFCKKCQRYWTAGGTMRNVPVGAGRRKNKSPASHYNRHVSITSAEAMQKVARTDLQHPNGANLLTFGSDSVLCESMASGLNLVEKSLLKTQTVLQEPNEGLKITVPLNQTNEEAGTVSPLPKVPCFPGPPPTWPYAWNGVSWTILPFYPPPAYWSCPGVSPGAWNSFTWMPQPNSPSGSNPNSPTLGKHSRDENAAEPGTAFDETESLGREKSKPERCLWVPKTLRIDDPEEAAKSSIWETLGIKKDENADTFGAFRSSTKEKSSLSEGRLPGRRPELQANPAALSRSANFHESS

>AtDof31

MDHLLQHQDVFGNYNKAREAMGLSYSSNPTPLDNDQKKPSPATAVTRPQPPELALRCPRCDSTNTKFCYYNNYSLTQPRYFCKSCRRYWTKGGTLRNIPVGGGCRKNKRSTSSAARSLRTTPEPASHDGKVFSAAGFNGYSNNEHIDLSLAFALLNKQHPGSSSQLGFHSELGSSHQSDMEGMFGTSQQKENATYAFGNGSSGLGDPSRVLWGFPWQMNGESFGMMNIGGGGGHVDQIDSGREMWTNMNYINSGALM

>AtDof32

MQDIHDFSMNGVGGGGGGGGRFFGGGIGGGGGGDRRMRAHQNNILNHHQSLKCPRCNSLNTKFCYYNNYNLSQPRHFCKNCRRYWTKGGVLRNVPVGGGCRKAKRSKTKQVPSSSSADKPTTTQDDHHVEEKSSTGSHSSSESSSLTASNSTTVAAVSVTAAAEVASSVIPGFDMPNMKIYGNGIEWSTLLGQGSSAGGVFSEIGGFPAVSAIETTPFGFGGKFVNQDDHLKLEGETVQQQQFGDRTAQVEFQGRSSDPNMGFEPLDWGSGGGDQTLFDLTSTVDHAYWSQSQWTSSDQDQSGLYLP

>AtDof33

MLETKDPAIKLFGMKIPFPTVLEVADEEEEKNQNKTLTDQSEKDKTLKKPTKILPCPRCNSMETKFCYYNNYNVNQPRHFCKACQRYWTSGGTMRSVPIGAGRRKNKNNSPTSHYHHVTISETNGPVLSFSLGDDQKVSSNRFGNQKLVARIENNDERSNNNTSNGLNCFPGVSWPYTWNPAFYPVYPYWSMPVLSSPVSSSPTSTLGKHSRDEDETVKQKQRNGSVLVPKTLRIDDPNEAAKSSIWTTLGIKNEVMFNGFGSKKEVKLSNKEETETSLVLCANPAALSRSINFHEQM

>AtDof34

MGLTSLQVCMDSDWLQESESSGGSMLDSSTNSPSAADILAACSTRPQASAVAVAAAALMDGGRRLRPPHDHPQKCPRCESTHTKFCYYNNYSLSQPRYFCKTCRRYWTKGGTLRNIPVGGGCRKNKKPSSSNSSSSTSSGKKPSNIVTANTSDLMALAHSHQNYQHSPLGFSHFGGMMGSYSTPEHGNVGFLESKYGGLLSQSPRPIDFLDSKFDLMGVNNDNLVMVNHGSNGDHHHHHNHHMGLNHGVGLNNNNNNGGFNGISTGGNGNGGGLMDISTCQRLMLSNYDHHHYNHQEDHQRVATIMDVKPNPKLLSLDWQQDQCYSNGGGSGGAGKSDGGGYGNGGYINGLGSSWNGLMNGYGTSTKTNSLV

>AtDof35

MSSHTNLPSPKPVPKPDHRISGTSQTKKPPSSSVAQDQQNLKCPRCNSPNTKFCYYNNYSLSQPRHFCKSCRRYWTRGGALRNVPIGGGCRKTKKSIKPNSSMNTLPSSSSSQRFFSSIMEDSSKFFPPPTTMDFQLAGLSLNKMNDLQLLNNQEVLDLRPMMSSGRENTPVDVGSGLSLMGFGDFNNNHSPTGFTTAGASDGNLASSIETLSCLNQDLHWRLQQQRMAMLFGNSKEETVVVERPQPILYRNLEIVNSSSPSSPTKKGDNQTEWYFGNNSDNEGVISNNANTGGGGSEWNNGIQAWTDLNHYNALP

>AtDof36

MPSEFSESRRVPKIPHGQGGSVAIPTDQQEQLSCPRCESTNTKFCYYNNYNFSQPRHFCKSCRRYWTHGGTLRDIPVGGVSRKSSKRSRTYSSAATTSVVGSRNFPLQATPVLFPQSSSNGGITTAKGSASSFYGGFSSLINYNAAVSRNGPGGGFNGPDAFGLGLGHGSYYEDVRYGQGITVWPFSSGATDAATTTSHIAQIPATWQFEGQESKVGFVSGDYVA

>OsDof1

MDDLAAASPPHPPPPPPESHVPPPPQTPEKDSCEDTGDMRISEEKPCTDQELDADQMNSSSFNSSSECENQTPSNDEMTGSESKSEAAQTEGGGSSEEKVLKKPDKILPCPRCNSMDTKFCYYNNYNINQPRHFCKSCQRYWTAGGSMRNLPVGAGRRKSKSSTANYRSILITGSNLAAPAGDAPLYQLSIKGDQTATAVKFAPDSPLCNSMASVLKIGEQSKNAKPTSTAQPRNGETQTCPASGTTSDSPRNEPVNGAVSGHQNGIVGHSGVPPMHPIPCFPGPPFVYPWSPAWNGIPAMAPPVCTAPAEPANSSDNGSTASVQWSMPPVMPVPGYFPVIPSSVWPFISPWPNGAWSSPWIQPNCSVSASSPTSTSTCSDNGSPVLGKHSRDSKPQGDDKAEKNLWIPKTLRIDDPDEAAKSSIWTTLGIEPGDRSMFRSFQSKPESREQISGAARVLQANPAALSRSQSFQETT

>OsDof2

MLSHVEMAPAAGGFKLFGKVIMQCGVSEGTQDKAQGFVVAREKVEPEEEEEEEQRVPAAATSGQRASIKREAADRDEEQRQGGGDAAGQPTQRRLQDSAEARAAAAAPLPCPRCRSRDTKFCYFNNYNVNQPRHFCKACHRYWTAGGALRNVPVGAGRRKNRPLGPLAVAHHNHHHRAAAGFVLGFPNPSSPTSPSPVYTDRWPVTPDRPF

>OsDof3

MCDKDPGIKLFGRVIPLAPEAEAAAAADGSDQPEAAAAAAAEVEPAAQDEDHHKETEERKYDEMKVDVPQEEEDNEMKVDAPQEKKDNEVTADVPEEKGNDEMRVDASESIESIEPVSRSTLDNKKEDQGQMNNVEEKAASDSKDENEKTANDESGQDKVLKKPDKILPCPRCNSMDTKFCYYNNYNVNQPRHFCKNCQRYWTAGGTMRNVPVGAGRRKSKSSSLHYRHLLMAPDCMMGSRVEISKSMNPEAFASAHSTPIQPIGRNETVLKFGPEVPLCESMASVLNIQEQNGTNAAAVPTGENQEDNSCISSITSHNVLPENAAQVDKNSTPVYCNGVGPVPQYYLGAPYMYPWNIGWNNVPMMVPGTSMPESASQSESCSTSSAPWMNMNSPMMPVASRLSAPPFPYPLVPPALWGCLSSWPATAWNIPWIRTNGGCMSPSSSSNSSCSGNGSPLGKHSRDSSLPLKEDKEEKSLWVPKTLRIDDPDEAAKSSIWATLGIKPGDPGIFKPFQSKGESKGQAASETRPARALKANPAALSRSQSFQETS

>OsDof4

MAALRQGDDPAIKLFGRTIPLLLDPPAAAAAAADEVMPNLGNGVKTNNDLPLVSDKLLIVKGIPFCPNNSKKNDLQGISRPDGRIEIDSMTEDVKTEPDGSVPEKILKKPDKILPCPRCNSMETKFCYFNNYNVHQPRHFCRNCQRYWTAGGAMRNVPVGAGRRRNKHVSKYCQAMMTCNNTVAPGDVSDVVHHQVITHGSSLLPATLKENETPTEFISEVPPCKSSASILDIGEPNDTDLVPLASGDNKEEKSCASSVVVSSCSENLMPDNAIMKEPNNRSGCCNGVALPFPTGPALVLPWSLGWNSVALMPATQCSMQPVLGLKDGIPCPPSWPPQLMVPAPGICTPVVPIPLVPPLWSCFPGWPNGMWNAQCPGGNTTVLPSTAPNKISCSGSSSLVLGKHSREESLQEEEKTRNYLWVPKTLRIDDPAEAAKSSIWATLGIKPDDKGIFKSFQPNVAKNGTAPESPQALQANPAAFSRSQSFQETT

>OsDof5

MVFPSVPAYLDPPNWNNQQGQPPRPANVGGGDAQHLPVGPTAAAAAPGEIGGLPTSSSSASAAAAAAQQARPNSMAERARLARAPQPEPALKCPRCDSTNTKFCYYNNYSLSQPRHFCKTCRRYWTRGGSLRNVPVGGGCRRNKRSGKSSSAAAAGASSSSSKPSSSAARQLPGGGASPMPSAAASTQPGGAAAGAIIPPSGLSSMSHHLPFLGAMHPPGPNLGLTFSAGFQPLGGMHHHVDTADQFPVASGGGATIGASLEQWRVQQQQQQQPQQHQFPFLGGALELPPPPPMYQLGLEATRAAGTGATAAAAFTLGQTSATATTSRQEGSMKLEDSKGLEMSLQRQYMAALRQGDGVWGNNNGGNGGSDGGGNGGGGSWTMNFPGFHSSSGGGGDDGGGVL

>OsDof6

MGECKVGGGGGGGDCLIKLFGKTIPVPEPGACAAGDVDKDLQHSGSSTTEPKTQENTVQDSTSPPPQPEVVDTEDSSADKNSSENQQQQGDTANQKEKLKKPDKILPCPRCSSMDTKFCYYNNYNINQPRHFCKNCQRYWTAGGAMRNVPVGAGRRKSKSVSAASHFLQRVRAALPGDPPLYAPVKTNGTVLSFGSDLSTLDLTEQMKHLKDKFIPTTGIKNTDEMPVGLCAEGLSKTEESNQTNLKEKVSADRSPNVAQHPCMNGGAMWPFGVAPPPAYYTSSIAIPFYPAAAAAVAAYWGCMVPGAWNAPWPPQSQSQSVSSSSAASPVSTMTNCFRLGKHPRDGDEELDSKGNGKVWVPKTVRIDDVDEVARSSIWSLIGIKGDKVGADHGRGCKLAKVFESKDEAKASTHTAISSLPFMQGNPAALTRSVTFQEGS

>OsDof7

MGECRGGGGGGDGLIKLFGKTIPVQPDAKDVQQHSGSSSSSTESDVQETAAVAVADPSPRSEVVDGESPPQPGGEAASHQQQQKEMKLKKPDKILPCPRCSSMDTKFCYFNNYNVNQPRHFCKHCQRYWTAGGAMRNVPVGAGRRKNKNATAAAHFLHRVRACAAAAAMPAAPHDATNATVLSFGGGGGGHDAPPVTLDLADKMTRLGKEGLVAHARNADAAAACSEVSSNRDDEQIGNTVAKPANGLQQHPPPPHHHHHSAMNGGGIWPYYTSGIAIPIYPAAPAYWGCMIPPPGAWSLPWPATVQSQAISSSSPPTSATPSVSSFTLGKHPREGGDHEARDHHGNGKVWVPKTIRIDNADEVARSSIRSLFAFRGGDKADDNNDDDGTGVHKLATTVFEPKRDSKTAKHPAITSLPLLHTNPVALTRSATFQEGS

>OsDof8

MLSSHCESMLAYAAAAGRRAVVVDHHQRRYRPNVEVAPNCPRCESPNTKFCYYNNYSLSQPRYFCKGCRRYWTKGGSLRNVPVGGGCRKNRRGKAVRAMVGETMTARGGGGGGAAAFSHRFHGPVRPDMILEGMAGSTAASAGLGEQPGVAAPDEKPAAADGSTIDLALLYAKFLNHHQPTMAEQGGGAAVPESVDTSSGSSSDRTTSPAAAQPAAAAAYGPGQDGLVGEPISTEEHGAAAMARCAQALGELNFSVDQISCYTSLGLPTTDGGDLILPSTLDQHAKYEPFDSLPEDALSLHDIISGDDDVWCNALGCQGLEAALCRP

>OsDof9

MLPYAPRPPSLLVDRRYKQGAEAAPNCPRCDSPNTKFCYYNNYSLSQPRYFCKGCRRYWTKGGSLRNVPVGGGCRKNRRGKSSSSARSAADAVSSGRDAAFGHRFPGPVRPDMVLEGMVGNPANPGQAMPDVAAAADGSTIDLAMLYAKFLNHPPTDAGLGAVTPESGGHVDEAFDTFSASSDLSPGILAAASAQFDPNQDGFGEWSSPASGNDPTSTATTATTSMLCTDASVQAALGELNFAMDQSCFDSLGLPTDVAGAGSLSSWCSIVPSLSTWEEPKYDSLDSFPDDAMSLHECMIGAPDHDWSVDCQGLEALYMP

>OsDof10

MAGAGGAATAAAGGGGGGGVAAGRSGGGGGGGAAAAAGAGAPDPRAEALRCPRCDSANTKFCYYNNYSLSQPRHFCKACKRYWTRGGTLRNVPVGGGCRKNKRSRSGGAAPGGGVGRGGPGGGAAAAVSSAGGGAAGTSPASSLALPQPGSLPSLSSALGLTGGTSLASLLLGSGGSGGDHLGLFQAMQSVVSDAAAFEMHQQHQSQVDHLLGLGYGAAGAQIQAAKPWLHDGGATGGLLDGFYAPLLSGSIVPGLEELQVKAEATTGDHQQKSSAAAAGEQSWDLPTPSSSNVEASIIASDALMAAAAASMNPAVSAAAASTAPSAQSLLYWGNGGIGAAAAAWPDLANCGSSIATLF

>OsDof11

MMAGAPPMHICMDSDWLKGIVPEEHGMGSSSPSAELIACPRAPMQAAAAAADRRLRPQHDQPLKCPRCESTHTKFCYYNNYSLSQPRYFCKTCRRYWTKGGSLRNVPVGGGCRKNKRAPPKKAAAHAQPAVAVAAALQGRHMETGLHLSFSGMQHHLAPPPPAAATAADPLCSLGLFDWKQYDPVFAGSGGGGSPVAALESAGGSEAQFMGAGMMGIGGGGVAEYHALSALRFAAGLGDHLALPFGAVRAEHDAVEVKPVAAERLLSLEWCGEASRTAAPESSISSLGGLGLWSGMIGGGHHHHGSSAAI

>OsDof12

MQEFQSIPGLAGRLFGGAAAADIRRAQAQQGPASRCGGIPSPEAVKCPRCESTNTKFCYYNNYNLSQPRHFCKSCRRYWTKGGVLRNVPVGGGCRKTKRSGSSSAASSAPSTPTAATDNAKNQRRASASSPRSSSGGSGNTSPTAAAATTPTTPATPSSNTIAVINHATTTTTTTNPFPTDVPPPAPIFADQAAALASLFAPPPPPPLPVFSFAAQAKTEDGIASVLLAGQTTAPTAATVADMTPFTSLDAGIFELGDVPPAAYWNAGSCWTDVPDPNVYLP

>OsDof13

MAPAAGDDAVVPRKGAGGGGTTTPPPPPPAQQQQQQPLPPPPPQEQGLRCPRCDSPNTKFCYYNNYSLSQPRHFCKTCRRYWTKGGALRNVPVGGGCRKNKRSRSAAAASRLSLNLPTVEGIGGAAADPAAAAAARLGFLGGGTTMMMSSSTSPLGGAAAAVADFQQGGAVGMLPLPRLQSPAGGGVGHHQYVPFGEWPSGDIAGGNAVNGGGGHGAVSSTIASSIESLSFINQDLHWKLQQQRLATMFLGPPGSASAAAAAAQANDGGGNGAQATASGHAAGGGGGAFMHMAGSVPSMEAAMPSATSWFMDSCSYGLPSPPPPATAAVAATTSSNLNSGGRSSGGDDNATSNCGSAIPAWGDISTFAMLP

>OsDof14

MQEFHPVPGLAGRLFGGAAAAAAVAAVEEVRCPRCDSSNTKFCYYNNYNLSQPRHFCKACRRYWTKGGLLRNVPVGGGCRKPKRPAPPPSSSFTGGGGGGGGCGHRDSKSARSAGGGGDGSGSTASATATPAAAPASSNTLSAAVSQPSSVDALSPPPAPMFADQATAFASLFAPPPPPPSQALPAFASFTAQPKAEEDVADAPALAATEQHRSSSAASFAAHSISPPFAAARSSDGPAAAAAAAAADWAPPTAVLDAGMFDLAGAIGGDTSYWNAASWTDHDGTIYLP

>OsDof15

MEVAEGRTVAAAAAGGGGLGGGARTEAEGLACPRCESTNTKFCYYNNYNLAQPRHFCKACRRYWTRGGALRNVPVGGGTRNKVAPAPCTGRRKRAAHAAHAAAPPPTTTASSAPLPLMPPAVAYELPFLPPPPPLPLAAVDPDRRLLDLGGSFTSLLAPAQLHNGHFTTGFLLGTMSSAPPPPPPPATSTPSPAPAAHPPVSDSIWAMGWPHLSI

>OsDof16

MDAAHWHQGLGLVKPMEEMLMAANAAAGANPNPAATAPSSVTGGALRGGGGGGAPPVAGGAGAGSTERRARPQKEKALNCPRCNSTNTKFCYYNNYSLQQPRYFCKTCRRYWTEGGSLRNVPVGGGSRKNKRSSSSAASASPASASTANSVVTSASMSMSMASTGGGASKNPKLVHEGAQDLNLAFPHHGGLQAPGEFPAFPSLESSSVCNPGGPMGTNGRGGGALSAMELLRSTGCYMPLQVPMQMPAEYATPGFALGEFRAPPPPPQSSQSLLGFSLDAHGSVGGPSAAGFGSSAGLQGVPESTGRLLFPFEDLKPTVSSGTGGGGASGGGAGVDGGHQFDHGKEQQAGGGGGGPGGHDTPGFWNGMIGGGSGTSW

>OsDof17

MDAAHWHQGLGLVKPMEEMLMGANPNPNGSSNQPPPPPSSAASAQRPIAPPAAGAAAGAGAAGAGAGTERRARPQKEKALNCPRCNSTNTKFCYYNNYSLQQPRYFCKTCRRYWTEGGSLRNVPVGGGSRKNKRSSSSVVPSAAASASTSAAVSGSVPVGLAAKNPKLMHEGAQDLNLAFPHHHGRALQPPEFTAFPSLESSSVCNPGGNLAAANGAGGRGSVGAFSAMELLRSTGCYVPLPQMAPLGMPAEYAAAGFHLGEFRMPPPPQQQQQQQAQTVLGFSLDTHGAGAGGGSGVFGACSAGLQESAAGRLLFPFEDLKPVVSAAAGDANSGGDHQYDHGKNQGGGGGVIGGHEAPGFWNSSMIGNGSSNGGGGGGSW

>OsDof18

MQEQQPETGRRPAQQFATVDLRRPKGYAAAPATPQPGSAATAAAAAGPAATAAAAAAGEGDPCPRCESRDTKFCYYNNYNTSQPRHFCKCCRRYWTKGGTLRNVPVGGGTRKKSSSSSSSSSSSSAAAAAPAAKRQKTSKKRRVTTPEPLAATTPVLTEAAADSAAKTTTEATSEKKTTTSTTTTTPPAPDTTSEITTELVVPAVEEDSFTDLLQPDSAAVTLGLDFSDYPSITKSLADPDLHFEWPPPAFDMASYWPAGAGFADPDPTAVFLNLP

>OsDof19

MPGQVMEAALQQLPASMASGSLLLPPACLQHPLPAAAAASGGVGGSSREQCPRCASHDTKFCYYNNYNTSQPRHFCRACRRYWTLGGSLRNVPIGGSTRKRPRPPVRRPPVHFTAAAAAAAAAAPPHHHHHHHGGPLTPPPATSSSSQQAGLLGSLFALGAAPLLEGRVGVGFDLGLGLPGPGHHHAVAGGGGPAAAVATSSSSSAAAPLLWPTGLLDSSSNNAETWRMAAGGMWPEFTAAAAQNIRLVIDIGDTTIQVPLNGPTVVQNIGRQAAAAVAGDSSAGGVSEKTGGAGGGGGEEWMQEQDGLLCMRGRRCGRRGGCLPRPRDWFAALLAADPAAAAVTRDQAGKAMLYLIVNTCTFATSLAVLPDAVRRRRRLRVEEGHRVADHQHDDGRRAVRRGDVRALRRRRLPAHGPFVGTVVAAVTVVVVRCNLALPFRGGDAGHGCSWVSRL

>OsDof20

MANLPSTAAAAAADASGFKLFGKVIQPDGQRGVEESAAAQAPPHPHPPAPPVMEAAAAAAGTSQTLQAAGGGGGGGGGGGGEPLPCPRCGSRETKFCYFNNYNVRQPRHLCRSCRRYWTAGGALRRVASASPGRRRPRPSAARSAAAAAASASAASPPAAVPAASEGAESVDSRS

>OsDof21

MVFSSLPIFLDPPNWTQMQQQPLQCLIGGGGSDHHHLMPPPSGLAPLPSAAGAADTAASAPAAAAQQQQPRPAVVSMSERARLARVPLPEPGTLRCPRCDSTNTKFCYFNNYSLSQPRHFCKACRRYWTRGGALRNVPVGGGCRRNTKRSTKKSSSSSSRQGGGAGNAAAAATSSSSTTSTSTTATTSSAAAAAAAAAADVIASMQAGGALLPHHLIGGLPSSAAAAAALEASLEGYHHHHHAHGHQLPFLQPPPFLQQGLHGYHFADGDVAAGAALADGGFPRGVASGLLAQLASVKMEEHGTNNGGGVGGGFVGAHEQYWHGGNGGGGWPAEFLSGFSSSSSGNVL

>OsDof22

MTSASLLLAPRSPDMAAAGILPVSGGGGASSARPVSMAERARMAKIPQPEPGLKCPRCDSTNTKFCYFNNYSLTQPRHFCKACRRYWTRGGALRNVPVGGGFRRNKRGTKPSNSKKPAAAVAGGVMAPPHAQLQLPFGFDGGGGGGHGSIIGGGGGGGASRLGFPELSSLHAAAAVDYQLGGGGGGDGLGLERQRLPHFPFLARSNAAVHPPPLMSTAAGVSYPFGDVAAGGLGGDMPANAASVAGSAGLITQMASVKMDDIDNHPPPSAATTTASSPIEFLGLRGSLQFWGGGGGHRGGGDGAGGSAAPGGGGGGWSDLPAFDLSTSGNIL

>OsDof23

MASGGALSPVEEKPTVVKTTKAEQHEEEAAVAVKSAAEMMKKSSPCCPRCNSIKTKFCYYNNYSMAQPRYFCRECRRYWTQGGSLRNVPVGGGCRKSKRSSASSASASAASPPAPAVGAAPPVVPALSSAISKLLQSEPMAAPCADFPNVLPTFVSTGFELPAAAGDRLSLGSFGAFGNLSAAVAAPGGGGGSSTTTSFMDMLRGVGGLFDGVGNSHQMGGNGGGGGSYYAPLITGAGNGMLMPPPPLPPFSGSLMQHGMQGLFANHAMGGGGGGVMNAGEDGSVMAGLGGGQWPPALGGADEQQGGGDGGEAVMTKDTGGGASSSASRPDYFYGWNSAAGGVVAGGGIGGNAAAATGATPWQGLIDSSSAMM

>OsDof24

MIFPPAFLDSSSWNDNNNNNHNQQQQQQHAHGHHQHHQVAAGCGGGGGGGDGNSHELLQQQSMIPGTLADGGGGGGAVGPAKPMSMSERARLARIPLPEPGLKCPRCDSTNTKFCYFNNYSLSQPRHFCRACRRYWTRGGALRNVPVGGGYRRHAKRAKPKPASAAGSASAATTTAGSTPAGSTTTTTTSSTCATPNAPALPAMLGGNLSILPPLLRLADFDAMSLGSTFSGMAAAAGKPPPVDAAGCYSVGAATGLEQWRLQQMQSFPFFHAMDHQAAMAAPPPAMAMPGMFQLGLDGDGHGSGGGEDGGELHHAMPSSKREGYPRGMYGDHHLAGGYTSYSSATTGNHLL

>OsDof25

MAPAVASSPSLVLSAAAATASNKRPADSDASPPHQGDRTGQQEKKQQQLECPRCRSTNTKFCYYNNYSTSQPRHFCRACRRYWTHGGTLRDVPVGGASRRGGGGKRRRVSADADPSSASPPPPTTSTTDAYADLPAGFPFLSDGAFLPQFGLAGVAPAAFSWASAVPDLYNCGIAPWDDGTAVTGAAWDNFADIAGLDLSWPPPGN

>OsDof26

MSSPFLGSSSSSASSPLSYLTPPRPPPPPPPLLMVRHGLARWLTDLDDELMVFDDDLGVQGQGYAAAANGGIGGGGVEAVNAAAAPRQGGRHAGHPPLPRPPPRQCPRCGSANTKFCYYNNYSRTQPRYLCKACRRHWTEGGTLRDVPVGGGRKNSKRAAGGGKAGATASTAASAHVVAPAAAPPTSSSFPDLLRQMLMAPATAGGGGGYSIDLTAWQQMAAFAAPPQAATGDVGGAVGAASTAAPDANCGGGGVQYWNGWLQDDMPGLDGSC

>OsDof27

MEAPLHQSPVPLLPPPPPPPRVVGVQQQQQQEAVVPPPPAMAAAAGGGGREQCPRCASRDTKFCYYNNYNTAQPRHFCRACRRYWTLGGSLRNVPIGGSTRKRPRPSRPARAAVAAAIAAAAAASASGSQIAAQQQQAPPVVMSQHEAAAAAAAASGGGGGDGLLVSLLGAAPVLEGRLGGGIGVDLLGGEQLGFGAMAMPPAPLLWPARVLEGGDAWKSAAAAAGVSYSPFPALWQELAAAAPVELAGGGGLLRHGGGGAPQLM

>OsDof28

MIQELLGGTTMDQLKGASALNHASLPVVLQPIVSNPSPTSSSSTSSRSSAQATQQRSSSATSSPHGQGQGGGAAEQAPLRCPRCNSSNTKFCYYNNYNLTQPRHFCKTCRRYWTKGGALRNVPIGGGCRKPRPMPAPVAKPPMSCKAAPPLGLGGGPVSWASGQQAATAHLMALLNSARGVQGHGGSNVHRLLGLDTMGHLQILPGAPNGAGAGTAASLWPQSAPRPVTPPPPHMDSQLGMGTLGHHDVLSSLGLKLPSSASSSPAASYYSDQLHAVVSNAGRPQAPYDVATASLPCTTAVTSLPSALSSVSAAAPTSNTVGMDLPPVSLAAPEMQYWNGPAAMSVPWPDLPTPNGAFP

>OsDof29

MPPHHGGLMAPRPDMVAAAVAASGGGGGGGGPTGGTAVRPGSMTERARLAKIPQPEPGLKCPRCESTNTKFCYFNNYSLSQPRHFCKTCRRYWTRGGALRNVPVGGGCRRNKRTKSSKSSSSTSAAGSASATGGTSSSTSSTATGGSSSAAAAAAMMPPQAQLPFLASLHHPLGGGDHYSSGASRLGFPGLSSLDPVDYQLGGGAAAAAAIGLEQWRLPQIQQFPFLSRNDAMPPPMSGIYPFDAEAAADAAGFAGQLLAGTKVPGSSGLITQLASVKMEDSNAQSAAMNSSPREFLGLPGNLQFWGGGNGAGPGGNGDGATGGSGAGVAPGGGGSGGGWADLSGFNSSSSGNIL

>OsDof30

MQEAGRRPAPQFAGVDLRRPKGYPAAAQLTPAAEEAAAGVGDPCPRCESRDTKFCYYNNYNTSQPRHFCKSCRRYWTKGGSLRNVPVGGGSRKSSTSSSSAAAAAASSSSSPSSPAKSPKRSKNSKRRRVSPPPPQPVPAPPPPTTADAADVAAPTAPEATTKKAPEDLTAAAATQPAVALGLGVADGGGGGKEHLDTSPFEWPSGCDLGPYWPTGVFADTDPSLFLNLP

>SbDof1

MEAPLHQFPVPPPLPQDALLRQQAVAARALMVAATAGNKAAGREQCPRCASRDTKFCYYNNYNTAQPRHFCRACRRYWTLGGSLRNVPVGGSTRKRPRPRPARHTRAMAAAAFGAAAMATPSTTTTASDGGSPFASPATFQGVGGGGGLLLSSLLLGSVSASSSASPLLALGAAPLLEGRLGFDLGFGDAALGGGGGGGAHAADLPHHHQLPLGGGPPLLPWPAATTRILEGDRAETTTVFPFPPAGAVWQELAATAPVVEAAGLHHGGAPHLLL

>SbDof2

MAECQGGGGGDFLIKLFGKTIPVPESGDAKDLQQSSSSSSSTEQDHQDAHALDQENPHPDSSDPSPQPEVVDAEDPKSSPETTHQKPGQGNGSGDAASQREKLKKPDKVLPCPRCNSMDTKFCYFNNYNVNQPRHFCKNCQRYWTAGGAMRNVPVGAGRRKNKNAVAASHFLHRVGAACGGGGDTLKTTNGTVLSFGGHGGGCVPPGPACLDLVEQLSHHLAAPVIRNAGNNPGPCSEGSSNCRDDNKTINDRSCVDEAAAANGDDGSVQHPASMNNGGATVWPPPYSCAPSPAAYFSSGIAIPIYPAAPGYWGCMVPGAWSLPWPVQQPPSSQSQGPAAGLSSSTSPTTTSAPSVSSSGAADSHTLGLGKHPRDREEGDDGRNAKVWAPKTIRIDDVDEVARSSIWSLIGIKGDKAKQQDDDAAGGHKQKQLVGMVFEPKREATKKPAAMMTSSPLLHANPVALTRSVAFQEGS

>SbDof3

MAERARLARMPQPEPALKCPRCESTNTKFCYYNNYSLSQPRHFCKTCRRYWTRGGSLRNVPVGGGCRRNKRSSKSSSAAAAGSSSSSKTSSSGRLLGGPSATPSTTPGVTGAIITPGLSSFSHHHLPFLGSMHPPGPNLGLAFSAGLPLLGMQHLDTVDQFPVASGGGTTIGASLEQWRVQQQQQRQFPFMTGGILDLPQPPTYQLGLEANRGGGGGSAAAAFTLGQPTTTSATATTGRQEGSPKKMEDSKGQDMSLQRQYMAALRHGSGAHGGWDGNAAGGSGSDGGGTGSAGSTWPMNIIPGFHSSSTTGGNGGGSL

>SbDof4

MAAVVAASGGGSTPGAGGGPTAGGSAIRPGSMTERARLAKIPQPEPGLKCPRCESTNTKFCYFNNYSLSQPRHFCKTCRRYWTRGGALRNVPVGGGCRRNKRTKSSKSNSSSAASASGAGGTSSSTSSTATGGSSAGGASGAGIMPSHQGHGHLPFLASLHHPLAGGDHYSTGASRLGFPGLSSLDPVDYHQFGASAGAGGAALGLEQWRLPQIQHQFPFLSGRPDAVPPTMSGIYPFDVEGHGGDGTGFAGHMLGASKVPGSAGLITQLASVKMEDNPASAAMANSSPREFLGLPGNLQFWGGGSNGGANGNNGGGAGNAGGGGSGGGGGGGGGTVAPGSSWVDLSGFNSSSSGNVL

>SbDof5

MASATTAATGDDAVGTRKGGTGGATAGGGTAPQTQQQTPPPPEQGLRCPRCDSPNTKFCYYNNYSLSQPRHFCKTCRRYWTKGGALRNVPVGGGCRKNKRSRSAAAVAVAAAAAASRLSLNLPPAEAAAADQQQAAARLGFLGAAHHHPAVASSPIGGAGAPAADYHHQQAGMMALQPRLHGPGAVVGQYVPFGDWPSSGDVSSGGGCHAVNGGAAAAVSMSSSIASSIESLSFINQDLHWKLQQQRLATMFLGTPTTSSGAAAGAHVDGAAAAAGAAAPHVVGGTFLHMAAGPPPPHGMVEATTMAPPAATSWFMDSSCYVLPSPTTAHHANTAAAAVVATSNCNVVNSSGGGDDDNATSNNNNCGPGSAIPSWGDMSTFAMLP

>SbDof6

MAGAGGATAVQQPAAGAPAAAGGAARSGVGAGAAGAPVADPRAEALRCPRCDSANTKFCYYNNYSLSQPRHFCKACKRYWTRGGTLRNVPVGGGCRKNKRSRSSGGAGGRNGSSSSASAAAAAVTSSSAASTLSLPPPTGSLPSLTSALGLPGGASLASLLLGTAGSGGDHLGLFQAAMQSVVSSEATAYEMQQQQSQVDHLLGLGYGGATGAGAQIHLKPWMQEAAGAGGIMDSFYAPLLSSSLVPGLEELHVKAEVAGAGDHQQKPAPGDQQSASWELPTPSSSNVDANVIASDALMAAAAASMNPAVSSSTSTAPTTVPSSFMYWGNGGIGGAAAAWPDLANCGSSIATLF

>SbDof7

MQEHGRRPVTPFAGVDLRRPKGYPAPAAVAAAKEAAPARAPVVGDPCPRCGARDTKFCYYNNYNTSQPRHFCKSCRRYWTKGGSLRNVPVGGGTRKSSSSSTSSPSATTSASPGGAAPKNTKRSKNSKRRRVAPAPDPAAPGTDAPTVTATATADVANTAPSTEAAAATVAASEKPVTMTEEEPAAAVVVATETKPPAAPGLGLADAGSGGGGKELLPDPSHFEWPSGCDLGSYWGTSVFADTDPALFLNLP

>SbDof8

MRVTGEKPCTHQELDVDQTNSSSSFNNSSECENLAPSNDEISGSESNLEIAKTEGDVPSGEKVLKKPDKILPCPRCNSMDTKFCYYNNYNIKQPRHFCKSCQRYWTAGGSMRNIPVGAGRRKSKSSSANCRSILIPGSSVATPGGEASLFPLSINGNQAAVSFGPDSPLCNSMASVLKIGGEQIKSSNPASAAQPRNGENQMCPPCTTSSDGPRNESQKETANAHQNGIIGQSNGVTSVHPIPFFPGPPFVYPWSPAWNGIPTMAAAVCPAPAEAANSSENCTTSSNVQWNVPPIVPVLPPGFCGPIPVPVMPPSVWPFITPWPNGAWNAPWLGPSSTVPSSSPTSSSTCSESGSPVLGKHSRDSKPQGDEKAERCLWIPKTLRIDDPVEAAKSSIWTTLGIEPGDRGMFRPFQSKPERQEQISGAARALQANPAALSRSRSFQETT

>SbDof9

MAGQVMEAQAARLQPPTMLAPPFAPLPHSTTCKHDVHHHHLTTTTTTATMAATSGTTTNNVVTRTGGAAAADMAAYLQQLQDAAEAAAKSSGGTGGAARGEQCPRCASHDTKFCYYNNYNTSQPRHFCRACRRYWTLGGSLRNVPIGGSTRKRPRLAHHQHQQHARRAPAAAAAAHVFGLGLGLGGAAPPPMMMMPPLPCSSSSSSLQGQGQGQGQGGGGLLGSLFALGAAGAPPLLEGRGAGSSSSFDFDLGLGLPTAGPLHLGEAAAAVQMQGLGLRGGGGNGNAAGSSSSFLWPAGLLADNDDSVDTWTKMPPGAGAGSMWPDFFSSPPAAAAPQTGGMMLHGGAHLM

>SbDof10

MDQLKSVNDVNAASLPLLLHPVISNPSPTSSSSTSSRSSAQQQQRSTSATSSPQGQGPQQQGQGQGAEQTPLRCPRCNSSNTKFCYYNNYNLTQPRHFCKTCRRYWTKGGALRNVPIGGGCRKPRPMPTPVAKPVISSCKAVGGGVPSLGLGVGLGMGMGAGPGPWASSQQAAAAQLMALLNSARGVHQGGGNMHRLLGLDAMAHLPLHVLPGAGNNNAGGTAPSLWPQSAPRAIPTPPHMDSQLGMGPLGQHDVLSSLGLKLPPPSSSPAAASYYSDQLHAVVSSAAGRGGHEYEAAASGAMSLPCTTALTSLPPAASSVSAALTSCATVGLDLPPVSVPASEMQYWAAGPAAMSVAWPDLPTPNGAFP

>SbDof11

MLSSHHEAMLPYAPGPPPSLLVDRRYKQGGEAAPNCPRCDSPNTKFCYYNNYSLSQPRYFCKGCRRYWTKGGSLRNVPVGGGCRKNRRGKSSLRSAADAAIASGGGRDAAFGHHRFPGPVRPDLVLEGMVGNPSNPAGQAMPGGVPAATDGSTIDLAMLYAKFLNHPPAEEGVNAVTPESAGQVVDEAFDTFSASSDLSPGVLAPLQFDPCHDGFGEWSAGGPVSSTGPSSTASTTAATTMLCADVSVQAAFGELNFAMDQSCFDSLGLPTDDVVGNLSSSWCSIVPGLSTLEDTKYDSLDSFPDDALSLHEDMISGTDHDWSVDCQGLEALYMP

>SbDof12

MAPAAGGFKLFGKVITRTQCAAETAPPAVPTQEQEPAAAASRSTAAFARERDDPDERDQPMVKREAAAAAASDHDFVVVADKQQQQHSAAAGGPAPASESDDSKGQQQHPRPRQHHQQQHQDTVEARAAAAASSAPPLPCPRCRSRNTKFCYFNNYNVNQPRHFCKDCHRYWTAGGALRNVPVGAGRRKNRPLGPVGAVPVPVPVPVPVPAHLHPAAAAGFVLGFPGQHPSSPTTSPSTAVYAERWPVCPDRRF

>SbDof13

MASHLPDADAAGFKLFGKVIQPPDAHHRAADEGGAPPQLPPPTTALPPPPPPPPSPPLPPQPPLPLQQQATGATGGTSGGGGEPLPCPRCGSRETKFCYFNNYNVRQPRHLCRACRRYWTAGGALRRVASASPGRRSPRPTSARSVAAAAAAAAAASSAAAAAAEEVGGER

>SbDof14

MAAPGGGLDQDRGRGRGQDAAIKLFGRSIPVLHSSVVAAAASEVSTKLANDVRSNDGMSCLPNMPLIVKASPFPSKNNMKNGLQAISSQHGKMEADSKSEEVKNSLQAIISQPGKTDDSKSEETKTESGGSGQEKVLKKPDKILPCPRCHSMETKFCYFNNYNVNQPRHFCRNCKRYWTAGGTMRNVPIGSGRRRNKDPSHHHHVTKPCDHIVTANGDVSDATQRQSLAVKPSVLQGSGKQNETACKSVSPVLNIKEQNNADLISLVSGDNKEEKSCASSVVSGSSENWMPENTVKKEEDSTSAYGNGVKEPDPNTQSHHAGPISVFSGNPAAVMVTNQSSADGIHGPGNGTVSPLSLPPPPMVPTPGICAPAVPFPLVPAFVSCIPGWPSAVWGAAWPGSSGPTLLSLPPNSLAFSGSNSRVLGKHTRVANLQEEQKAEKKFWVPKALRIDNPEEAAKSSIWASLGIKPDERIIFKSFQSKDLKNSETKTPESLQANPAAFSRSQTFQERT

>SbDof15

MSDQKDPGIKLFGRVIPLEPEPAPGTTEAEDPPPSHDQPPDELQPRAPELAAAADEDQHNEKEEKPASEMVNMPQEKDKEIKVDTPQVEKDNEMKVDAPQKEHDDEMKIDAQQEKKDEQMEVNGSPMHENIEPANLPPSEHKKEDEDLMNSTEDKAASDPKGENEKTSNEESGQDKALKKPDKILPCPRCNSMDTKFCYYNNYNVNQPRHFCKNCQRYWTAGGTMRNVPVGAGRRKSKNSSLHYRQLLMAPDCMLGSRVDISKSVLPEALVSPPAPIQPTSRNETVLKFGPEVPLCESMVSALNIDEQNVNNSGSAPRGENREDNPGPGTSYNGVPENMVHVDKNGAPVHCNGVAPVPQYYLGTPFMYPWNVGWNNVPVMAPASRFPSPAFPYPLVPPALWGCLSGWPVTTWNIPWIRANGCVSPSSSSNSSCSGNGSPTLGKHSRDSNPMKEEKREKSLWVPKTLRIDDPDEAAKSSIWATLGIKPGDPGTFKPFQSKVESKGQRSDAAQVLQANPAALSRSQTFQESS

>SbDof16

MEEMLMAGNANPNQNPNPPPPAPSAPGAQRAGAPAAGAAAAPSAGATGGPAGAGTERRARPQKEKALNCPRCNSTNTKFCYYNNYSLQQPRYFCKTCRRYWTEGGSLRNVPVGGGSRKNKRSSSAVSSAAAASTSAAMSGTVSVGLPAKNPKLMHEGAHDLNLAFPHHNGRALQPPEFPAFPSLESSSVCNPGAAGMVGNGAAGRGMGALSAMELLRSTGCYVPLQHVQLGMPAEYAAAGFALGEFRMPPPPQSHSVLGFSLDTHGTGGVGGAGGYSAGLQDSAAGRLLFPFEDLKPAVSAAAGGGGASNGADHHQYEHSKDQAAGDGGSGPSGVTGGHETPGFWSNSLIGNGSSNGGGGPW

>SbDof17

DPIKSTREPLYTYPNREKLNETLGVLIKDEAKHKGIVPEEPGMGSSSPSAELIACPRPMHAVAAAAAADRRLRPQHDQPLKCPRCESTHTKFCYYNNYSLSQPRYFCKTCRRYWTKGGSLRNVPVGGGCRKNKRASAKKPPAPPMQPRHMAETGLHLSFSGMPQLPPPSADPLCSLGLLDWKYDPILTGSGGGAAAGSLDGASSEAHFAGAGMLGIPGGGGSGGECHALSALRYAAGLGEHLQLQGLPFGGRAEHDGMEVKPPATERLLSLDWYSETSRAPESAISSLGALGLWSGMLGGAHQHHGSSAAI

>SbDof18

MQEFQSIPGLAGRLFGGAGAGDLRRAQAHAQQGPGARCGGVSPAAPPEVVKCPRCESTNTKFCYYNNYNLSQPRHFCKSCRRYWTKGGVLRNVPVGGGCRKTKRASSSSSATSSVPSTPTSGSGDAAANKNPRRASASSPRSTNSGSASPTAAAATTPTPAPTTPATPSSNSSVAVFTTSSHHHSSPFSTIDVVAPPAPIFADQAAARASLFAPPPPPPLPVFTFAAQPKQEEAPTTTSELQLVAGLSAAAPSSSSVVSEDMAPFASLDAAGIFELGDAASAAAYWNAGSCWTDVVQDPSMYLP

>SbDof19

EAIVSSPIIKEEARSPKQAQVTQQASGSGERKPRPQLAEALRCPRCNSNNTKFCYYNNYSTSQPRYFCKGCRRYWTHGGALRNVPVGGGCRKNKRTSGSISASGTSSSSSAAYAPLSPSTNTSSSKMSINTQLMMVPNMMMSTSSMTGLFPNVLPTLMSATEGGEFNFTMDNQHASLPFTPMSLSNQASVPVLAAGESGTMPSFLEMLRKGLLHGSSSYDTGLAMSDGNNGMDMSFPLPAYGAMHGHGLSGSTTNDARQLVGTQQGVNTGGGFVGSTGVQEEEEEGDNKAMVKSNKNNNGGSLLDRYWIKPNNNNNKRQQG

>SbDof20

MEEMLMAANAGAANPSQGSNNPNPPAPAPGGALRGGGAPAAPLAGAGSTERRARPQKEKALNCPRCNSTNTKFCYYNNYSLQQPRYFCKTCRRYWTEGGSLRNVPVGGGSRKNKRSSSSASASASTSASVTSSSMASAEGAAASKNPKLAHEGAHDLNLAFPHHGGLHAPEFAAFPSLESSNVCNPGGGMTSNGRGGGAGPAVGALSAMELLRSSGCYMPLQMPMQMQGDYTAAEFALGDFRTPPPPPSQSVLGFSLDAHGPGSGAAAAGYGSSAGLQGVTENAGRLLFPFEDLKPPVSSGGGGVAGGATGGAGDGNSNHNQFDHNKEQDGGGGPGAGHDTPGFWSGMIGGSGASW

>SbDof21

MAPAASILSATAAAAGASKRPADSDAELSLDSSALQQQGDEAVRKGRQTRQQQQLECPRCRSTNTKFCYYNNYSTAQPRHFCRACRRYWTHGGTLRDVPVGGASRRSSGGGGKRRRVSAEPSSAASSSPPPLPAASLADACLPDLTSAFPFLSDGSFFPQFDVGSGVALAPAAFSSSWQSVVPDFYDGLAPWDDGAAAGTAAGGFVGAWGDIAGLDLSWTPPGN

>SbDof22

MDGVSTSPSSVTATATIREKPKPAPAATAPASLYLAVLRPSGSPSPSLSLYATGPRASQPEPGTTAPSRMQEVSVEPGRRPAQQPHHQFAGVDLRRPKGYATAPVQEATPAVKVAEGGDPCPRCASRDTKFCYYNNYNTSQPRHFCKGCRRYWTKGGTLRNVPVGGGTRKKPSSSSYAAAAAAAADADNKPPKKKPASKKRRVEAPVPEPAAAADASGVTDTVAAADSAKTTTTTTTTTTDGASEITTETEAAVAVAVPVAEEDSESLAHLLLQPGAEEAVSLGLGLSDFPSAAGKAVLDDDSFVWPAAFDLGTCWASAGFADTDPASLFLNLP

>SbDof23

MGEGRAGDGLIKLFGKTIPVPETPAAGDAAKDIQQSGSSGTTDLKGQENTLQDSTGSPPQQEVADTEDSSAAKNSSADKQQGEAANQKEKLKKPDKILPCPRCNSMDTKFCYYNNYNINQPRHFCKNCQRYWTAGGAMRNVPVGAGRRKSKSASAASHFLQRVRAALPMDPLCTAAKTNGTVLSFGSDMSSLDLTEQMKHLKEKLIPIAGIKNGDERSVGSCTEGPAKAEDSNQKENVTAEKSAKLVQHPCMNGVAMWPFSCAPPPACYTPGSIAIPFYPAPAAYWGCMVPGAWNAPWPPQSPSETGSTLSTASPASTKSNCFTPGKRPRDCNEEGDTKGNGKVWVPKTIRIDDVDEVARSSILSLIGINGDKAGKDGKGCKLARVFEQKEEARTATHSVINGLPFLQGNPAALSRSLTFQEGS

>SbDof24

MVSSPNIKEEARSPKQAEATQQPSGSGERKPRPQLAEALRCPRCNSNNTKFCYYNNYNTMQPRYFCKGCRRYWTHGGTLRNVPVGGGCRKNKRASRSVSGSGSSSSSAAYAPLSPDTNTSSSKMSINTQPMMVPNMMMPTPTMTGLFPNVLPTLMSTGGGSHFNFTMDNQHASMPFTPMPLSNQASVPMLAAGGSGTMPSFLEMLRRGLLHGSSSYDAGLVMSGGNNEMDMSFPLPAYGAMHGHGLSGSTTDDARQLVGTQQGMNTDSGFAGSTRVQEEEEEKGDNKAMVKSSNNNNAQQQQQQQQQQQQQQQQQQQQGAAGVVCQAVEVLLLQTI

>SbDof25

MKLSSSSHHLLLESSNLTLSPHHRNTAVVTRMLSSHCENMLPYAPGRRAAVLLDHRRYRPNVVEVAPSCPRCDSPNTKFCYYNNYSLSQPRYFCKGCRRYWTKGGSLRNVPVGGGCRKNRRGKPVVRAVAVDAAVAAASGGASALANRSSSSSPATLRPDLLLEGMIGSPVGLCQPTDDDAAEIPAVVAPEGSTIDLALLYAKFLNHQPAAAAEPCAAAVVPESLDTLSGSSTSGDVSPVVVPPRDQHQPFTTQDHGGFGELSATTASAEPSAAPPQRPADDACACAAEALIGALSVDPRCYDSLGLPPDGGDLVLPSTWHLGTKYEPFDPLPEDAMSLQDGFAGDEDVWSSALACQGLEAALCRP

>SbDof26

MGGPLPDGGGGGGGGAGGQVVGGPAKPMSMAERARLARIPLPEPGLKCPRCDSTNTKFCYFNNYSLSQPRHFCRACRRYWTRGGALRNVPVGGGYRRHAKRAKPKQQQAAGAGAGGGSAGTGNTATANAALQHAPAGSTASSAAACTATTTNALPGGMLGGGGLSMLPPLLRLADFDAMSLGSTFSGISSMGKPGSIDAYSHSVGGGGAPAGLEQWRVQQMQSFPFLHAMDQGPLGPPLAMAMAAPGMFQLGLDTTSSDNGHGRGGGGGEDGSTGGELHVMHQQAATKRESYPAPRGMYGDHHHHLAAAGGYTSAYSTNAAT

>SbDof27

MEAGQVPDGRALMAAVTTTGGGGREPEGLPCPRCESVNTKFCYYNNYNLSQPRYFCKTCRRYWTRGGALRNVPVGGNTRKATPATGRRKRSTPAPVNVTVPAPATASPPPPPALHGGSLLRPYGGGGGSGLLSFAAPALASPLAAADPDRRLLDFGGSFTSLIAPGVADVGVHFSAGFLMGGLAPAALPRAPGSVAALPPPPPQQQPTVSQALPEGMVWSMGWPDLSI

>SbDof28

MQMQQQPPLQCLLGGGGGGSDHHHLMPPPSGLAPLPGGPADTAASAPAGGGSSTSMQAAAGAGTAAAQPRPVVSMAERARLARVPLPEPGTLRCPRCDSTNTKFCYFNNYSLSQPRHFCKACRRYWTRGGALRNVPVGGGCRRNTKRSSKKSSRGGGGAGATAATSSSSTTSTSTTATTTTATTTSAAMAAAEAIASMQAQLPHLGLPPAAAAAALEASLEGYHHYLPLQMQPQFLQQAGLHGYHFADDGTGVLADGFPRGVVASGLLAQLAAVKMEEHSSNGGGAVAAHHEQSYWPGSTGGGSGWPAEFLSGFSSSSSGNVL

>SvDof1

MEKVAIVKQLGNVDEDRSFGVVCWCRVPEAAMVASPPREEAVAARNVKAKQARQQQVVASGSGERKPRPQQDQALNCPRCNSTNTKFCYYNNYSMTQPRYFCKACRRNWTLGGTLRNVPVGGGSRKKKQNPAGGSSASSAPPASSSSSNDSKKMNITQQLLTMPTATTPMPADFPNVLPTFMSIGGSFQLPSSDQHSLPFAPLSLSSNPGTMLSFMERGGFLDGSSSNGMASLPILPVPSFGVMQHGHGMMGGSSDQQMMGPLQGVDQEVKPPMATAGGSGLQQWPSSTTQEQQVVGGDGSADNNNDNNMDGGASGSSSGVERYWQGGFN

>SvDof2

MDAAQWHQGLGLGKPMEEMLMAGNTNPNQNPNPPPAAPSAAPAAQRAPGAPAAAPPAPAAAGAGAGTERRARPQKEKALNCPRCNSTNTKFCYYNNYSLQQPRYFCKTCRRYWTEGGSLRNVPVGGGSRKNKRSSSAVSSAATAAASTSAAVSGTIPVGLAAKHPKLMHEGAHDLNLAFPHHNGRGLHPPEFSAFPSLESSSVCNPGATMAGNGAAGRGVGALSAMELLRSTGCYVPLQHVQLGMPAEYAAAGFALGDFRMPPPPHSQSVLGFSLDTHGTGGVGGAGGYSAGLGVQESAAGRLLFPFEDLKPAVSAAGGANNNGADQYEHSKDQAGDGSGASGVTTGGHETPGFWSNSIIGNGSSNGGGGPW

>SvDof3

MMAGVPPMHICMDSDWLKGIVPEEPGMGSSSPSAELIACPRPVHAAAADRRLRPQHDQPLKCPRCESTHTKFCYYNNYSLSQPRYFCKTCRRYWTKGGSLRNVPVGGGCRKNKRASAKKPAAPPMLQAAHARQVAETGLHLSFSGMLQLPPPTAAADPLCSLGLLDWKYDPILAGPGAAAGALDGAGSEAQFSGAGMMGIPGGGECHALSALRYAAGLGEHLQLQGLPFGARAEHDAVEVKPAAAAERLLSLDWYGEAASRAPESAISSLGALGLWSGMLGGAHQHHGSSAAI

>SvDof4

MQEFQSIPGLAGRLFGGAAAADLRRAQAQQGPGARCGGVSPAAAAPEAVKCPRCESTNTKFCYYNNYNLSQPRHFCKSCRRYWTKGGVLRNVPVGGGCRKAKRSSSSSSPSSAPSTPTSATDAKNPRRASASSPRSNSGSGSGSASPTAAVATAPTTPATPSSNSIGLTSHHTNPFSTVDVAPPAPIFADHAAALASLFAPPPPPPLPAFTFTAQPKEESAAAASAQHLAGQAVPSEAPSSASTDMTPFASLDTSGMFDLGDASAAAYWNAGSCWTDVHDPSMYLP

>SvDof5

MQEPGRRPAPPFAGVDLRRPKGYPAPAARAAEEPASASSASAQAQPPAGDPCPRCESRDTKFCYYNNYNTSQPRHFCKSCRRYWTKGGSLRNVPVGGGTRKSSSSSSASSSSLAAAPKSTKRSKNSKRRRVAPAPDPVPATEASATTTRVANNAPVPAPSTVAAAMVVATGKPTASKPAPPVAAAEKTTAPEPAATVAAMDEKLTAPPAAVGCFTSEPSTAPGLGLADVGCGGGKELPPDPNHFEWPSGCDLGSYWSTGVFADTDPALFLNLP

>SvDof6

MAGAGGATAVQQPAAGARSGAGAAGAPVADPRAEALRCPRCDSANTKFCYYNNYSLSQPRHFCKACKRYWTRGGTLRNVPVGGGCRKNKRSRSSSGAAGAAGRSGSSAAAAAAAAATSSSAASALSLPPPAGSLPSLTSALGLSGGTSLASLLLGTGAGGDHLGLFHAAMQSVVSSEATAYEMQQQQQTQVDHLLGLGYGGAAAQIQLKPWMQDAGAGGAGGIMDSFYAPLLSSSLVPGLEELHVKAEAAGAGDHQQKAAPGDQQSGSWELPTPSSSNVDASIIASDALMAAAASMNPAVSSTSTAATTAPSSFMYWGNGGIGGAAAAWPDLANCGSSIATLF

>SvDof7

MELAGAASPRSPESHVAPPRPPPQPPEKDACEDTGDMSMAVEKPCTRQEVDLGQTNRSSLNSSSECENQAASNDEMTGSESNLETAKTEGDVSNGEKVLKKPDKILPCPRCNSMDTKFCYYNNYNIKQPRHFCKSCQRYWTAGGSMRNIPVGAGRRKSKSSSANCRSILIPGSSVATPAGEAALFPLSINGNQAAVNFGPDSPLCNSMASVLKIGGEQSKNANPASTAQPRNGETQTCPASTTASDGPRSESHKGAVSAHQNGVVGHGNGVTSIHPIPFFPGPPFVYPWSPAWNGIPALAAPVCPAPAEAANSSENGNSSCNAQWNVPPMVPVLPPGFCGPPFPVPVMPSSVWPFITPWANGAWSTPWLGPSSSVPASSPTSSSTCSDSSSPVLGKHSRDSRPQGDEKAERCLWIPKTLRIDDPDEAAKSSIWTTLGIEPGERGMFRPFQSKPEGREQISSSAKVLQANPAALSRSQSFQEKT

>SvDof8

MAPAASILSATSAAAASKRPPASDAEPLLQGDGEAARKGQQPSRQQQLECPRCQSTNTKFCYYNNYSTAQPRHFCRACRRYWTHGGTLRDVPVGGASRRTGGSKRRRVSAEPSPSASASSPPQTTAGADAFLLAPDLSAFPFLSDGSFLMPPQLDLGVAPAAFSSWQSVVPDFYDGLAPWDDGATGMTGPWGDIAGGLEP

SWPPPGN

>SvDof9

MIFPPAFLDSSSWNDNQQQQQQAHHQVAGGGGGGGDGNHELLQPSIMGGALPEGAGGGGGAGQVGPAKPMSMAERARLARIPLPEPGLKCPRCESTNTKFCYFNNYSLSQPRHFCRACRRYWTRGGALRNVPVGGGYRRHAKRAKPKAAAAASAATSTGTASATAAAAAGLAPAGSTSSACATTNVPALQGPAMLGGNLSMLPPLLRLADFDAMSLGSSFSGMAGKPSLDAAGAYSVGGGSGLEHQWRVQQMQSFPFLHAMDQGPLGPPLAMTMAPGMFQLGLDSGDGRGGGGGSGEDGSGELHVMQAKREGGYQARGMYGDHHFAAAGYASYSNNAAAGNHLL

>SvDof10

MADTSLLCFGENHRKPAIHTYASRDMLPHVELPVRAAAACTGAAGIKLFGKVITTQQQPLPAPPMHAGGNAPPRLQNQAPPAPGRGSADLLEEAARARAAAAEARLPCPRCRSRDTKFCYFNNYNVNQPRHFCRACHRYWTAGGAIRNVPVGSGRRKNRPVLPPMPPPHAATNTGADHASSGSGSPPVFAASGLAVPYRGSPFHLAPPSPACTAASGLPEATTGQYWWLVAGGAVASSDRAF

>SvDof11

MHHRDIVVVTRMLSPHYDNVLPPYAAGRRAAALLDHRRYRPNVEVAPNCPRCDSPNTKFCYYNNYSLSQPRYFCKGCRRYWTKGGSLRNVPVGGGCRKNRRGKPVRAMPVDAAATGTAGGATAPYYQRSSSFPGTLRPDLLLEGMVGSPAGLCQPMEAAADKPAVVEGSTIDLALLYAKFLNHQPPPAVEPCAILPESLDALRGSSSDMSPDVPPPPDHHPFTRQDAFGELSTPASADPGAAAPQCPDARTEVLAELGFSVDQSCYDSLGLSTDDGDLILPSTWQPEAKYEPFDPLPEDAMSLHGGISGGDDVWSSALACQGLEAALCRP

>SvDof12

MVFSSLPIFLDPPNWGQMQMQQQQPPLQCLLGGGGGGSDHHHLMPPPSGLAPLPGGPADTAASAPAGGGSSTSMQAAAGAAAQPRPVVSMAERARLARVPLPEPGTLRCPRCDSTNTKFCYFNNYSLSQPRHFCKACRRYWTRGGALRNVPVGGGCRRNTKRSSKKSSRGGGAGATAATSSSSTTSTSTTATTTTATTSAAMAAAEAIASMQAQLPHLGLPPAAAAAALEASLEGYHHYLPLQLQPQFLQQAGLHGYHFADDGSGVLADGFPRGVVASGLLAQLAAVKMEEHGGGGGGGGAVAAHEQPYWAGSNGGGSGWPAEFLSGFSSSSSGNVL

>SvDof13

MEAPLHQSPVPLPLPPPQEEAVIGAALLRQAEAARAMMAAAAAAQHQHQQPPPAAAMGREQCPRCASRDTKFCYYNNYNTAQPRHFCRACRRYWTLGGSIRNVPVGGSTRKRPRPARPTRALAAATAAAATTPASSAPFDTSSSSSPVAPAAALQGGLLGSLLLGSASASTLLALGAAPLLEGRLGFGPGLGQPALLAGANAAAGDLSRLDFGGAGPLLWPAAATVLEGDRAFPLPPPAAALWQKELAAAAPPPVEAGGLHHGGSPHLLL

>SvDof14

MQELQPIPGLAGRLFGSGGAAAAAIGLLRRHGGSSAAEVRCPRCDSPDTKFCYYNNYNLAQPRHFCRACRRYWTKGGHLRNVPVGGGCRKPRPRRPAAAAADGRGKDGVHRDGKAPRSGFAGAASSSSPTAAGDADAPVSGAFSVVTEPSVPRSGGVAEASAETGSFAAGDTRALLVPPPAPMFADQASVFASLFAPPRPLSAFGSSAQPQPEQAEERVAASLLAAEQPPPSCTAAFTDTAPFAAGSDGALSAGPSDWPTAGIFELAGGNAGDASLPEHWNHGSWTDPDPAVYLP

>SvDof15

MDASSGLDCDQYEDPEWHAAIQLVGLCVPVAGAPNSDAAAAAPEVSIRSLISLSASRNHSFVVAGCSWGERRARKLNRARLFLLSIALSNAVVWAMVRADRGFRSPMLPSFSYGNSSAAMCVFLARDHLVGRRRGGEGVGERRLTVVRWTAAACARTSGANKAPAPMSWAQSGSQPIWTSEVPEASERAAEQAPVRVSTKNDADGSGKEKEFKKPDTVLPCARCNSMRTKFCYFNNYNANQPRHYYCKDCKRYWTDGGTLRDVPVGSARRKNRSNANGDVAGTSRRRSRVFKPLAIPGPAAEESGAATTSGSEMVLGKPLCPAHNTEEQKNSTDLVGPGDNKEEKSCLSSAVAPSGSSDNSGSNSAAVTASTQSSTDGTQELVNSGVSRLLLMPTTMPGPGIRAPAVTFPQVPFYWSCIPGWPNGASSVPWPGSSGTTLPPVPACSGSLALGKHPREEEEAEKTFWVPKALRVTDPEEAAKSTIWASLGIKPDERILQSKDENGKTPESPAACSISCSLTFQNRT

>SvDof16

MSDQKDPGIKLFGRVIPLAPEPAPEATETEDPLGHDQPPEELQPRAPEEEAAAVADEGQHNEKEDTEDSEMEVDTPQEKGSEMKVDTPLEKGNEMKIDTPQEEKGNEMKFDASKKEQDGEMKVDAQQEKKDEQMKVDAPPRAENIQPGTSPRSDHKKEDQGQMNSTEDKAASDPKGESEKANDESGQDKTLKKPDKILPCPRCNSMDTKFCYYNNYNVNQPRHFCKNCQRYWTAGGTMRNVPVGAGRRKSKNAPLHYRQLLMAPDCMLGSRVDISKSVLPEALVSPPSAPVQPTSRNETVLKFGPEVPLCESLVSALNIDEQNAKNPGSAPRGENREDNSCASAVTSYNGLPENTVHVDKNGAPVYCNGVAPVPQYYLGTPFMYPWSVGWNNLPVMVPGKSMPEAASASESCSTSSAPWMTSPMMPASTSPAFPYPLVPPALWGCLSGWPATTWNIPWVRTNGCVSPSSSSNSSCSGNGSPTLGKHSRDSNPLKDEKQEKSLWVPKTLRIDDPDEAAKSSIWATLGIKPGDPGTFKPFQSKVESKGQKADAAQVLQANPAALSRSQSFQESS

>SvDof17

MVSCAASSPSSAADMEAGQAADGGALAVVASVAAATGGRPGVAGGGREPEGLPCPRCESVNTKFCYYNNYNLSQPRHFCRACRRYWTRGGALRNVPVGGGTRKATPATRRKRTAGGTPPAPAPAPLVAALPPLTMSGPHGALLRQYGGLPFPAPALASPLAAVDPDRRLLDLGGSFSSLIAAAPPLDVGAHFSAGFLVGGLAPALAHSPAAAAALPPPPPPPQQVPQALPEGLIWSMGWPDLSI

>SvDof18

MANLPDADAAGFKLFGKVIQPPDAHRAAEEGAGAPPPPPPPPQPPTPTPTMAVLPPAPPQSPPPPPPPLQLQAAGAGAAAGGTGDPLPCPRCGSRETKFCYFNNYNVRQPRHLCRSCRRYWTAGGALRRVASASPGRRRPRPTARSAAAIATASSASAAAAEEGGGER

>SvDof19

MLSHVEMAPAAGGFKLFGKVITQCAESAPPVAAVPPQEAAASRSTAFARERDDPDERDQPMVKREAAADHDELVEKQQQDSGGGGAVESGESKGQQHPRPRQQQHQDTAEARAAAAASSAPPLPCPRCRSRNTKFCYFNNYNVNQPRHFCKDCHRYWTAGGALRNVPVGAGRRKNRPLGPVVAAGAVPTHHLHHQHRAAAVAAAAAAAGAGFVLGFPGQHPSSPTSPSPIYAERWPVCPDRRF

>SvDof20

MLSSHHEAMLPYAPRPPSLLVDRRYKQGGETAPNCPRCDSPNTKFCYYNNYSLSQPRYFCKGCRRYWTKGGSLRNVPVGGGCRKNRRGKSSARSAAESMASGRDAAAFGHRFPGPVRPDLVLEGMVGNPANPGQAIPDMPAAADGSTIDLAMLYAKFLNHPAAEAGAGAVTPESAGQVDEAFDTFSASSDLSPGVLAPAQFDPCHDGFGEWLGPVSSTDPTSTAASTSSATTMLCSDVSVQAALGELNFAMDQSCFDSLGLPTDVGNLSSWCSIVPSLSTLEDTKYDSLDSFPDDALSLHEGMISGPDNHDWSVDCQGLEALYMP

>SvDof21

MQGAAAEPGRRLAQQQFAGVDLRRPKGYAVPAPAEGDPCPRCESRDTKFCYYNNYNTSQPRHFCKGCRRYWTKGGTLRNVPVGGGTRKKPSSSPPSSYAAAANANKPKKPSKKKRRVVVVAPQDPVAAPVAAPTLVAAPAADSAKTTTNETTTTTTNETTATTATPTTTDAASEITTELVVPAAEEEDSLAHLLQPDVALGLGTSDFPAAGKALDLEPDSFEWPAAFDLGGACWGSAGFADPDPAGLFLNLP

>SvDof22

MCSRSSSGSTTATHNPTRAQTKSLGLVKPMEEMLMAANAGAAAANTGQGSNPNPPAPAPAPSSVPGGGALRAGAPPAVAGAGSTERRARPQKEKALNCPRCNSTNTKFCYYNNYSLQQPRYFCKTCRRYWTEGGSLRNVPVGGGSRKNKRSSSSSASASTSASVTSSSMASTAGAASKNPKLAHEGAQDLNLAFPHHSGLHAPEFGAFPSLESSSVCNSGGAMASNGRGGGGGGAGPAVGALSAMELLRSSGCYMPLQMPMHMPGDYAAGGFALGEFRAPPPSQSVLGFSLDAHGPGPGAAAAGYGSSAGLQGVPENAGRLLFPFEDLKPPVSSGGGGVATGASGGDGDGNSGHPQFDHNKDQGGGAPSAGHDPPGFWNGMIGGSGASW

>SvDof23

MGRKEEAAIPPPAAARNVKKAKQQQAGGERMPRPQQEQALNCPRCHSTNTKFCYYNNYSVTQPRYLCKACRRYWTKGGTLRNVPVGGRCRKNKQQNPSAPSALASSSDSKKIMNSSTQQLLMMMPPPPPTAANLSNVLPTTFMSATGVGGGFELPSSDHHPLPFAPLSLPSNPGTTPPASSFLDLLPFLPTPSSFGAMMLQHGPGMIAGAGGGLQQQWLPSSQHGNDDGGLFAAGGSPAAAAVQEPQQQQEEEVGGGDGGTAADAAGNDDMGGGGASADIINIYWSSSRI

>SvDof24

MDMTSSTTSNTTAQSVVQNKQEAAMVASPPREEAAAARNIKANQAWQQQVAATGGGERKPRPQQEQGLNCPRCNSTNTKFCYYNNNSMTQPRYFCKACRRNWTQGGTLRNVPVGGRSRKNKLNRAGGSSSSSTPPAPSSSSNDSKKMNLTQQLSMMPTATAPMPADFPNVLPTFMLTGGGFELPSSDHHSLPFPPLSPLFNPGTTPSLLDILTGGFLDSGNNRMAASPLLGTMQHGHGMMGGSHDPQLVGLLQGVDQALELPMPAAGGGFQQWPSLAAQEQQVVGGDGSADMDDDNNNNGGGASGGSSGVENYWQGSI

>SvDof25

MELISSTTNNTTAPSAPQNKQEGAMVASPPREEADPARNVKAKQAQQQQVAASGASERKPRPQQEQGLNCPRCNSTNTKFCYYNNNSMTQPRYFCKACRRNWTQGGTLRNIPVGGGSRKNKQNRAGSSSSSSPAPPAPSSSSTNSKKMNQLTQQLLMMPTAMAPMPADFPIVLPTFMSTGGGGFELPSSDHHSLPFPPLSLPSNPGTTPSLLDMLTGGFLDGGIGALPFLPTPPSFGAMQHGHGIMVGSHDQQLVDPLKGVDQALKPPMAATGGSGLQQ

>SvDof26

EAAMVASPSREEAAAARNIKAKQERQQQVVASGADERKPRPQQEQGLNCPRCNSTNTKFCYYNNNSMTQPRYFCKACRRNWTQGGTLRNVPVGGGSRKNKKNRAGGSSSSSSAPPAPSSSCTNSKKMNLTQQLLMMPTAMAPMPADFPNVLPTFMSTGGEGFELPGSDHHSVPFPPLSLPSNPGTMPSLLNMLTGGFLDGGMGAQPFLPAPPSFGAMQHEHAIICGSSDQQVVGPLQGVDKALKPPMAAAGGSGLQQWPSSAAQELQVVGGDGSAFNNNNGNNNGGGASGGISGVEHYWH

GST

>SvDof27

MVASPPREEAAAARNVKAKQAWQQQMAASGGGERKPRPQQEQGLNCPRCDSTNTKFCYYNNNSMTQPRYFCKACRRNWTQGGTLRKVPIGGSSRKNKQSRAGGSSSSSSSAPPAPSSTSNDSNKMNLTQQLMMMPTTTTPMPANFPNVLPTFMSAGSGSFGELPGSDHHSLPFPPLSLPSNPTGTMPSLVGILRGGFPDGGMAALPFLPVPPSFGAMHQHGHGMMGGSSDQQLVGPLQGMDQALKLPLAAACGSGPQQWPSSAAPEQQVVGGDGRADKNNNNNGGGASGSSSGVEYYWHG

SI

>SvDof28

MVFSSVPVYLDPPNWNQHQHQQQQQQAHHGQLPSGGGGGGGGGGGVETHAHHHQQHQHQHQHQHQHQHQHHQLPPMPPPGALMAPRPDMETIAVAASGGGGGGGGPTGGSAVRPGSMTERARLAKIPQPEPGLKCPRCESTNTKFCYFNNYSLSQPRHFCKTCRRYWTRGGALRNVPVGGGCRRNKRTKSSKSSSSSAAASASATGGGTSSSTSSTATGGSSAAAAVMPPQGQLPFLASLHHPLGGDHYSTGASRLGFPGLSSLDPVDYQLGGAGGGGGTAIGFEQWRLPQIQQFPFLSRPDAVPPPMSGIYPFDVEGHGDPAGFAGQMLGGSKVPGSAGLITQLASVKMEDNPPSTAMTSLPREFLGLPGNLQFWGGGGNGGASGNNGGTANTGGGGGGSGASAPGSSWVDLSGFNSSSSGNIL

>SvDof29

MACPFSSPSSNFFISHTPVPLPHPTTHPLIHTTKQEPESNHTTPPLPSPRKNPSPSASRSLFELEFSPSITMIQELLGGAAMDQLKSVNDGNPASLPMLLHPTVSNPSPTSSSSTSSRSSAQQPHQQQRSTSATSSPQGQQQVQGAEQAPLRCPRCNSSNTKFCYYNNYNLTQPRHFCKTCRRYWTKGGALRNVPIGGGCRKPRPMPTPVAKPALSCKAVGGAPSLGLGVGLGMGAGPVPWASSQQAAAAQLMALLNSARGVQGGGGHGGSNMHRLLGLDSMAHLPIHVLPGAGNASGAPASLWPPAASRPIPTPPPHMDSQLGMGPLGQHDVLSTLGLKLPSPSPSLAASYYSDQLHAVVSSAAGRPHEYDAPGTTSLPCTTAAASLPPPASSVSAALSSATVGLDLPPISLPAPEMQYWAGPAAMSMAWPDLPTPNGAFP

>SvDof30

MAGQVMEAHAHHQARLQPPAVPPFAPLPPSCKNDVHHFPAAMATPGSNAAAAADMAAYLQQLQDTAAAAAEANKSSSGGNGGGAARGEQCPRCASHDTKFCYYNNYNTSQPRHFCRACRRYWTLGGSLRNVPIGGSTRKRPRLAHHHHHHQQQPAAARRAPVFGLGLGGGGGAHPPMPSSSSSQAGGLLGSLFALGAAAPLLDGPAAPMLEGRGAGLFDLGLGLPVPGGAVGGAGDAGVQMQGLGLIRGGGGGHAGGASSGLFWPAGLLDSDSVDTWKMLPGVGAGAMWPEFSAAAAPAPQAGGLLHGGAQLM

>SvDof31

MASSTPVAAGDDAVGTRKGGTGGGGGAAPPPPATQQQQPPPPPEQGLRCPRCDSPNTKFCYYNNYSLSQPRHFCKTCRRYWTKGGALRNVPVGGGCRKNKRSRSAAAAAAASRLSLNLPVEGVGGDQQAARLGFLGATGSAPVASSPIGGGGPAADYQQAAGGAVGMMALPRLHALGVGQYVPFGEWPSGAGGDISSGGGGGHAMSGGGAHGGAVSSNIASSIESLSFINQDLHWKLQQQRLATMFLGPPPPPPPTSSASHIDGAPAAAPAHIGGAFLQMAGPPGMESTMPAATSWFMDSSYAVLPSPHAHANNTAAAAITAAATTTNCNVGRSSGGDDDDAANCGSAIPSWGDMSTFAMLP

>SvDof32

MPSSFLASASSSASSPLSYLIPAASRPPPPPLVMGQGYSVDGLAGVPTGGVVVDATSAAAVAPPRPGARNPGHPPLPRPPPRQCPRCQSANTKFCYYNNYSRTQPRYLCKACRRHWTEGGTLRDVPVGGGRKNRRGGSKAGAAAAKASASTSAAAAAATQGGSVAVGADTFPDLLRQLMQFQPTALAGGGYAIDLSAWQQMAAATAPPQGAGDVSALGGAAAAAAEANCGALQYWAGWQPDDMPGLDGPC

>SvDof33

MMAECRGGDCLIKLFGKTIPVPEAADAAKESGSSSSSTESDAPENAHQEASDPSPQPEVVDAEDPKSSPETTQQPDAAGDVASQREKLKKPDKVLPCPRCNSMDTKFCYFNNYNVNQPRHFCKNCQRYWTAGGAMRNVPVGAGRRKNKHAVAASHFLQRVRAALPAAGDPLKTNGTVLNFGGHGPPPALHDLTEQVNHLQEKLLIPARKTSNPSPAVGPCSEGSSSTDDMTHGGGIKEKSSTVDKPANGAQYPAGMNGAAVWPYSCAPPPAYFSSGIAIPIYPAAPGYWGCMVPGAWSLPWSVQPPSQGLSSPSSAPSVSSSGPDSLTLGKHPREVDEGRSSAHGSGKVWAPKTIRIDDADEVARSSIWSLIGIKGDKKRDDADHAVGHKHGTVFEPKLEVNKAAKPGMITRSPFLHTNPVALTRSVTFQEGS

>SvDof34

MMHDMMRRKQTTKEDQTFSTMPYHGRIPTHGGGGTGSGSKEAHRYTQHQLPRLPGAPMAPHLSDTPAEASASADDGSAGGSCWVKPGCMMELARLAKIPQPASGLMCPRCRSTETKFCYYNNYSLSQPRHFCKTCRRYWTHGGALRDLPFSNSVRRRRRNKPSNNKQTSSKVACCGAFSGSTGMSPSSSPSSGATIFSGRVATAATAILKPLEQLLIGGAEHHRAGASRLWFPGHSSQDPVPLGYRHQLGNSRGAATTIRLEHQRYLPQRQPFSLLGYKNGGTAPATSAIFPFSEGAGGAEAASFAGQMHAAISRVRGSAAVTTTELASEMMAGNPTIPSTEMGTLASSPGEFLVGVQGDHDLFHFLGSGSWACGYGSTAGNNGSGGGSSCTAAPGSAWPDPSGFTSSSSGRSTIL

>SvDof35

MVFPSVPAYLDPPNWNNQQGQQPRASAGGGDAPLLPVGPAAATAAGPDNSGLPSSSSTASAAVAAQARPNSMAERARLARMPQPEQALKCPRCDSTNTKFCYYNNYSLSQPRHFCKACRRYWTRGGSLRNVPVGGGCRRNKRSSKSSGGSSSSKPYSSARQLAGPSSSTPSSTPGATGAIIPPSLGSFSHHLPFLGTMHQPGPNLGLAFSAGLPPLGMQHMDTVDQFPVASGGGATIGASLEQWRVQQQPQQQFPFLTGGGILELPPPAMYQLGLDGNNRGGSGSAAAAAFTLGQTSATTARQEGSMKVEGSKGQDMSLQRQYMAALRHGSQGVWDGIHGNAGSSGGDGGGNGGSSWPMNIPGFHSSSTGGGNGSGL

>SvDof36

MAECRVGGGGGDGLIKLFGKTIPVPEAAAAVGEADKDIQQSGSSTTELKGQENTLQDSTGSPPQQEVADTEDSSATKKSSGDQQQGETANQKEKLKKPDKILPCPRCNSMDTKFCYYNNYNINQPRHFCKNCQRYWTAGGAMRNVPVGAGRRKSKSASATSHFLQRVRAALPIDALCAAAKTNGTVLSFGSDMSSLDLTEQMKHLKEKLVPITRIKSSDDRSAGSCTEGSAKVEDSNQKEKVTADKSANVVPHPGMNGVTMWPFSCAPPPACYTSGIAIPFYPAAAAYWGCMVPGAWNTPWPPHSQSESASPLSTASPASTKSNCFTPGKRSRDCDEEGDTKGHGKVWVPKTIRIDDADEVARSSILSLIGINGDKASKDGRGCKLARVFEQQKEEAKTATHAVINSLPFLQGNPAALSRSLTFQEGS

>SiDof1

MVASPLPREEAVAARNVKAKQARQQQVVASGSGERKPRPQQDQALNCPRCNSTNTKFCYYNNYSMTQPRYFCKACRRNWTLGGTLRNVPVGGGSRKKKQNPAGGSASSAPPASSSSSNDSKKMNIVTQQLLTMPTATTPMPADFPSVLPTFMSIGGSFQLPSSDHHSLPFAPLSLSSNPGMMSSFMERGGFLDGSSSNGMASLPILPVPSFGVMQHGHGMMGGSSDQQMVGPLQGVDQEVKPPMATAGGSGLQQWPSSTTQEQQVVGGDGSADNNNHNMDGGASGSSSGVERYWQGGFN

>SiDof2

MDAAQWHQGLGLGKPMEEMLMAGNTNPNQNPNPPPAAPSAAPAAQRAPGAPAAAAPAPAAAGAGAGTERRARPQKEKALNCPRCNSTNTKFCYYNNYSLQQPRYFCKTCRRYWTEGGSLRNVPVGGGSRKNKRSSSAVSSAATAAASTSAAVSGTIPVGLAAKHPKLMHEGAHDLNLAFPHHNGRGLHPPEFSAFPSLESSSVCNPGATMAGNGAAGRGVGALSAMELLRSTGCYVPLQHVQLGMPAEYAAAGFALGDFRMPPPPHSQSVLGFSLDTHGTGGVGGAGGYSAGLGVQESAAGRLLFPFEDLKPAVSAAGGANNNGADQYEHSKDQAGDGSGASGVTTGGHETPGFWSNSIIGNGSSNGGGGPW

>SiDof3

MMAGAPPMHICMDSDWLKGIVPEEPGMGSSSPSAELIACPRPVHAAAADRRLRPQHDQPLKCPRCESTHTKFCYYNNYSLSQPRYFCKTCRRYWTKGGSLRNVPVGGGCRKNKRASAKKPAAPPMLQAAHARQVAETGLHLSFSGMLQLPPPTAAADPLCSLGLLDWKYDPILAGPGAAAGALDGAGSEAQFSGAGMMGIPGGGECHALSALRYAAGLGEHLQLQGLPFGARAEHDAVEVKPAAAAERLLSLDWYGEAASRAPESAISSLGALGLWSGMLGGAHQHHGSSAAI

>SiDof4

MQEFQSIPGLAGRLFGGAAAADLRRAQAQQGPGARCGGVSPAAAAPEAVKCPRCESTNTKFCYYNNYNLSQPRHFCKSCRRYWTKGGVLRNVPVGGGCRKAKRSSSSSSPSSAPSTPTSATDAKNPRRASASSPRSNSGSGSGSASPTAAVATAPTTPATPSSNSIGLTSHHTNPFSTVDVAPPAPIFADHAAALASLFAPPPPPPLPAFTFTAQPKEESAAAAAASAQHLAGQAVPSEAPSSVSTDMTPFASLDTSGMFDLGDASAAAYWNAGSCWTDVHDPSMYLP

>SiDof5

MQEPGRRPAPPFAGVDLRRPKGYPAPAARAAEEPASASSASAQAQPPAGDPCPRCESRDTKFCYYNNYNTSQPRHFCKSCRRYWTKGGSLRNVPVGGGTRKSSSSSSASSSSLAAAPKSTKRSKNSKRRRVAPAPDPVPATEASATTTRVANNAPVPAPSTVAAAMVVATGKPTASKPAPPVAAAEKTTAPEPAATVAAMDEKLTAPPAAVGCFTSEPSTAPGLGLADVGCGGGKELPPDPNHFEWPSGCDLGSYWSTGVFADTDPALFLNLP

>SiDof6

MAGAGGATAVQQPAAGARSGAGAAGAPVADPRAEALRCPRCDSANTKFCYYNNYSLSQPRHFCKACKRYWTRGGTLRNVPVGGGCRKNKRSRSSSGAAGAAGRSGSSAAAAAAAAATSSSAASALSLPPPAGSLPSLTSALGLSGGTSLASLLLGTGAGGDHLGLFHAAMQSVVSSEATAYEMQQQQQTQVDHLLGLGYGGAAAQIQLKPWMQDAGAGGAGGIMDSFYAPLLSSSLVPGLEELHVKAEAAGAGDHQQKAAPGDQQSGSWELPTPSSSNVDASIIASDALMAAAASMNPAVSSTSTAATTAPSSFMYWGNGGIGGAAAAWPDLANCGSSIATLF

>SiDof7

MELAGAASPRSPESHVAPPRPPPQPPEKDACEDTGDMSMAVEKPCTRQEVDLGQTNRSSLNSSSECENQAASNDEMTGSESNLETAKTEGDVSNGEKVLKKPDKILPCPRCNSMDTKFCYYNNYNIKQPRHFCKSCQRYWTAGGSMRNIPVGAGRRKSKSSSANCRSILIPGSSVATPAGEAALFPLSINGNQAAVNFGPDSPLCNSMASVLKIGGEQSKNANPASTAQPRNGETQTCPASTTASDGPRSESHKGAVSAHQNGVVGHGNGVTSIHPIPFFPGPPFVYPWSPAWNGIPALAAPVCPAPAEAANSSENGNSSCNAQWNVPPMVPVLPPGFCGPPFPVPVMPSSVWPFITPWPNGAWSTPWLGPSSSVPASSPTSSSTCSDSSSPVLGKHSRDSRPQGDEKAERCLWIPKTLRIDDPDEAAKSSIWTTLGIEPGERGMFRPFQSKPEGREQISSSAKVLQANPAALSRSQSFQEKT

>SiDof8

MAPAASILSATSAAAASKRPPASDAEPLLQGDGEAARKGQQPSRQQQLECPRCQSTNTKFCYYNNYSTAQPRHFCRACRRYWTHGGTLRDVPVGGASRRTGGSKRRRVSAEPSPSASASSPPQTTAGADAFLLAPDLSAFPFLSDGSFLMPPQLDLGVAPAAFSSWQSVVPDFYDGLAPWDDGATGMTGPWGDIAGGLEPSWPPPGN

>SiDof9

MIFPPAFLDSSSWNDNQQQQQQAHHQVAGGGGGGGGDGNHELLQPSIMGGALPEGAGGGGGAGQVGPAKPMSMAERARLARIPLPEPGLKCPRCESTNTKFCYFNNYSLSQPRHFCRACRRYWTRGGALRNVPVGGGYRRHAKRAKPKAAAAASAATSTGTASATAAAAAGLAPAGSTSSACATTNVPALQGPAMLGGNLSMLPPLLRLADFDAMSLGSSFSGMAGKPSLDAAGAYSVGGGSGLEHQWRVQQMQSFPFLHAMDQGPLGPPLAMTMAPGMFQLGLDSGDGRGGGGGSGEDGSGELHVMQAKREGGYQARGMYGDHHFAAAGYASYSNNAAAGNHLL

>SiDof10

MADTSLLCFGENHRKPAIHTYASRDMLPHVELPVRAAAACTGAAGIKLFGKVITTQQQPLPAPPMHAGGNAPPRLQNQAPPAPGRGSADLLEEAARARAAAAEARLPCPRCRSRDTKFCYFNNYNVNQPRHFCRACHRYWTAGGAIRNVPVGSGRRKNRPVLPPMPPPHAATNTGADHASSGSGSPPVFAASGLAVPYRGSPFHLAPPSPACTAASGLPEATTGQYWWLVAGGAVASSDRAF

>SiDof11

MHHRDIVVVTRMLSPHYDNVLPPYAAGRRAAALLDHRRYRPNVEVAPNCPRCDSPNTKFCYYNNYSLSQPRYFCKGCRRYWTKGGSLRNVPVGGGCRKNRRGKPVRAMPVDAAATGTAGGATAPYYQRSSSFPGTLRPDLLLEGMVGSPAGLCQPMEAAADKPAVVEGSTIDLALLYAKFLNHQPPPAVEPCAILPESLDALRGSSSDMSPDVPPPPDHHPFTRQDAFGELSTPASADPGAAAPQCPDARTEVLAELGFSVDQSCYDSLGLSTDDGDLILPSTWQPEAKYEPFDPLPEDAMSLHGGISGGDDVWSSALACQGLEAALCRP

>SiDof12

MVFSSLPIFLDPPNWGQMQMQQQQPPLQCLLGGGGGGSDHHHLMPPPSGLAPLPGGPADTAASAPAGGGSSTSMQAAAGAAAQPRPVVSMAERARLARVPLPEPGTLRCPRCDSTNTKFCYFNNYSLSQPRHFCKACRRYWTRGGALRNVPVGGGCRRNTKRSSKKSSRGGGAGATAATSSSSTTSTSTTATTTTATTSAAMAAAEAIASMQAQLPHLGLPPAAAAAALEASLEGYHHYLPLQMQPQFLQQAGLHGYHFADDGSGVLADGFPRGVVASGLLAQLAAVKMEEHGGGGGGGGAVAAHEQPYWAGSNGGGSGWPAEFLSGFSSSSSGNVL

>SiDof13

MEAPLHQSPVPLPLPPPQEEAVIGAALLRQAEAARAMMAAAAAAQHHQQQQPPAAAAMGREQCPRCASRDTKFCYYNNYNTAQPRHFCRACRRYWTLGGSIRNVPVGGSTRKRPRPARPTRALAAATATTTPASSAPFDTSSSSSPVAPAAALQGGLLGSLLLGSASASTLLALGAAPLLEGRLGFGPGLGQPALLAGANAAAGDLSRLDFGGAGPLLWPAAATVLEGDRAFPLPPPAAALWQKELAAAAPPPVEAGGLHHGGSPHLLL

>SiDof14

MQELQPIPGLAGRLFGSGGGAAAAIGLLRRHGGSSAAEVRCPRCDSPDTKFCYYNNYNLAQPRHFCRACRRYWTKGGHLRNVPVGGGCRKPRPRRPAAAAADGRGKDGVHRDGKAPRSGFAGAASSSSPTAAGDADAPVSGAFSVVTEPSAPRSGGVAEASAETGSFAAGDTRALLVPPPAPMFADQASVFASLFAPPRPLSAFGSSAQPQPEQAEERVAASLLAAEQPPPSCTAAFTDTAPFAAGSDGELSAGPSDWPTAGIFELAGGNAGDASLPEHWNHGSWTDPDPAVYLP

>SiDof15

MSDQKDPGIKLFGRVIPLAPEPAPEATETEDPLGHDQPPEELQPRAPEEEAAAVADEGQHNEKEDTEDSEMEVDTPQEKGSEMKVDTPLEKGNEMKVDTPLEKGNEMKIDTPQEEKGNEMKFDASKKEQDGEMKVDAQQEKKDEQMKVDAPPRAENIQPGTSPRSDHKKEDQGQMNSTEDKAASDPKGESEKANDESGQDKTLKKPDKILPCPRCNSMDTKFCYYNNYNVNQPRHFCKNCQRYWTAGGTMRNVPVGAGRRKSKNAPLHYRQLLMAPDCMLGSRVDISKSVLPEALVSPPSAPVQPTSRNETVLKFGPEVPLCESLVSALNIDEQNAKNPGSAPRGENREDNSCASAVTSYNGLPENTVHVDKNGAPVYCNGVAPVPQYYLGTPFMYPWSVGWNNLPVMVPGKSMPEAASASESCSTSSAPWMTSPMMPASTSPAFPYPLVPPALWGCLSGWPATTWNIPWVRTNGCVSPSSSSNSSCSGNGSPTLGKHSRDSNPLKDEKQEKSLWVPKTLRIDDPDEAAKSSIWATLGIKPGDPGTFKPFQSKVESKGQKADAAQVLQANPAALSRSQSFQESS

>SiDof16

MVSCAASSPSSAADMEAGQAADGGALAVVASVAAATGGRPGVAGGGREPEGLPCPRCESVNTKFCYYNNYNLSQPRHFCRACRRYWTRGGALRNVPVGGGTRKATPATRRKRTAGGTPPAPAPAPLVAALPPLTMSGPHGALLRQYGGLPFPAPALASPLAAVDPDRRLLDLGGSFSSLIAAAPPLDVGAHFSAGFLVGGLAPALAHSPAAAAALPPPPPPPQQVPQALPEGLIWSMGWPDLSI

>SiDof17

MANLPDADAAGFKLFGKVIQPPDAHRAAEEGAGAPPPPPPPPQPPTPTPTMAVLPPAPPQSPPPPPPPLQLQAAGAGAAAGGTGDPLPCPRCGSRETKFCYFNNYNVRQPRHLCRSCRRYWTAGGALRRVASASPGRRRPRPTARSAAAIATASSASAAAAEEGGGER

>SiDof18

MLSHVEMAPAAGGFKLFGKVITQCAESAPPVAAVPPQEAAASRSTAFARERDDPDERDQPMVKREAAADHDELVEKQQQDSGGGGAVESGESKGQQHPRPRQQQHQDTAEARAAAAASSAPPLPCPRCRSRNTKFCYFNNYNVNQPRHFCKDCHRYWTAGGALRNVPVGAGRRKNRPLGPVVAAGAVPTHHLHHQHRAAAVAAAAAAAGAGFVLGFPGQHPSSPTSPSPIYADRWPVCPDRRF

>SiDof19

MLSSHHEAMLPYAPRPPSLLVDRRYKQGGETAPNCPRCDSPNTKFCYYNNYSLSQPRYFCKGCRRYWTKGGSLRNVPVGGGCRKNRRGKSSARSAAESMASGRDAAAFGHRFPGPVRPDLVLEGMVGNPANPGQAIPDMPAAADGSTIDLAMLYAKFLNHPAAEAGAGAVTPESAGQVDEAFDTFSASSDLSPGVLAPAQFDPCHDGFGEWLGPVSSTDPTSTAASTSSATTMLCSDVSVQAALGELNFAMDQSCFDSLGLPTDVGNLSSWCSIVPSLSTLEDTKYDSLDSFPDDALSLHEGMISGPDNHDWSVDCQGLEALYMP

>SiDof20

MQGAAAEPGRRLAQQQFAGVDLRRPKGYAVPAPAEGDPCPRCESRDTKFCYYNNYNTSQPRHFCKGCRRYWTKGGTLRNVPVGGGTRKKPSSSPPSSYAAAANANKPKKPSKKKRRVVVVAPQDPVAAPVAAPTLVAAPAADSAKTTTNETTTTTTNETTATTATPTTTDAASEITTELVVPAAEEEDSLAHLLQPDVALGLGTSDFPAAGKALDLEPDSFEWPAAFDLGGACWGSAGFADPDPAGLFLNLP

>SiDof21

MCSRSSSGSTTATHNPTRAQTKSLGLVKPMEEMLMAANAGAAAANTGQGSNPNPPAPAPSSVPGGGALRAGAPPAVAGAGSTERRARPQKEKALNCPRCNSTNTKFCYYNNYSLQQPRYFCKTCRRYWTEGGSLRNVPVGGGSRKNKRSSSSSASASTSASVTSSSMASTAGAASKNPKLAHEGAQDLNLAFPHHSGLHAPEFGAFPSLESSSVCNSGGAMASNGRGGGGGGAGPAVGALSAMELLRSSGCYMPLQMPMHMPGDYAAGGFALGEFRAPPPSQSVLGFSLDAHGPGPGAAAAGYGSSAGLQGVPENAGRLLFPFEDLKPPVSSGGGGVATGASGGDGDGNSGHPQFDHNKDQGGGAPSAGHDPPGFWNGMIGGSGASW

>SiDof22

MDKIFNTTCNTTMPSTLQNQQEEAAIPPPAAARNVKKAKQQQAGGERMPRPQQEQALNCPRCHSTNTKFCYYNNYSVTQPRYLCKACRRYWTKGGTLRNVPVGGRCRKNKQQNPSAPSALASSSDSKKIMNSSTQQLLMMMPPPPPPTAANLSNVLPTTFMSATGVGGGFELPSSDHHPLPFAPLSLPSNPGTTPPASSFLDLLPFLPTPSSFGAMMLQHGPGMIAGAGGGLQQQWLPSSQHGNDDGGLFAAGGSPAAAAVQEPQQQQEEEVGGGDGGTAADAAGNDDMGGGGASADIINIYWSSSRI

>SiDof23

MVASPPREEAAAARNIKANQAWQQQVAATGSGERKPRPQQEQGLNCPRCNSTNTKFCYYNNNSMTQPRYFCKACRRNWTQGGTLRNVPVGGRSRKNKLNRAGGSSSSSTLPAPSSSSNDSKKMNLTQQLSMMPTATAPMPADFPNVLPTFMSTGGGFELPSSDHHSLPFPPLSPLFNPGTTPSLLDILTGGFLDSGNNRMAAPPLLGTMQHGHGMVGGSPDPQLVGLLQGVDQALELPMPAAGGGFQQWPSLAAQEQQVVGGDGSADMDDNNNNNNGGGASGGSSGVEYYWQGSI

>SiDof24

MVASPPREEADPARNVKAKQAQQQQVAASGASERKPRPQQEQGLNCPRCNSTNTKFCYYNNNSMTQPRYFCKACRRNWTQGGTLRNIPVGGGSRKNKQNRAGSSSSSSPAPPAPSSSSTNSKKMNLTQQLLMMPTAMAPMPADFPIVLPTFMSTGGGGFELPSSDHHSLPFPPLSLPSNPGTTPSLLDMLTGGFLDGGIGALPFLPTPPSFGAMQHGHGIMVGSHDQQLGVDQALKPPMAATGGSGLQQWPSSAAQDQHGSI

>SiDof25

MVASPSREEAAAARNIKAKQERQQQVVASGADERKPRPQQEQGLNCPRCNSTNTKFCYYNNNSMTQPRYFCKACRRNWTQGGTLRNVPVGGGSRKNKKNRAGGSSSSSSAPPAPSSSCTNSKKMNLTQQLLMMPTAMAPMPADFPNVLPTFMSAGGEGFELPGSDHHSVPFPPLSLPSNPGTMPSLLNMLTGGFLDGGMGAQPFLPAPPSFGARQHEHAIICGSSDQQVVGPLQGVDKALKPPMAAAGGSGLQQWPSSAAQELQVVGGDGSAFNNNNGNNNGGGASGGISGVEHYWHGST

>SiDof26

MVASPPREEAAAARNVKAKQAWQQQMAASGGGERKPRPQQEQGLNCPRCDSTNTKFCYYNNNSMTQPRYFCKACRRNWTQGGTLRKVPIGGSSRKNKQSRAGGSSSSSSSAPPAPSSTSNDSNKMNLTQQLMMMPTTTTPMPANFPNVLPTFMSAGSGSFGELPGSDHHSLPFPPLSLPSNPTGTMPSLVGILRGGFPDGGMAALPFLPVPPSFGAMHQHGHGMMGGSSDQQLVGPLQGMDQALKLPLAAACGSGPQQWPSSAAPEQQVVGGDGRADKNNNNNGGGATGSSSGVEYYWQGSI

>SiDof27

MVFSSVPVYLDPPNWNQHQHQQQQQQAHHGQLPSGGGGVETHAHHHQQHQHHQQHQHQHHQLPPMPPPGALMAPRPDMETIAVAASGGGGGGGGPTGGSAVRPGSMTERARLAKIPQPEPGLKCPRCESTNTKFCYFNNYSLSQPRHFCKTCRRYWTRGGALRNVPVGGGCRRNKRTKSSKSSSSSAAASASATGGGTSSSTSSTATGGSSAAAAVMPPQGQLPFLASLHHPLGGDHYSTGASRLGFPGLSSLDPVDYQLGGAGGGGGTAIGFEQWRLPQIQQFPFLSRPDAVPPPMSGIYPFDVEGHGDPAGFAGQMLGGSKVPGSAGLITQLASVKMEDNPPSTAMTSLPREFLGLPGNLQFWGGGGNGGASGNNGGTANTGGGGGGSGASAPGSSWVDLSGFNSSSSGNIL

>SiDof28

MACPFSSPSSNFFISHTPVPLPHPTTHPLIHTTKQEPESNHTTPPLPSPRKNPSPSASRSLFELEFSPSITMIQELLGGAAMDQLKSVNDGNPASLPMLLHPTVSNPSPTSSSSTSSRSSAQQPHQQQRSTSATSSPQGQQQVQGAEQAPLRCPRCNSSNTKFCYYNNYNLTQPRHFCKTCRRYWTKGGALRNVPIGGGCRKPRPMPTPVAKPALSCKAVGGAPSLGLGVGLGMGAGPVPWASSQQAAAAQLMALLNSARGVQGGGGHGGSNMHRLLGLDSMAHLPIHVLPGAGNASGAPASLWPPAASRPIPTPPPHVDSQLGMGPLGQHDVLSTLGLKLPSPSPSLAASYYSDQLHAVVSSAAGRPHEYDAPGTTSLPCTTAAASLPPPASSVSAALSSATVGLDLPPISLPAPEMQYWAGPAAMSMAWPDLPTPNGAFP

>SiDof29

MAGQVMEAHAHHQARLQPPAVPPFAPLPPSCKNDVHHFPAAMATPGSNAAAAADMAAYLQQLQDTAAAAAEANKSSSGGNGGGAARGEQCPRCASHDTKFCYYNNYNTSQPRHFCRACRRYWTLGGSLRNVPIGGSTRKRPRLAHHHHHHQQQPAAARRAPVFGLGLGGGGGAHPPMPSSSSSQAGGLLGSLFALGAAAPLLDGPAAPMLEGRGAGLFDLGLGLPVPGGAVGGAGDAGVQMQGLGLIRGGGGGHAGGASSGLFWPAGLLDSDSVDTWKMLPGVGAGAMWPEFSAAAAPAPQAGGLLHGGAQLM

>SiDof30

MASSTPVAAGDDAVGTRKGGTGGSGGAAPPPPATQQQQPPPPPEQGLRCPRCDSPNTKFCYYNNYSLSQPRHFCKTCRRYWTKGGALRNVPVGGGCRKNKRSRSAAAAAAASRLSLNLPVEGVGGDQQAARLGFLGATGGAPVASSPIGGGGPAADYQQAAGGAVGMMALPRLHALGVGQYVPFGEWPSGAGGDISGGGGGGHAMSGGGAHGGAVSSNIASSIESLSFINQDLHWKLQQQRLATMFLGPPPPPPPTSSASHIDGAPAAAPAHIGGAFLQMAGPPGMESTMPAATSWFMDSSYAVLPSPHAHANNTAAAAITAAATTTNCNVGRSSGGDDDDAANCGSAIPSWGDMSTFAMLP

>SiDof31

MPSSFLASASSSASSPLSYLIPAASRPPPPPLVMGQGYSVDGLAGVPTGGVVVDATSAAAVAPPRPGARNPGHPPLPRPPPRQCPRCQSANTKFCYYNNYSRTQPRYLCKACRRHWTEGGTLRDVPVGGGRKNRRGGSKAGAAAAKASASASTSAAAAAATQGGSVAVGADTFPDLLRQLMQFQPTAPAGGGYAIDLSAWQQMAAATAPPQGAGDVSALGGAAAAAAEANCGALQYWAGWQPDDMPGLDGPC

>SiDof32

MMAECRGGDCLIKLFGKTIPVPEAADAAKESGSSSSSTESDAPENAHQEASDPSPQPEVVDAEDPKSSPETTQQPDAAGDVASQREKLKKPDKVLPCPRCNSMDTKFCYFNNYNVNQPRHFCKNCQRYWTAGGAMRNVPVGAGRRKNKHAVAASHFLQRVRAALPAAGDPLKTNGTVLSFGGHGPPPALHDLTEQVNHLQEKLLIPARKTSNPSPAVGPCSEGSSSTDDMTHGGGIKEKSSTVDKPANGAQYPAGMNGAAVWPYSCAPPPAYFSSGIAIPIYPAAPGYWGCMVPGAWSLPWPVQPPSQGLSSPTSAPSVSSSGPDSLTLGKHPREVDEGRSSAHGSGKVWAPKTIRIDDADEVARSSIWSLIGIKGDKKRDDADHAVGHKHGTVFEPKLEVNKAAKPGMITRSPFLHTNPVALTRSVTFQEGS

>SiDof33

MMHDMMRRKQTTKEDQTFSTMPYHGRIPTHGGGGTGSGSKEAHRYTQHQLPRLPGAPMAPHLSDTPAEASASADDGSAGGSCWVKPGCMMELARLAKIPQPASGLMCPRCRSTETKFCYYNNYSLSQPRHFCKTCRRYWTHGGALRDLPFSSSVRRRRRNKPSNNKQTSSKVACCGAFSGSTGMSPSSSPSSGATIFSGRVATAATAILKPLEQLLIGGAEHHRAGASRLWFPGHSSQDPVPLGYRHQLGNSRGAATTIRLEHQRYLPQRQPFSLLGYKNGGTAPATSAICPFSEGARGA

EAASFAGQMHAAISRARGSAAVTTTELASEMMAGNPPIPSTEMGTLASSPGEFLVGVQGDHDLFHFLGSGSWACGYGSTAGNNGSGGGSSCTAAPGSAWPDPSGFTSSSSGRSTIL

>SiDof34

MVFPSVPAYLDPPNWNNQQGQQPRASAGGGDAPLLPVGPAAATAAGPDNSGLPSSSSTASAAVAAQARPNSMAERARLARMPQPEQALKCPRCDSTNTKFCYYNNYSLSQPRHFCKACRRYWTRGGSLRNVPVGGGCRRNKRSSKSSGGSSSSKPYSSARQLAGPSSSTPSSTPGATGAIIPPSLGSFSHHLPFLGTMHQPGPNLGLAFSAGLPPLGMQHMDTVDQFPVASGGGATIGASLEQWRVQQQPQQQFPFLTGGGILELPPPAMYQLGLDGNNRGGSGSAAAAAFTLGQTSATT

ARQEGSMKVEGSKGQDMSLQRQYMAALRHGSQGVWDGIHGNAGSSGGDGGGNGGSSWPMNIPGFHSSSTGGGNGSGL

>SiDof35

MAECRVGGGGGDGLIKLFGKTIPVPEAAAAVGEADKDIQQSGSSTTELKGQENTLQDSTGSPPQQEVADTEDSSATKKSSGDQQQGETTNQKEKLKKPDKILPCPRCNSMDTKFCYYNNYNINQPRHFCKNCQRYWTAGGAMRNVPVGAGRRKSKSASATSHFLQRVRAALPIDALCAAAKTNGTVLSFGSDMSSLDLTEQMKHLKEKLVPITRIKSSDDRSAGSCTEGSAKVEDSNQKEKVTADKSANVVPHPCMNGVTMWPFSCAPPPACYTSGIAIPFYPAAAAYWGCMVPGAWNTPWPPHSQSESASPLSTASPASTKSNCFTPGKRSRDCDEEGDTKGHGKVWVPKTIRIDDADEVARSSILSLIGINGDKAGKDGRGCKLAKVFEQQKEEAKTATHAVINSLPFLQGNPAALSRSLTFQEGS
