# Supplementary material for: Herbicide Metabolic Resistance in Poaceae Plants via the GA‐GID1/DELLA‐DOF2‐P450s Module
Source: Adv Sci (Weinh). 2026 Jul 24;13(57):e76742. doi: 10.1002/advs.76742 (PMC13398137; doi:10.1002/advs.76742)
Supplement: Supplementary file 3 [file ADVS-13-e76742-s001.zip › Supplemental Data S2.docx]

**Supplemental Data S2.** The full-length coding sequences and protein sequences for EcDOF2, EcDELLA, OsDOF2, and OsDELLA.

>EcDOF2 Scaffold259.96 length=1710 bp (coding sequence)

ATGTCGGATCAGAAGGACCCTGGGATCAAGCTCTTCGGCCGGGTGATCCCGCTAGCTCCTGACCCCGCGCCGGGGACCATGGAAACGGAGGAACCGCCATGCGACGACCAGCCGCACGAGGAGCTGCAGCCACGGGCGGCGGCGGCGGCGGAAAAGGATCAACATAATGAAAAAGAAGAGAAAGAAGATAGTGGAATGAAGGTTGACACGCCACAAGAGAAGGGCAATGAAATGAAGGCTGACACAACACAAGAGGAGGGCAATGAAATGAAGGTAGACACACCACAAGAAAAGGGTGATGAAATGAAGTTTGATGCACCACAAAAGGAACAAGATGATGAAATGAAAGTTGATGCACAGCAAGAGAAAAGAGATGAGCAAATGAAAATCGATGCACCGCCAATGACAGAAAATATACAACCAGGCAGTTTACCTTCCTTGGACTCGGACCATAAGAAAGAAGATCAGGGCCAGACGAACAGTACTGAAGATAAAGCGGCGTCAGACCCAAAGGGGGAGAATGAGAAGCCATCAAATGATGAATCAGGCCAGGATAAGACACTTAAGAAGCCAGATAAGATTCTACCTTGTCCTCGGTGCAACAGCATGGATACAAAGTTCTGTTATTACAACAACTACAATGTTAATCAACCTAGGCACTTTTGTAAGAATTGCCAGCGGTACTGGACTGCAGGGGGTACTATGAGGAATGTACCTGTTGGTGCTGGACGACGCAAAAGCAAGAATGCATCATTACACTACCGCCAACTACTGATGGCCCCTGATTGTATGCTGGGGTCTAGAGTGGACATGTCGAAGTCAGTGCTCCCTGAAGCTCTTGTATCTCCTGCCCCAATACAGCCAACCAGTCGCAATGAAACAGTTCTCAAATTTGGGCCTGAGGTGCCACTTTGTGAATCAATGGTGTCAGCACTGAACATCGATGAGCAGAATGTAAAGAACCCTGGATCAGCAACAGGAGGTGAAAATGGGGAGGACAACTCTTGTGCATCTTCTGTCACATCATACAATGGACTGCCTGAAAACGCGGTTCACGCAGATAAGAATGGAGCGCCCATATATTGTAACGGAGTTGGACCAGTGCCTCAATATTACCTTGGAACCCCTTTCATGTACCCTTGGAGTGTAGGATGGAACAATCTCCCTGTGATGGTGCCAGGTAAGGGTATGCCTGAATCTGCTTCTGCTTCTGAAGGCTGCAGTACTAGTTCAGCTCCATGGATGACCTCTCCCATGATGCCGTCCTCAAGACTTCCTGGACCAGCATTTCTGTACCCCCTTGTGCCACCCGCGCTTTGGGGTTGTTTGTCTGGATGGCCAGCCACGACGTGGAACATACCATGGATTAGCGCAAATGGTTGTGTCTCCCCGTCATCATCAAGCAACAGCAGCTGCTCAGGCAATGGTTCTCCAACTCTGGGTAAACATTCCAGGGACTCTAATCCACTGAACGAGGAGAAGAAAGAGAAATCATTGTGGGTTCCCAAGACACTCCGGATCGATGACCCTGATGAGGCTGCAAAAAGTTCAATATGGGCCACCCTTGGCATCAAACCCGGAGACCCTGGCACCTTCAAGCCTTTCCAGTCCAAGGTTGAGAGCAAGGGCCAGAAATCAGATGCTGCTCAGGTTTTGAAGGCGAATCCAGCAGCATTATCACGCTCACAATCATTCCAGGAGAGCTCTTGA

>EcDOF2 Scaffold259.96 length=569 aa (protein sequence)

MSDQKDPGIKLFGRVIPLAPDPAPGTMETEEPPCDDQPHEELQPRAAAAAEKDQHNEKEEKEDSGMKVDTPQEKGNEMKADTTQEEGNEMKVDTPQEKGDEMKFDAPQKEQDDEMKVDAQQEKRDEQMKIDAPPMTENIQPGSLPSLDSDHKKEDQGQTNSTEDKAASDPKGENEKPSNDESGQDKTLKKPDKILPCPRCNSMDTKFCYYNNYNVNQPRHFCKNCQRYWTAGGTMRNVPVGAGRRKSKNASLHYRQLLMAPDCMLGSRVDMSKSVLPEALVSPAPIQPTSRNETVLKFGPEVPLCESMVSALNIDEQNVKNPGSATGGENGEDNSCASSVTSYNGLPENAVHADKNGAPIYCNGVGPVPQYYLGTPFMYPWSVGWNNLPVMVPGKGMPESASASEGCSTSSAPWMTSPMMPSSRLPGPAFLYPLVPPALWGCLSGWPATTWNIPWISANGCVSPSSSSNSSCSGNGSPTLGKHSRDSNPLNEEKKEKSLWVPKTLRIDDPDEAAKSSIWATLGIKPGDPGTFKPFQSKVESKGQKSDAAQVLKANPAALSRSQSFQESS

>EcDELLA scaffold20.707 length=1878 bp (coding sequence)

ATGAAGCGCGAGTACCAAGACGCCGGCGGGAGCGGCGGCGGCGACATGGGCTCCTCCAAGGACAAGATGACGGCGGCGGCGGGGGCGGGGGAGCAGGAGGAGGACATGGACGAGCTGCTGGCCGCGCTCGGGTACAAGGTGCGCTCGTCGGACATGGCGGACGTCGCGCAGAAGCTGGAGCAGCTCGAGATGGCCATGGGGATGGGCGGCGTCGGCGGCGCCGGCGCCGCCGCCGATGACGGGTTCGTCTCGCACCTCGCCACGGACACGGTGCACTACAACCCCTCCGACCTTTCGTCCTGGGTCGAGAGCATGCTGTCCGAGCTCAACGCGCCCCCGCCGCCGCTCCCGCCCGCGCCGCCGGCACCGCGGCTCCCGTCCACCTCGTCCACCGTCACGGGCAGCGCCGCCGCCGGTGCGGGCTACATCGATCTCCCGCCCGCCGTCGACTCGTCCAGCAGCACCTACGCTCTCAAGCCGATCCCATCGCCGGTCCCGGCGTCGGCCGACCCGTCAATGGACTCGGCGCGGGAGCCCAAGCGGATGCGAACTGGCGGCGGCAGCACGTCGTCTTCCTCTTCCTCGTCGTCGTCCCTGGACGGCGGCCGCACCAGGAGCTCCGTGGTCGAGGCGGCCCCGCCGGCGACGAAGGCATCCGCGGCGGCCAACGGGCCCGCGGTGCCGGTGGTGGTGGTGGACACGCAGGAGGCCGGGATCCGGCTCGTCCACGCGCTGCTGGCGTGCGCGGAGGCCGTGCAGCAGGAGAACTTCACGGCGGCGGAGGCGCTGGTCAAGCAAATCCCCATGCTGGCCTCGTCGCAGGGCGGCGCCATGCGCAAGGTCGCCGCCTACTTCGGCGAGGCGCTCGCTCGCCGCGTCTACCGCTTCCGCCCTGCCCCGGACAGCTCCCTCCTCGACGCCGCCTTCGCTGACCTCCTCCACGCCCACTTCTACGAGTCCTGCCCCTACCTCAAGTTCGCCCACTTCACCGCGAACCAGGCCATCCTCGAGGCGTTCGCCGGCTGCCGCCGCGTCCACGTCGTCGACTTCGGCATCAAGCAGGGGATGCAGTGGCCGGCTCTCCTCCAGGCCCTCGCCCTCCGCCCCGGCGGCCCCCCATCGTTCCGCCTCACTGGCGTCGGCCCGCCGCAGCCGGACGAGACCGACGCCTTGCAGCAGGTGGGTTGGAAGCTCGCCCAGTTCGCGCACACCATCCGCGTCGACTTCCAGTACCGGGGGCTCGTCGCCGCCACGCTCGCTGACCTGGAGCCCTTCATGCTGCAACCGGAGGGCGGCGAGGACACGGATGACGAGCCCGAGGTGATCGCCGTCAACTCGGTGTTCGAGCTGCACCGGCTGCTGGCGCAGCCCGGCGCCCTGGAGAAGGTCCTGGGCACGGTGCGCGCAGTGCGGCCGAGGATCGTGACCGTGGTCGAGCAGGAGGCCAACCACAACTCCGGCTCATTCCTGGACCGCTTCACGGAGTCGCTGCACTACTACTCCACCATGTTCGATTCCCTCGAGGGCGCCGGCTCAGGCCAATCCGCCGCTGATGCCTCCCCGGCCGCGGCCGGCGGCACGGACCAGGTCATGTCCGAGGTGTACCTCGGCCGGCAGATCTGCAACGTCGTGGCGTGCGAGGGCGCGGAGCGCACGGAGCGCCACGAGACGCTGGGGCAGTGGCGCAACCGCCTCGGCCGCGCCGGCTTCGAGCCCGTGCACCTGGGCTCCAATGCCTACAAGCAGGCGAGCACGCTGCTGGCGCTCTTCGCCGGCGGCGACGGGTACAGGGTGGAGGAGAAGGAAGGGTGCCTGACCCTCGGGTGGCACACGCGCCCGCTCATCGCCACCTCGGCATGGCGCATCACCGCGCCGTGA

>EcDELLA scaffold20.707 length=625 aa (protein sequence)

MKREYQDAGGSGGGDMGSSKDKMTAAAGAGEQEEDMDELLAALGYKVRSSDMADVAQKLEQLEMAMGMGGVGGAGAAADDGFVSHLATDTVHYNPSDLSSWVESMLSELNAPPPPLPPAPPAPRLPSTSSTVTGSAAAGAGYIDLPPAVDSSSSTYALKPIPSPVPASADPSMDSAREPKRMRTGGGSTSSSSSSSSSLDGGRTRSSVVEAAPPATKASAAANGPAVPVVVVDTQEAGIRLVHALLACAEAVQQENFTAAEALVKQIPMLASSQGGAMRKVAAYFGEALARRVYRFRPAPDSSLLDAAFADLLHAHFYESCPYLKFAHFTANQAILEAFAGCRRVHVVDFGIKQGMQWPALLQALALRPGGPPSFRLTGVGPPQPDETDALQQVGWKLAQFAHTIRVDFQYRGLVAATLADLEPFMLQPEGGEDTDDEPEVIAVNSVFELHRLLAQPGALEKVLGTVRAVRPRIVTVVEQEANHNSGSFLDRFTESLHYYSTMFDSLEGAGSGQSAADASPAAAGGTDQVMSEVYLGRQICNVVACEGAERTERHETLGQWRNRLGRAGFEPVHLGSNAYKQASTLLALFAGGDGYRVEEKEGCLTLGWHTRPLIATSAWRITAP

>OsDOF2 LOC_Os01g15900 length=1656 bp (coding sequence)

ATGTGTGACAAGGATCCGGGCATAAAGCTCTTCGGGCGGGTGATCCCGCTGGCTCCAGAGGCTGAGGCTGCTGCGGCGGCGGACGGCTCCGACCAGCCGGAGGCGGCGGCGGCGGCGGCGGCGGAGGTTGAGCCGGCGGCGCAGGATGAGGATCATCACAAGGAAACAGAAGAAAGAAAATATGATGAAATGAAGGTTGATGTGCCACAAGAGGAGGAAGATAACGAAATGAAGGTCGATGCACCACAAGAGAAAAAGGATAATGAAGTGACGGCTGATGTGCCAGAAGAGAAGGGAAATGATGAAATGAGGGTTGATGCATCAGAGTCAATAGAAAGCATAGAGCCAGTCAGCAGATCTACCTTGGACAATAAAAAAGAAGATCAGGGTCAGATGAACAACGTTGAAGAGAAAGCAGCATCAGACTCAAAAGATGAAAATGAAAAGACAGCAAATGATGAATCAGGCCAGGACAAAGTACTTAAGAAGCCAGATAAGATTCTCCCTTGCCCTCGGTGCAACAGTATGGACACAAAGTTTTGTTATTACAACAACTACAATGTTAATCAACCCAGGCACTTCTGTAAGAACTGCCAAAGGTATTGGACTGCCGGGGGAACCATGAGAAATGTACCTGTTGGTGCTGGGAGGCGCAAAAGCAAGAGCTCATCGTTGCACTACCGTCACTTACTGATGGCCCCTGATTGCATGATGGGGTCTAGAGTGGAAATATCCAAGTCAATGAACCCTGAAGCTTTCGCATCTGCGCATTCGACCCCTATACAACCAATTGGCAGAAACGAAACAGTTCTCAAATTTGGGCCTGAGGTGCCACTCTGTGAATCGATGGCATCAGTGCTGAACATTCAGGAGCAGAATGGAACCAATGCTGCAGCAGTACCAACGGGTGAAAATCAGGAAGATAACTCTTGCATCTCTTCAATCACATCACACAACGTGTTACCTGAAAATGCAGCCCAAGTTGACAAGAACAGCACGCCGGTGTATTGCAACGGAGTCGGCCCAGTGCCGCAGTACTACCTTGGAGCTCCTTACATGTACCCATGGAACATAGGATGGAACAACGTTCCTATGATGGTGCCAGGTACAAGCATGCCAGAGTCTGCTTCCCAATCTGAGAGCTGCAGCACCAGTTCAGCTCCATGGATGAACATGAACTCCCCCATGATGCCGGTTGCCTCGAGGCTTTCTGCACCACCATTTCCATACCCTCTAGTGCCACCTGCACTATGGGGTTGCTTATCCAGCTGGCCGGCCACGGCATGGAACATACCGTGGATCAGAACGAATGGCGGCTGCATGTCTCCATCGTCGTCGAGCAACAGCAGCTGTTCAGGCAATGGCTCCCCTCTGGGGAAGCATTCCAGGGACTCCTCTCTCCCACTGAAGGAGGACAAGGAGGAGAAATCACTGTGGGTTCCCAAGACGCTCCGCATCGACGATCCCGACGAGGCGGCGAAGAGCTCCATCTGGGCCACCCTGGGGATCAAGCCTGGAGACCCTGGCATCTTCAAGCCGTTCCAGTCCAAAGGTGAGAGCAAAGGCCAAGCAGCATCAGAGACTCGTCCTGCTCGTGCTCTTAAGGCAAACCCAGCTGCATTGTCGCGGTCGCAGTCGTTCCAGGAGACTTCTTGA

>OsDOF2 LOC_Os01g15900 length=551 aa (protein sequence)

MCDKDPGIKLFGRVIPLAPEAEAAAAADGSDQPEAAAAAAAEVEPAAQDEDHHKETEERKYDEMKVDVPQEEEDNEMKVDAPQEKKDNEVTADVPEEKGNDEMRVDASESIESIEPVSRSTLDNKKEDQGQMNNVEEKAASDSKDENEKTANDESGQDKVLKKPDKILPCPRCNSMDTKFCYYNNYNVNQPRHFCKNCQRYWTAGGTMRNVPVGAGRRKSKSSSLHYRHLLMAPDCMMGSRVEISKSMNPEAFASAHSTPIQPIGRNETVLKFGPEVPLCESMASVLNIQEQNGTNAAAVPTGENQEDNSCISSITSHNVLPENAAQVDKNSTPVYCNGVGPVPQYYLGAPYMYPWNIGWNNVPMMVPGTSMPESASQSESCSTSSAPWMNMNSPMMPVASRLSAPPFPYPLVPPALWGCLSSWPATAWNIPWIRTNGGCMSPSSSSNSSCSGNGSPLGKHSRDSSLPLKEDKEEKSLWVPKTLRIDDPDEAAKSSIWATLGIKPGDPGIFKPFQSKGESKGQAASETRPARALKANPAALSRSQSFQETS

>OsDELLA LOC_Os03g49990 length=1878 bp (coding sequence)

ATGAAGCGCGAGTACCAAGAAGCCGGCGGGAGCAGCGGCGGCGGGAGCAGCGCCGATATGGGGTCGTGCAAGGACAAGGTGATGGCGGGGGCGGCGGGGGAGGAGGAGGACGTCGACGAGCTGCTGGCGGCGCTCGGGTACAAGGTGCGGTCGTCCGACATGGCCGACGTCGCGCAGAAGCTGGAGCAGCTGGAGATGGCCATGGGGATGGGCGGCGTGAGCGCCCCCGGCGCCGCGGATGACGGGTTCGTGTCGCACCTGGCCACGGACACCGTGCACTACAACCCCTCGGACCTCTCCTCCTGGGTCGAGAGCATGCTTTCCGAGCTCAACGCGCCGCTGCCCCCTATCCCGCCAGCGCCGCCGGCTGCCCGCCATGCTTCCACCTCGTCCACTGTCACCGGCGGCGGTGGTAGCGGCTTCTTTGAACTCCCAGCCGCTGCCGACTCGTCGAGTAGCACCTACGCCCTCAGGCCGATCTCCTTACCGGTGGTGGCGACGGCTGACCCGTCGGCTGCTGACTCGGCGAGGGACACCAAGCGGATGCGCACTGGCGGCGGCAGCACGTCGTCGTCCTCATCGTCGTCTTCCTCTCTGGGCGGTGGGGCCTCGCGGGGCTCTGTGGTGGAGGCTGCTCCGCCGGCGACGCAAGGGGCCGCGGCGGCGAATGCGCCCGCCGTGCCGGTTGTGGTGGTTGACACGCAGGAGGCTGGGATCCGGCTGGTGCACGCGTTGCTGGCGTGCGCGGAGGCCGTGCAGCAGGAGAACTTCGCGGCCGCGGAGGCGCTGGTCAAGCAGATCCCCACGCTGGCCGCGTCCCAGGGCGGCGCCATGCGCAAGGTCGCTGCCTACTTCGGCGAGGCCCTCGCCCGCCGCGTGTACCGCTTCCGCCCCGCGGACAGCACCCTCCTCGACGCCGCCTTCGCCGACCTTCTGCACGCCCACTTCTACGAGTCCTGCCCCTACCTCAAGTTCGCCCACTTCACCGCAAATCAAGCCATCCTCGAGGCTTTCGCCGGCTGCCACCGCGTCCACGTCGTCGACTTCGGCATCAAGCAGGGGATGCAATGGCCAGCTCTCCTCCAGGCCCTCGCCCTTCGTCCCGGCGGCCCCCCATCGTTCCGCCTCACCGGCGTCGGCCCCCCGCAGCCGGACGAGACCGACGCCTTGCAGCAGGTGGGTTGGAAGCTTGCCCAGTTCGCGCACACCATTCGCGTCGACTTCCAGTACCGGGGACTCGTCGCCGCCACTCTCGCGGACTTGGAGCCGTTCATGCTGCAGCCGGAGGGCGAGGCGGACGCGAACGAGGAGCCTGAGGTGATCGCCGTCAACTCGGTGTTCGAGCTGCACCGGCTGCTCGCGCAGCCCGGCGCGCTGGAGAAGGTCCTGGGCACGGTGCACGCGGTGCGGCCAAGGATCGTCACCGTGGTAGAGCAGGAGGCCAACCACAACTCCGGCTCATTCCTCGACCGGTTCACCGAGTCGCTGCACTACTACTCCACCATGTTCGATTCCCTCGAGGGCGGCAGCTCCGGCCAGGCCGAGCTCTCTCCGCCGGCTGCCGGGGGCGGCGGTGGCACGGACCAGGTCATGTCCGAGGTGTACCTCGGCCGGCAGATCTGCAACGTCGTGGCGTGCGAGGGCGCGGAGCGCACGGAGCGCCACGAGACGCTGGGGCAGTGGCGCAACCGCCTCGGCCGCGCCGGCTTCGAGCCCGTGCACCTGGGCTCCAATGCCTACAAACAGGCGAGCACGCTCCTCGCGCTTTTCGCCGGCGGCGACGGCTACCGGGTGGAGGAGAAGGAGGGCTGCCTCACGCTGGGCTGGCACACGCGCCCGCTCATCGCCACCTCGGCATGGCGCGTCGCCGCGGCGTGA

>OsDELLA LOC_Os03g49990 length=625 aa (protein sequence)

MKREYQEAGGSSGGGSSADMGSCKDKVMAGAAGEEEDVDELLAALGYKVRSSDMADVAQKLEQLEMAMGMGGVSAPGAADDGFVSHLATDTVHYNPSDLSSWVESMLSELNAPLPPIPPAPPAARHASTSSTVTGGGGSGFFELPAAADSSSSTYALRPISLPVVATADPSAADSARDTKRMRTGGGSTSSSSSSSSSLGGGASRGSVVEAAPPATQGAAAANAPAVPVVVVDTQEAGIRLVHALLACAEAVQQENFAAAEALVKQIPTLAASQGGAMRKVAAYFGEALARRVYRFRPADSTLLDAAFADLLHAHFYESCPYLKFAHFTANQAILEAFAGCHRVHVVDFGIKQGMQWPALLQALALRPGGPPSFRLTGVGPPQPDETDALQQVGWKLAQFAHTIRVDFQYRGLVAATLADLEPFMLQPEGEADANEEPEVIAVNSVFELHRLLAQPGALEKVLGTVHAVRPRIVTVVEQEANHNSGSFLDRFTESLHYYSTMFDSLEGGSSGQAELSPPAAGGGGGTDQVMSEVYLGRQICNVVACEGAERTERHETLGQWRNRLGRAGFEPVHLGSNAYKQASTLLALFAGGDGYRVEEKEGCLTLGWHTRPLIATSAWRVAAA
