# Supplementary material for: Herbicide Metabolic Resistance in Poaceae Plants via the GA‐GID1/DELLA‐DOF2‐P450s Module
Source: Adv Sci (Weinh). 2026 Jul 24:e76742. Online ahead of print. doi: 10.1002/advs.76742 (PMC13398137; doi:10.1002/advs.76742)
Supplement: Supplementary file 3 — Supporting File 3: advs76742‐sup‐0003‐MethodS1.docx. [file ADVS-9999-e76742-s002.docx]

**Supplementary Data S5. Agrobacterium-mediated genetic transformation protocol for Echinochloa crus-galli**

1.**Selection and Surface Sterilization of *Echinochloa crus-galli* Embryos**

Healthy and plump seeds of barnyard grass (*Echinochloa crus-galli*) were selected. After removing the seed coat and glumes, the embryos were isolated and collected into sterile 5 mL centrifuge tubes. Surface sterilization was carried out in a laminar flow hood as follows: the embryos were gently inverted with 20% (v/v) sodium hypochlorite solution for 2 minutes, followed by 75% (v/v) ethanol for 5 minutes, and finally rinsed 5–7 times with sterile distilled water.

**2.Callus Induction**

The surface sterilization embryos were placed on sterile filter paper to remove excess moisture. A wound was made on each embryo using a scalpel blade, and the embryos were then placed on the surface of callus induction medium. The cultures were incubated at 28°C in the dark for 7 days. The germinating shoots were removed, and the explants were further cultured in the dark. Compact and embryogenic calli were selected for subculture and subsequent *Agrobacterium*-mediated transformation.

**3.*Agrobacterium* Infection**

An *Agrobacterium* strain verified as positive was streaked for activation. A single colony was picked and inoculated into 1 mL of LB liquid medium supplemented with 50 μg/mL kanamycin sulfate and 25 μg/mL rifampicin, and cultured overnight at 28°C with shaking at 200 rpm. The culture was then transferred at a 1:50 ratio into fresh medium and grown until the optical density at 600 nm (OD_600_) reached 0.6–0.8. The bacterial cells were harvested by centrifugation at 3500 rpm and resuspended in AAM liquid medium to an OD_600_ of 0.6. Compact and dense calli were selected and immersed in the resuspended *Agrobacterium* suspension. Infection was carried out at 28°C in the dark for 5 minutes. After infection, the calli were blotted dry on sterile filter paper to remove excess bacterial suspension and transferred to callus induction medium containing hygromycin for subculture and selection of positive calli.

**4.Callus Differentiation, Rooting, and Plantlet Regeneration**

Yellowish and compact calli were selected and transferred to differentiation medium under low-light conditions. After the calli formed green spots and differentiated into shoots, the plantlets were transferred to tissue culture bottles containing rooting medium. When the root system was well established, the plantlets were gently removed from the medium, placed in 15 mL centrifuge tubes with water covering the entire root system, acclimated for 2 days, and subsequently transplanted into soil.

**5.Identification and Propagation of Putative Transgenic Plants**

When the transgenic *E. crus-galli* seedlings reached a suitable size, leaf samples were collected for genomic DNA extraction. PCR amplification was performed using hygromycin gene-specific primers HygF1 (5′-GACCTGCCTGAAACCGAACTG-3′) and HygR1 (5′-CCCAAGCTGCATCATCGAAA-3′), and the products were analyzed by agarose gel electrophoresis. Plants showing a band of the expected size were identified as positive transformants, confirming successful integration of the target gene. These positive plants were then propagated in soil for further studies.

**callus induction medium**

| Component | Concentration |
| --- | --- |
| MS basal salts | 4.3 g/L |
| casein hydrolysate | 1 g/L |
| Proline | 690 mg/L |
| inositol | 350 mg/L |
| 2,4-D | 1 mg/L |
| CuSO_4_ | 1.25 mg/L |
| Maltose  Gellan Gum | 30 g/L  4.5 g/L |

*Note: Hygromycin (typically 50 mg/L) was added to the medium for positive callus selection. The exact concentration should be optimized according to preliminary tests on *E. crus-galli*. pH=5.8

**Differentiation Medium**

| Component | Concentration |
| --- | --- |
| MS basal salts | 4.3g/L |
| casein hydrolysate | 0.6g/L |
| NAA | 5mg/L |
| Sucrase  Gellan Gum | 30g/L  4.5 g/L |

*pH=5.8

**Rooting Medium**

| Component | Concentration |
| --- | --- |
| MS basal salts | 2.15g/L |
| casein hydrolysate | 0.6g/L |
| sucrase | 30g/L |
| Gellan Gum | 4.5 g/L |

*pH=5.8

**AAM Medium**

| Component | Concentration |
| --- | --- |
| AAM Base Salts with vitamins | 5.35 g/L |
| sucrase | 30 g/L |
| Gellan Gum | 8 g/L |

*pH=5.2
